# A specialized MreB-dependent cell wall biosynthetic complex mediates the formation of stalk-specific peptidoglycan in *Caulobacter crescentus*

**DOI:** 10.1371/journal.pgen.1007897

**Published:** 2019-02-01

**Authors:** Maria Billini, Jacob Biboy, Juliane Kühn, Waldemar Vollmer, Martin Thanbichler

**Affiliations:** 1 Faculty of Biology, Philipps-Universität, Marburg, Germany; 2 Max Planck Institute for Terrestrial Microbiology, Marburg, Germany; 3 Centre for Bacterial Cell Biology, Institute for Cell and Molecular Biosciences, Newcastle University, Newcastle upon Tyne, United Kingdom; 4 LOEWE Center for Synthetic Microbiology, Marburg, Germany; Universidad de Sevilla, SPAIN

## Abstract

Many bacteria have complex cell shapes, but the mechanisms producing their distinctive morphologies are still poorly understood. *Caulobacter crescentus*, for instance, exhibits a stalk-like extension that carries an adhesive holdfast mediating surface attachment. This structure forms through zonal peptidoglycan biosynthesis at the old cell pole and elongates extensively under phosphate-limiting conditions. We analyzed the composition of cell body and stalk peptidoglycan and identified significant differences in the nature and proportion of peptide crosslinks, indicating that the stalk represents a distinct subcellular domain with specific mechanical properties. To identify factors that participate in stalk formation, we systematically inactivated and localized predicted components of the cell wall biosynthetic machinery of *C*. *crescentus*. Our results show that the biosynthesis of stalk peptidoglycan involves a dedicated peptidoglycan biosynthetic complex that combines specific components of the divisome and elongasome, suggesting that the repurposing of preexisting machinery provides a straightforward means to evolve new morphological traits.

## Author summary

Bacteria show a variety of different cell shapes that are critical for survival in the environmental niche they inhabit. While the mechanisms generating the prototypic rod-shaped and coccoid morphologies have been studied intensively, only little is known about the processes that underlie the formation of more complex morphological features. The model organism *Caulobacter crescentus* is characterized by a polar stalk, which carries an adhesive organelle mediating surface attachment at its tip. This structure forms through the insertion of new cell wall material at its base and elongates considerably in phosphate-limited conditions. Our work reveals significant differences in the architecture of cell walls isolated from stalks and cell bodies, respectively, hinting at the existence of a stalk-specific cell wall biosynthetic apparatus. To identify components of this machinery, we systematically inactivated and localized proteins with a predicted enzymatic or regulatory function in cell wall biosynthesis in *C*. *crescentus*. Our results show that stalk formation is mediated by a pole-associated complex composed of proteins that have previously been identified as components of the cell elongation and cell division machineries. The stalk biosynthetic apparatus may thus have evolved through the repurposing of preexisting machinery, indicating that even complex morphological traits can emerge without the need for extensive changes to the complement of morphogenetic factors.

## Introduction

The shape of most bacteria is determined by a cell wall made of peptidoglycan (PG), a mesh-like heteropolymer that surrounds the cytoplasmic membrane and provides resistance against the internal osmotic pressure [1, 2]. The backbone of PG is formed by strands of alternating *N*-acetylglucosamine (GlcNAc) and *N*-acetylmuramic acid (MurNAc) subunits. These glycan chains are connected by short peptides that are attached to the MurNAc moieties, giving rise to a single elastic macromolecule known as the PG sacculus [3].

The PG meshwork needs to be continuously remodeled to allow for cell growth and division [4]. In Gram-negative bacteria, this task is achieved by a large and seemingly redundant set of PG synthesizing and degrading enzymes. Insertion of new cell wall material is initiated by the translocation of lipid-linked GlcNAc-MurNAc-pentapeptide precursors across the cytoplasmic membrane to the periplasm [5–7]. Glycosyltransferases (GTases) then incorporate the disaccharide units into preexisting glycan strands, while the L-Ala–D-Glu–L-Lys/*meso*-DAP–D-Ala–D-Ala pentapeptides of adjacent glycan strands are crosslinked by transpeptidases (TPases) [2, 8]. Depending on their domain structure, PG synthases can be classified as bifunctional GTases/TPases (class A PBPs), monofunctional TPases (class B PBPs) and monofunctional GTases [8].

The majority of TPases are DD-TPases, also known as penicillin-binding proteins (PBPs) [9]. These proteins catalyze the formation of D-Ala^4^–*meso*-DAP^3^ (4-3) crosslinks, in a reaction that releases the D-Ala^5^ moiety of the donor molecule [10]. Alternatively, crosslinks can also be formed between two *meso*-DAP^3^ residues (3–3 crosslinks), catalyzed by specific LD-TPases that use tetrapeptide side chains as donor moieties and release their terminal D-Ala^4^ residue to gain energy for the crosslinking reaction [11]. For PG to grow, cells require not only synthetic but also lytic enzymes that cleave bonds in the PG meshwork and thus make space for the insertion of new material [12]. Depending on their cleavage specificity, these so-called autolysins can be typically sorted into three main categories. Lytic transglycosylases act on the glycan strands and cleave the β-1,4-glycosidic bond between MurNAc and GlcNAc, leaving 1,6-anhydro-MurNAc as the terminal residue [13]. Amidases, by contrast, hydrolyze the amide bond between the peptide and the MurNAc moiety [12], whereas endo- and carboxypeptidases hydrolyze specific amide bonds within the peptides [8, 14].

The formation and degradation of PG need to be closely coordinated to prevent cell lysis [2, 15], a task that is presumably achieved by the assembly of synthetic and lytic enzymes into dynamic multi-protein complexes [16]. In the majority of rod-shaped bacteria, two of these complexes have been identified to date. The first one, called the elongasome (or Rod complex), mediates the dispersed incorporation of new PG along the lateral walls of the cell during the elongation phase. Its positioning is controlled by the actin-like protein MreB [17–19], which forms patch- or arc-like filaments that are attached to the inner face of the cytoplasmic membrane [20–24]. These structures move around the circumference of the cell and, thus, ensure even growth of the rod-shaped sacculus. In Gram-negative bacteria, their effect on the PG biosynthetic machinery is mediated by the transmembrane protein RodZ [25–27], which links MreB to a periplasmic complex containing the elongation-specific monofunctional TPase PBP2 [2, 28, 29]. Towards the end of the elongation phase, PG synthesis is taken over by a second complex, called the divisome [2, 30], which mediates pre-septal elongation and subsequent constriction of the PG sacculus at midcell. Its positioning and activity are regulated by FtsZ, a tubulin homolog that assembles into a dynamic ring-like structure at the future division site. This so-called Z-ring then recruits, directly or indirectly, all other components of the cell division machinery. The divisome includes a variety of PG synthases and hydrolases, among them the division-specific monofunctional TPase PBP3 [31], which act together to coordinately remodel the PG layer during the division process. Of note, in some species, MreB relocalizes to the division site before the onset of cell constriction but then moves back to the lateral walls as cytokinesis progresses, suggesting that the elongasome and divisome cooperate during certain stages of the division cycle [32, 33].

While the function of the elongasome and divisome and their roles in establishment of generic rod and coccoid morphologies have been studied intensively [2, 34], the mechanisms generating more complex cell shapes are still poorly understood. A model organism known for its distinctive morphological features is the alphaproteobacterium *Caulobacter crescentus* (henceforth *Caulobacter*) [35]. This species is characterized by a biphasic life cycle that involves two morphologically and physiologically distinct cell types. One of them, the swarmer cell, possesses a single polar flagellum mediating swimming motility. The stalked cell, by contrast, displays a tubular extension (stalk) whose tip carries an adhesive holdfast mediating surface attachment. Whereas the stalked cell undergoes repeated cycles of chromosome replication and cell division, the swarmer cell is arrested in G1 phase, searching its environment for nutrients. However, at a defined point in the cell cycle, it sheds its flagellum, starts to establish a stalk at the previously flagellated pole, and enters S phase. The cell then elongates, forms a new flagellum at the pole opposite the stalk, and finally divides asymmetrically to produce a stalked cell and a new swarmer cell [36]. The biological role of the *Caulobacter* stalk is still controversial, but it may serve as a spacer to elevate the cell above the substratum and thus enhance its access to nutrients [37]. Consistent with this idea, its length increases up to 20-fold under conditions of phosphate limitation [38].

In *Caulobacter* species, the stalk consists almost exclusively of the three cell envelope layers (inner membrane, cell wall and outer membrane) and does not contain any cytoplasm [35, 39]. Moreover, it is compartmentalized by large disc-like protein complexes, so-called crossbands, which are deposited at irregular intervals along its length, serving as non-selective diffusion barriers that physiologically separate the stalk envelope from the cell body [35, 40]. Formation of the stalk is driven by zonal incorporation of new cell wall material at the stalk base, as detected by the labeling of newly synthesized PG with tritiated glucose [38], radiolabeled D-cysteine [41], or fluorescently labeled D-alanine derivatives [42]. To date, various mutants have been identified that lack stalks under standard growth conditions [43–46]. However, in all cases, cells regained the ability to form stalks after transfer into phosphate-limited media, indicating that they suffered from a block in the cell cycle-regulated initiation of stalk formation rather than a defect in the underlying biosynthetic machinery. Depletion of MreB or the elongasome-specific GTase RodA [47], by contrast, impaired stalk elongation under all growth conditions [48]. Similar results were obtained after inhibition of the elongasome-specific TPase PBP2 [49] with the β-lactam antibiotic mecillinam. However, because of the severe global cell shape defects observed in these cases, it was difficult to conclude on a specific role of MreB, RodA and PBP2 in the stalk biosynthetic pathway. Finally, a moderate reduction in stalk length was observed for mutants lacking the cytoskeletal protein bactofilin A (BacA) or the BacA-associated class A PBP PbpC [50]. Together, these results suggest that components of the generic PG biosynthetic apparatus may be critical for stalk formation, but the precise composition of the machinery responsible for this process still remains elusive.

In the present study, we comprehensively investigate the mechanism of stalk formation, focusing on phosphate-limiting conditions to obtain a sensitive readout of the contributions that individual factors make to this process. We show that phosphate starvation induces a G0-like resting state that is characterized by the absence of key cell cycle regulators, including FtsZ. Comparing the muropeptide profiles of isolated stalk and cell body PG, we then identify significant differences in the composition of cell walls from these two compartments, suggesting that stalks are formed by specialized machinery with distinct biosynthetic properties. Systematic deletion and localization studies of cytoskeletal and PG biosynthetic proteins then indeed reveal a distinct set of factors involved in stalk elongation, which we characterize in detail with respect to their impact on PG composition and the spatial regulation of PG biosynthesis. Morphometric analysis of the corresponding mutants shows that these factors make varying and, in part specific, contributions to stalk and cell body elongation, indicating that these two modes of growth a mechanistically distinct. Finally, we identify MreB as a key component of the stalk biosynthetic complex and pinpoint a region on its surface that appears to be required for stalk formation but largely dispensable for elongasome-mediated lateral growth. Collectively, our results show that stalk formation represents a specialized growth process that is mediated by a composite complex including components of both the elongasome and divisome, with distinctive properties that clearly differentiate it from other PG biosynthetic machineries.

## Results

### Phosphate limitation arrests the cell cycle of *Caulobacter* in G1 phase

**Fig 1:**
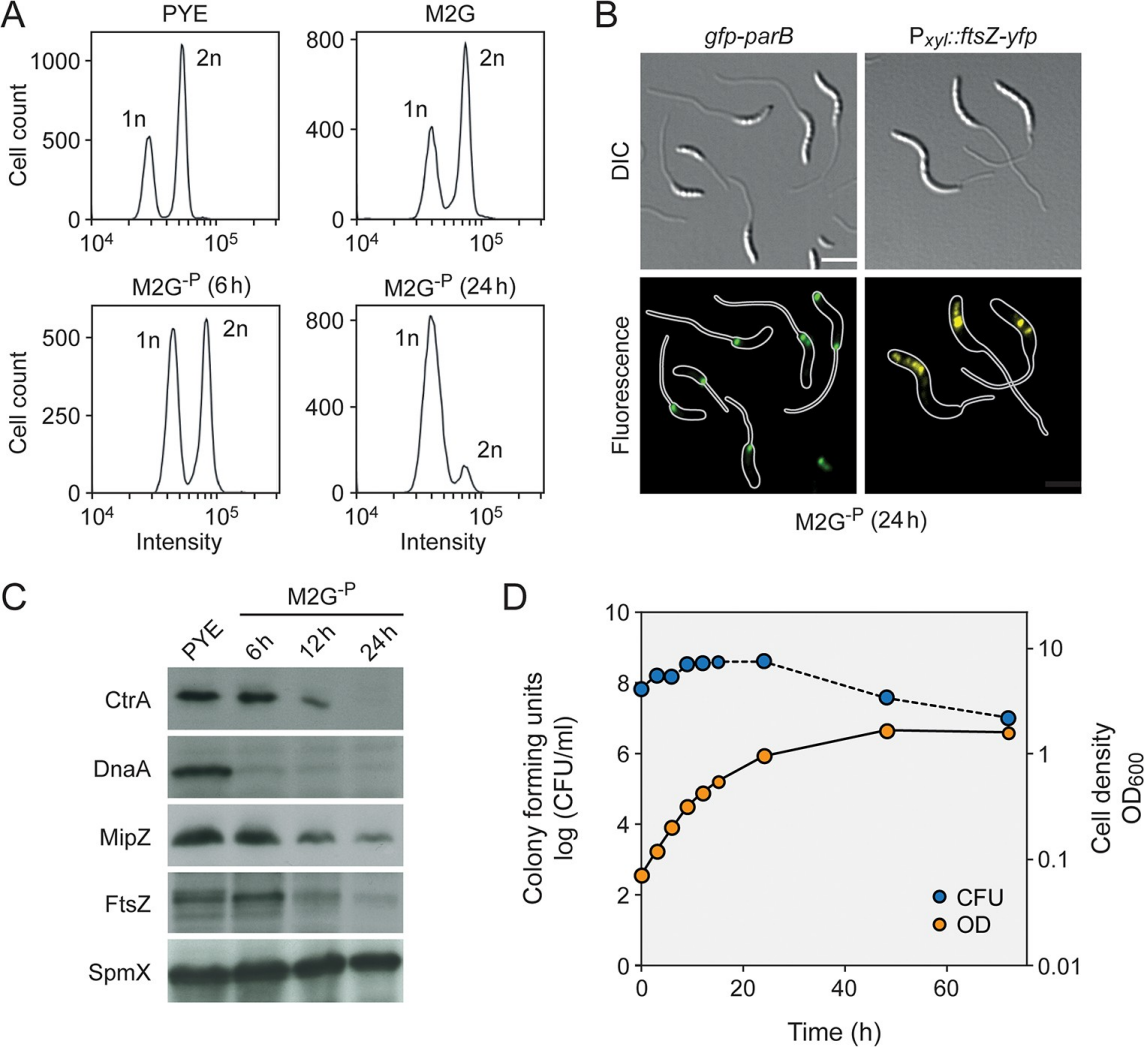
Progressive arrest of DNA replication and cell division under phosphate starvation. (**A**) DNA content of *C*. *crescentus* wild-type cells grown in PYE (rich medium, exponential phase), M2G (minimal medium, exponential phase), and M2G^-P^ (phosphate-lacking medium) for 12 h and 24 h. Cells were treated with 20 μg ml^−1^ rifampicin to prevent the reinitiation of replication prior to analysis by flow cytometry. (**B**) Subcellular localization of GFP-ParB and FtsZ-YFP in cells of strains MT199 (Pvan::Pvan*-ftsZ-yfp*) and MT174 (*parB*::*gfp*-*parB*) after 24 h of cultivation in M2G^-P^ medium. Synthesis of FtsZ-YFP was induced by addition of 50 μM vanillate 3 h prior to analysis (scale bar: 3 μm). (**C**) Changes in the levels of CtrA, DnaA, FtsZ, and MipZ over the course of phosphate starvation. Wild-type cells were grown in PYE, transferred into M2G^-P^ medium and subjected to Western blot analysis after 6 h, 12 h, and 24 h of incubation. A Western blot detecting SpmX served as a loading control. (**D**) Changes in the optical density (OD_600_) and viable-cell counts (CFU/ml) after transfer of a wild-type culture to M2G^-P^ medium.

Although the stimulatory effect of phosphate starvation on *Caulobacter* stalk elongation has been known for decades [38], the underlying regulatory mechanisms are still poorly understood. Prompted by the fact that stalk formation is tightly linked to cell cycle progression, we set out to investigate the effects of phosphate deprivation on central cellular processes such as DNA replication and cell division. First, flow cytometry was used to assess the replicational state of cells after transfer from standard to phosphate-free (M2G^-P^) medium. To this end, replication initiation was blocked with rifampicin and ongoing rounds of replication were allowed to finish. Previous work has shown that *Caulobacter* cells contain a single chromosome that is replicated only once per division cycle [51, 52]. Consistent with this finding, we observed that cells accumulated either one or two chromosome equivalents when grown in standard conditions, indicating that a large fraction of the population was in S-phase (**Fig 1A**). However, upon phosphate deprivation, DNA replication gradually ceased, with most cells arrested in G1 phase after 24 h of incubation. These data suggest that the lack of phosphate leads to a block in the cell cycle prior to S-phase, thereby preventing new rounds of chromosome replication. To support this conclusion, we visualized the number and positions of the chromosomal replication origins. In doing so, we made use of a fluorescently (GFP-) tagged derivative of the chromosome partitioning protein ParB, which interacts with specific motifs (*parS*) in the origin region [53, 54]. The expression of GFP-ParB thus typically results in the detection of either one or two foci, depending on the number of origin copies in the cell. Microscopic analysis revealed that most (~ 85%) cells exhibited a single ParB focus at the stalked pole when subjected to 24 h of phosphate starvation, indicating that they are arrested in G1 phase (**Fig 1B**). To clarify the reason for this G1 arrest, we analyzed the cellular levels of the replication initiator protein DnaA and the cell cycle master regulator CtrA, which act as positive and negative regulators of chromosome replication, respectively [52]. Interestingly, both proteins were rapidly depleted from the cells during phosphate starvation (**Fig 1C**), indicating that key drivers of the *Caulobacter* cell cycle are absent under this condition.

**Fig 2:**
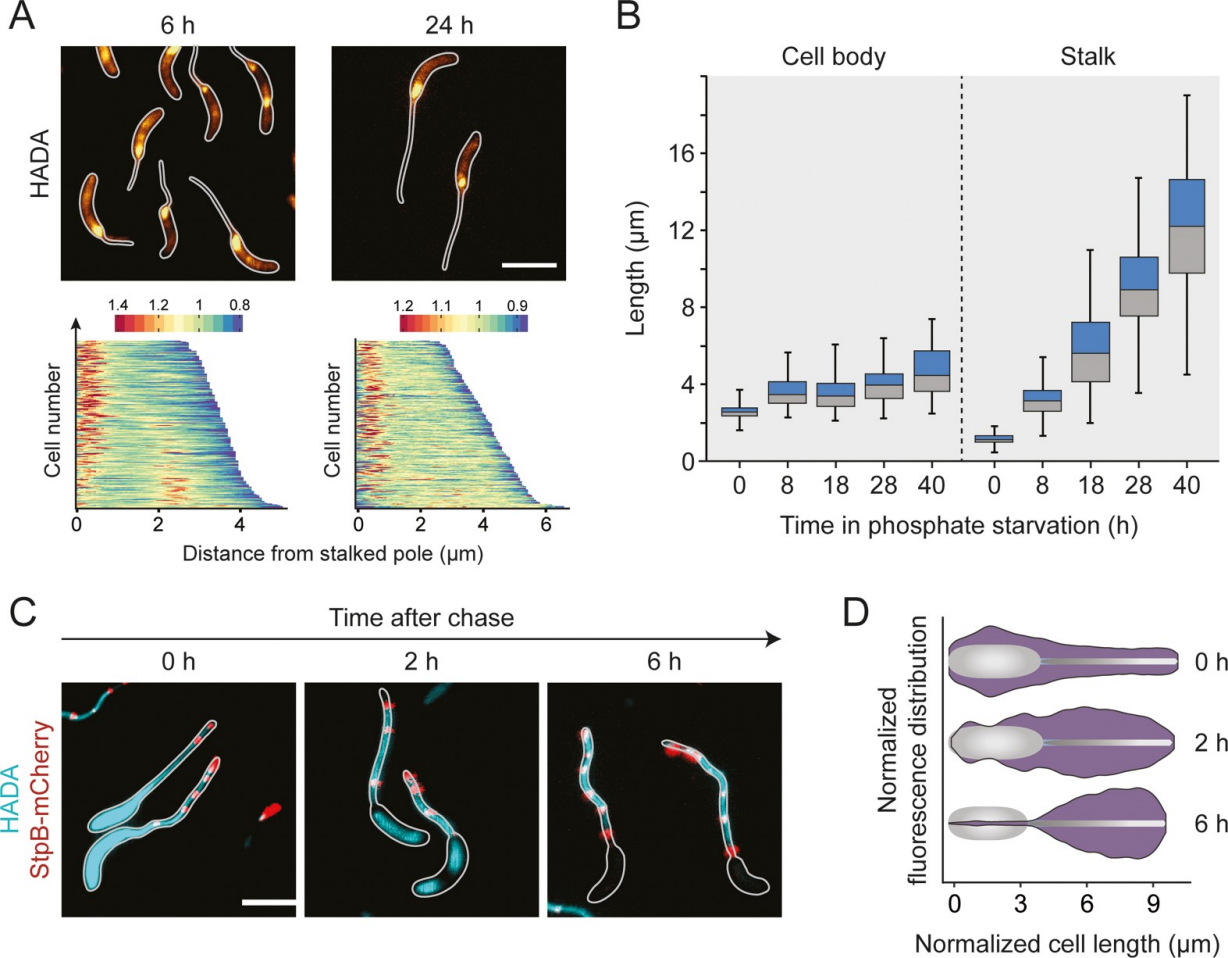
Reorganization of cell wall biosynthesis in the absence of phosphate. (**A**) Major growth zones of phosphate-starved wild-type cells. Cells were cultivated in M2G^-P^ medium for 6 h or 24 h and exposed to a short (2 min) pulse of HADA. The subcellular distribution of the fluorescence signals was quantified by demographic analysis of a random subpopulation of cells (n = 200). To generate the graphs, single-cell fluorescence profiles were sorted according to cell length and stacked on top of each other (scale bars: 3 μm). (**B**) Changes in stalk and cell body lengths during phosphate starvation. Wild-type cells were incubated in M2G^-P^ medium for 8 h, 18 h, 28 h, and 40 h prior to imaging. The data are shown as box plots, with the horizontal line indicating the median, the box the interquartile range, and the whiskers the 2^th^ and 98^th^ percentile (0 h: n = 108, 8 h: n = 100, 18 h: n = 100, 28 h: n = 105, 40 h: n = 102) (*** p < 10^−6^; t-test). See **S1 File** for the raw data. (**C and D**) Slow turnover of PG in the stalk compartment. Cells producing StpB-mCherry as a proxy for crossbands (SW30) were cultivated in M2G^-P^ medium for 18 h and exposed to HADA for an extended period of time (1.5 h). Subsequently, they were washed, transferred into fresh in M2G^-P^ medium and grown for 2 h, 4 h, and 6 h in the absence of the label (scale bars: 3 μm). To quantify the changes in HADA fluorescence overtime, fluorescence profiles were obtained from random subpopulations of cells (n = 200 per time point). The lengths of the profiles in each quintile of the cell length distribution were normalized to the maximum cell length in the respective quintile. Subsequently, the fluorescence intensities were averaged and used to generate violin plots. Shown is a representative part of the data depicting the fluorescence distributions in the fourth quintile at each of the time points (**D**). The full analysis is presented in **S2C Fig**.

To correlate changes in cell cycle progression with the growth behavior of cells, we monitored changes in cell mass and number after a shift to phosphate-limiting conditions. Interestingly, the optical density of cultures kept increasing exponentially for more than 10 h and only leveled off after ~ 50 h of incubation (**Fig 1D**), suggesting that cells made use of internal phosphate storage compounds to compensate for the lack of an external phosphate source. Consistent with the detection of DNA replication events (**Fig 1A**), cells still multiplied during the initial exponential phase. However, after longer starvation periods (> 24 h), the viable-cell count started to decline, whereas the cell mass still increased, likely due to continued elongation of the cell bodies and stalks in the absence of cell division events (see **Fig 2B**). Western blot analysis indeed revealed that the essential cell division protein FtsZ was depleted from the cells upon phosphate starvation (**Fig 1C**). The same was true for the cell division regulator MipZ, an inhibitor of FtsZ polymerization that limits Z-ring formation to the midcell region [54]. In line with these findings, an FtsZ-YFP fusion induced after prolonged phosphate starvation formed multiple foci in the vicinity of the stalk-distal pole instead of a defined midcell band (**Fig 1B**), indicating the absence of a functional and properly localized Z-ring [54]. Notably, FtsZ was never observed at the stalk base, supporting the previous notion that it does not play any role in stalk formation [54].

Taken together, our results demonstrate that phosphate starvation arrests the *Caulobacter* cell cycle in a G1-like phase, thereby stalling DNA replication and cell division until phosphate becomes available again.

### Phosphate starvation induces a distinct pattern of PG synthesis

Phosphate starvation induces *Caulobacter* to enter a non-replicative state in which cells continue to elongate their cell body and stalk. To investigate this atypical mode of growth, we set out to visualize sites of active PG biosynthesis using the fluorescent D-amino acid 7-hydroxy-coumarin-amino-D-alanine (HADA) [42, 55] as a tracer. As a control, we initially analyzed the growth dynamics of cells growing in phosphate-replete medium. To this end, cells were synchronized and then pulse-labeled with HADA at different stages of the cell cycle. Consistent with previous results [41], we observed disperse incorporation of new cell wall material before the onset of cell division, followed by zonal growth at midcell during the constriction phase (**S1 Fig**). Moreover, concurrent with the switch from disperse to zonal growth, an additional intense focus of fluorescence appeared at one of the cell poles, reflecting the establishment and outgrowth of the stalk. This polar signal faded gradually as the cell cycle progressed and was no longer detectable in late pre-divisional cells. Thus, HADA reliably detected all known growth zones in *Caulobacter* cells.

Next, we used HADA labeling to determine the pattern of PG synthesis under phosphate-limiting conditions (**Fig 2A**). After 6 h of incubation in phosphate-free medium, most cells showed a bright fluorescent patch at the stalked pole as well as a faint disperse signal extending throughout the rest of the cell body. Cells longer than ~ 4 μm often displayed an additional bright focus at their center, which could reflect FtsZ-dependent zonal growth or cell division, consistent with the observation that the viable-cell counts still increased in the early phase of starvation (**Figs 1D** and **S2A**). Interestingly, the intensity of the polar signal decreased considerably upon appearance of a midcell focus, suggesting that the machineries mediating stalk formation and cell division may compete with each other for at least some of their components (**Fig 2A**). After longer starvation periods (>18 h), midcell foci were almost undetectable, and HADA fluorescence was largely limited to the stalk base, which correlates with the lack of cell division events at this time point. Notably, the intensity of the polar signal decreased slightly during long-term incubation (**S2B Fig**), although the rate of stalk elongation remained constant at all time points (**Fig 2B**). The increase in cell body length, by contrast, was most pronounced during the early phases of starvation, when cells still showed midcell HADA foci, suggesting that it may, at least in part result, from FtsZ-mediated zonal growth at the cell center. Collectively, phosphate starvation induces a switch in the pattern of PG synthesis that ultimately limits cell growth to the stalked cell pole.

Our and previous labeling studies suggest that stalk formation is driven by the insertion of new cell wall material at the stalk base [38, 41, 42]. To determine whether stalk PG is still subject to modification or turnover, phosphate-starved *Caulobacter* cells were incubated with HADA for an extended period of time (1.5 h). After this treatment, staining was observed throughout the entire cell envelope (**Fig 2C**), including distal segments of the stalk that were clearly formed prior to the start of the labeling procedure (considering a stalk elongation rate of 0.28 ± 0.03 μm/h; see **Fig 2B**). Given that HADA is likely incorporated by the action of periplasmic DD- or LD-TPases [42, 56, 57], this finding indicates the presence of transpeptidase activity in the stalk compartment that mediated the addition of HADA to preexisting PG independently of the pole-associated biosynthetic complex. After transfer of the cells to HADA-free medium, fluorescence was rapidly lost in the cell bodies and in the basal region of the stalk, whereas it was stably retained in the distal stalk segments, indicating that stalk PG is not turned over at significant rates (**Fig 2C** and **2D**). Notably, the same behavior was observed for a strain lacking crossbands. The differences in the behavior of cell bodies and stalks may thus not result from restrictions in the diffusion of envelope-localized PG biosynthetic enzymes but rather from the retention of these enzymes at the stalk base, possibly due to the lack of cytoplasm in the stalk compartment.

### The stalk and cell body show different PG architectures

**Fig 3:**
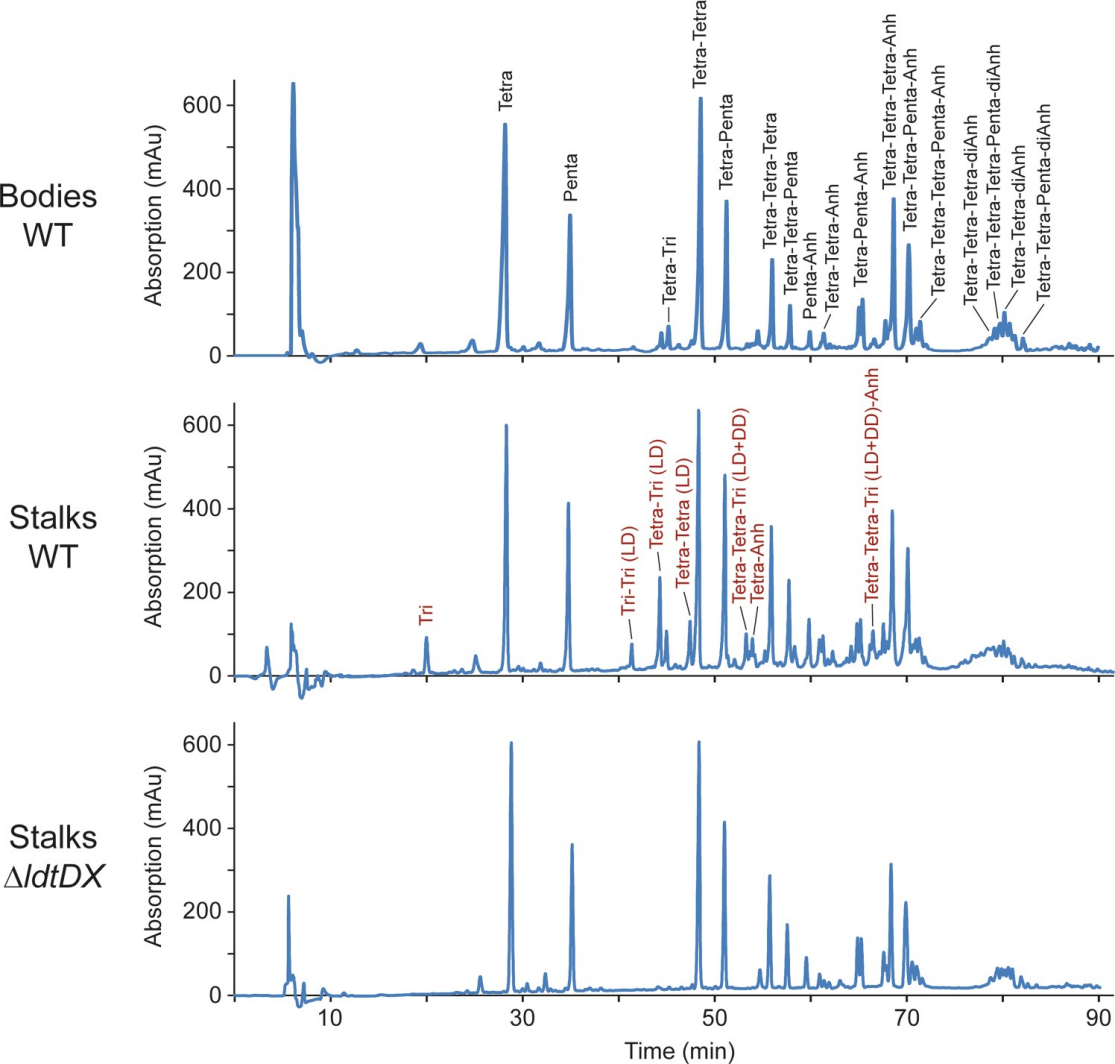
Differential composition of cell body and stalk peptidoglycan. Shown are the HPLC profiles of muropeptides obtained from the cell body and stalk fractions of stains NA1000 (WT) and AZ138 (Δ*ldtD* Δ*ldtX*) after growth in M2G^-P^ medium for 24 h. In the first panel, the identities of the most abundant muropeptides are given in black. In the second panel, products that are specifically enriched in the stalk fraction are indicated in red. Abbreviations: Tri: Glc*N*Ac–Mur*N*Ac(r)–L-Ala–D-Glu–mDap; Tetra: Glc*N*Ac–Mur*N*Ac(r)–L-Ala–D-Glu–mDap–D-Ala; Penta: Glc*N*Ac–Mur*N*Ac(r)–L-Ala–D-Glu–mDap–D-Ala–D-Ala; Anh: 1,6-anhydro-Mur*N*Ac; DD: mDap–D-Ala crosslink; LD: mDap–mDap crosslink.

The distinct mode of growth involved in stalk formation opens the possibility that there may be compositional differences between the PG layers encompassing the cell body and stalk compartments. To address this issue, phosphate-starved cells were agitated vigorously to shear off stalks from the cell bodies. After separation of the two compartments by differential centrifugation (**S3 Fig**), PG was isolated from each of the fractions and subjected to muropeptide analysis. Interestingly, stalk PG contained a high proportion of 3–3 crosslinked peptides and non-crosslinked tripeptides (resulting from the cleavage of 3–3 bonds), whereas these muropeptide species were barely detectable in the cell body samples (**Fig 3** and **S1 Table**). Similarly, the total fraction of crosslinked peptide side chains was significantly higher in stalk PG, mostly because of a higher proportion of trimeric muropeptides. The glycan chain lengths, by contrast, did not vary between the two compartments. Collectively, these findings indicate that the PG layers of stalks and cell bodies differ in both the type and extent of peptide crosslinks.

**Fig 4:**
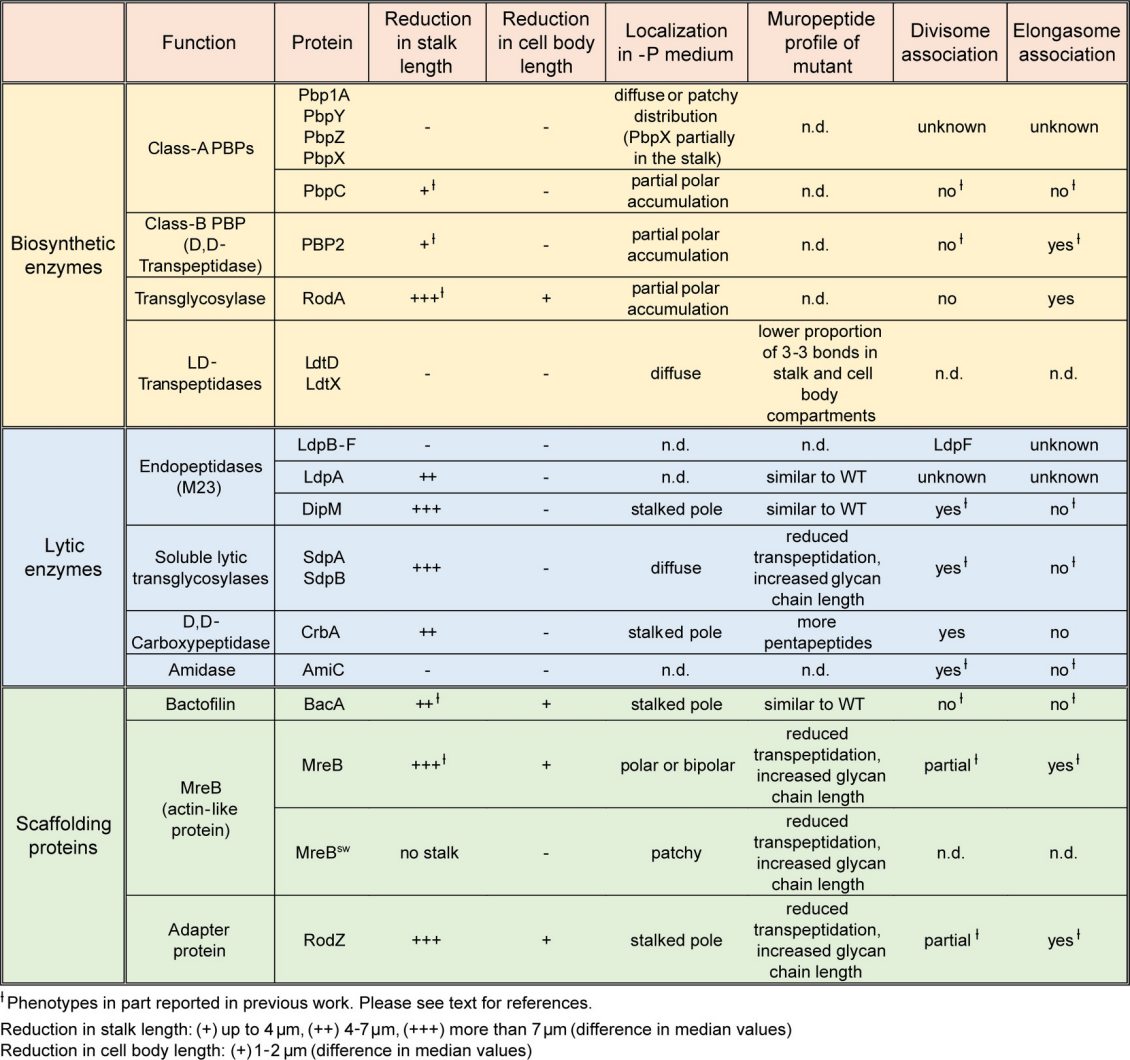
Summary of the results obtained in this study.

Previous work has shown that 3–3 crosslinks are generated by LD-TPases, which are characterized by a conserved YkuD domain [11]. The *Caulobacter* genome contains two so-far uncharacterized open reading frames, CC_1511 and CC_3744, which encode proteins with this signature domain (now referred to as LdtD and LdtX, respectively). To determine how these factors contribute to the distinctive composition of the stalk cell wall, we generated a strain carrying in-frame deletions in both the *ldtD* and *ldtX* gene and analyzed the composition of PG purified from its stalk and cell body compartments. In both samples, 3–3 crosslinked peptides and non-crosslinked tripeptides were virtually undetectable (**Fig 3** and **S1 Table**), indicating that the formation of these muropeptide species is linked to the activity of the two predicted LD-TPases. Notably, however, the total fraction of crosslinked peptides barely changed in either of the compartments, because the loss of 3–3 crosslinks was compensated by a proportional increase in the fraction of 4–3 crosslinks. Thus, LD-TPase activity is not the main factor responsible for the elevated degree of crosslinking detected in stalk PG (see **Fig 4** for a summary of the key results obtained in this study).

The stalk is physiologically separated from the cell body, because it is devoid of cytoplasm and contains crossband complexes that block the exchange of periplasmic and membrane proteins [40]. It was conceivable that crossbands could help establish the differences in the PG composition observed for the two compartments, for instance by facilitating the establishment of distinct pools of PG biosynthetic enzymes or blocking the diffusion of lipid II into the stalk structure. To test this idea we determined the muropeptide profile of stalk and cell body PG isolated from a crossband-less strain (Δ*stpAB*, SW51). Notably, we still observed a higher content of 3–3 crosslinks and a higher total proportion of crosslinked peptides in stalk PG (**S1 Table**). Similar to the differences in PG turnover (**Fig 2C**), this characteristic thus appears to be independent of the presence of crossbands.

### Stalk formation requires DD- but not LD-transpeptidase activity

**Fig 5:**
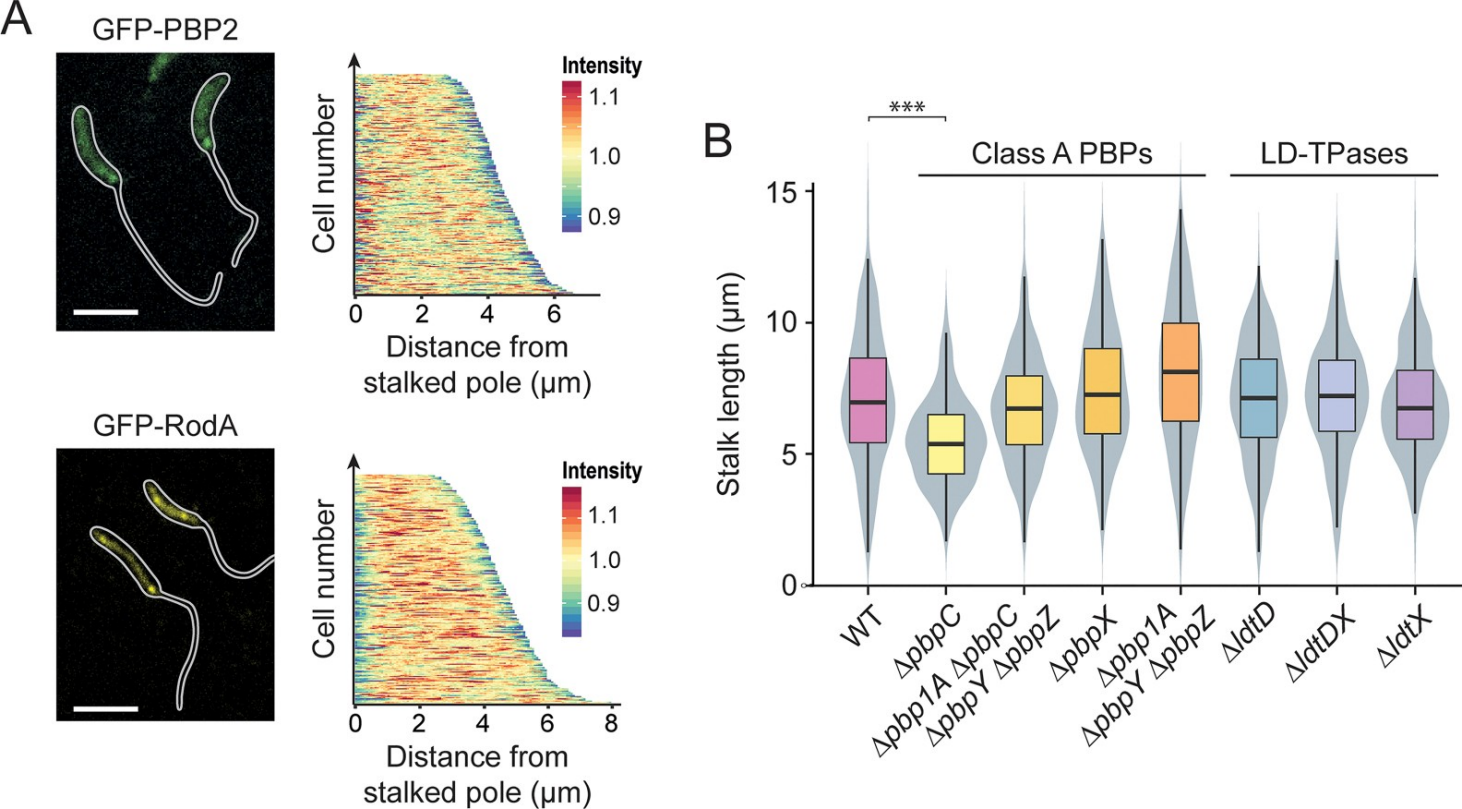
Participation of PG biosynthetic enzymes in stalk elongation. (**A**) Localization of GFP-PBP2 and GFP-RodA in strains MAB244 (*pbp2*::*gfp-pbp2*) and MAB405 (Pxyl::Pxyl*-gfp-rodA*), respectively, after 24 h of cultivation in M2G^-P^. The demograph shows the fluorescence profiles of a random subpopulation of cells sorted according to cell length (GFP-PBP2: n = 227, GFP-RodA: n = 205). (**B**) Distribution of the stalk lengths in populations of mutants lacking specific PG synthases. Shown are the results obtained for MT286 (Δ*pbpC)*, JK305 (Δ*pbpA1* Δ*pbpC* Δ*pbpY* Δ*pbpZ*), KK1 (Δ*pbpX*), KK12 (Δ*pbp1A* Δ*pbpY* Δ*pbpZ*), AZ137 (Δ*ldtD*), AZ138 (Δ*ldtDX*), and AZ140 (Δ*ldtX*) after 24 h of growth in M2G^-P^ medium. Data are represented as box plots, with the horizontal line indicating the median, the box the interquartile range and the whiskers the 2^nd^ and the 98^th^ percentile (n = 208 per strain). In addition rotated kernel density plots (grey) are depicted for each dataset to indicate the distribution of the raw data (*** p < 10^−6^; t-test). See **S1 File** for the raw data.

Stalk formation involves a growth process that is distinct from the disperse and zonal incorporation of PG mediated by the elongasome or division complex, respectively. To determine the composition of the underlying machinery, we systematically analyzed all predicted PG biosynthetic proteins encoded in the *Caulobacter* genome for their contribution to stalk elongation under phosphate-limiting conditions (see **Fig 4** for a summary). In doing so, we initially focused on enzymes with PG synthase activity, including RodA, mono- and bifunctional PBPs and LD-TPases. Previous studies have shown that the depletion of RodA or the inhibition of the monofunctional DD-TPase PBP2 with mecillinam largely abolished the synthesis of stalks in phosphate-replete media, although these treatments concomitantly induced severe morphological defects in the cell body [48, 49]. To verify these results, we determined the consequences of RodA depletion on stalk elongation during phosphate limitation (**S4 Fig**). The absence of RodA indeed led to a severe decrease in both stalk and cell body length. Similar tendencies were observed for mecillinam-treated cells, although the effects were generally less pronounced. These results indicate a global morphogenetic function of RodA and PBP2 in phosphate-starved cells. To further investigate these proteins, we generated functional fluorescent protein fusions [58] and analyzed their localization in phosphate-limited media. Consistent with a global role in PG biosynthesis, both GFP-RodA (**S9 Fig**) and GFP-PBP2 formed large patches that were distributed throughout the cell bodies. Moreover, both proteins frequently formed a faint but distinct focus at the stalked pole, particularly in cells with clearly elongated (> 4 μm) bodies (**Fig 5A**). Thus, RodA and PBP2 appear to be partly associated with the stalked pole, where they likely cooperate to mediate stalk formation.

Previous work has also implicated bifunctional PBPs in stalk elongation, largely based on the analysis of mutants lacking one of these proteins [50, 59]. To verify and extend these results, we analyzed strains carrying single or multiple mutations in the PBP-encoding *pbpY*, *pbp1A*, *pbpC*, *pbpX*, and *pbpZ* genes [59, 60]. Our results confirm that deletion of *pbpC* led to a moderate reduction in stalk length, whereas the absence of any other PBP, either alone or in combination, did not have any effect (**Fig 5B**). However, as observed under standard growth conditions [59, 60], at least one bifunctional PBP was required for viability during phosphate starvation (**S5A Fig**). In line with the results of the deletion studies, localization analyses revealed that none of the bifunctional PBPs except for PbpC accumulated at the stalked pole, indicating that these proteins may not be specifically associated with the stalk biosynthetic machinery (**S5B Fig**). Notably, however, PbpX appeared enriched in the stalk compartments, but the significance of this observation remains unclear.

Finally, we analyzed the role of the two predicted LD-TPases LdtD and LdtX in stalk formation. Although these proteins make a significant contribution to PG crosslinking in the stalk compartment (**Fig 3**), their inactivation did not have any apparent phenotypic effect (**Fig 5B**). LD-TPase activity may thus not contribute to the establishment of the stalk structure per se but rather have an accessory function that serves to modify the biophysical properties of the PG layer. Localization studies indicate that LdtD and LdtX do not accumulate at the stalk base, suggesting that they may act independently of the polar stalk biosynthetic machinery (**S5C Fig**).

### Components of the autolytic machinery are critical for proper stalk formation

**Fig 6:**
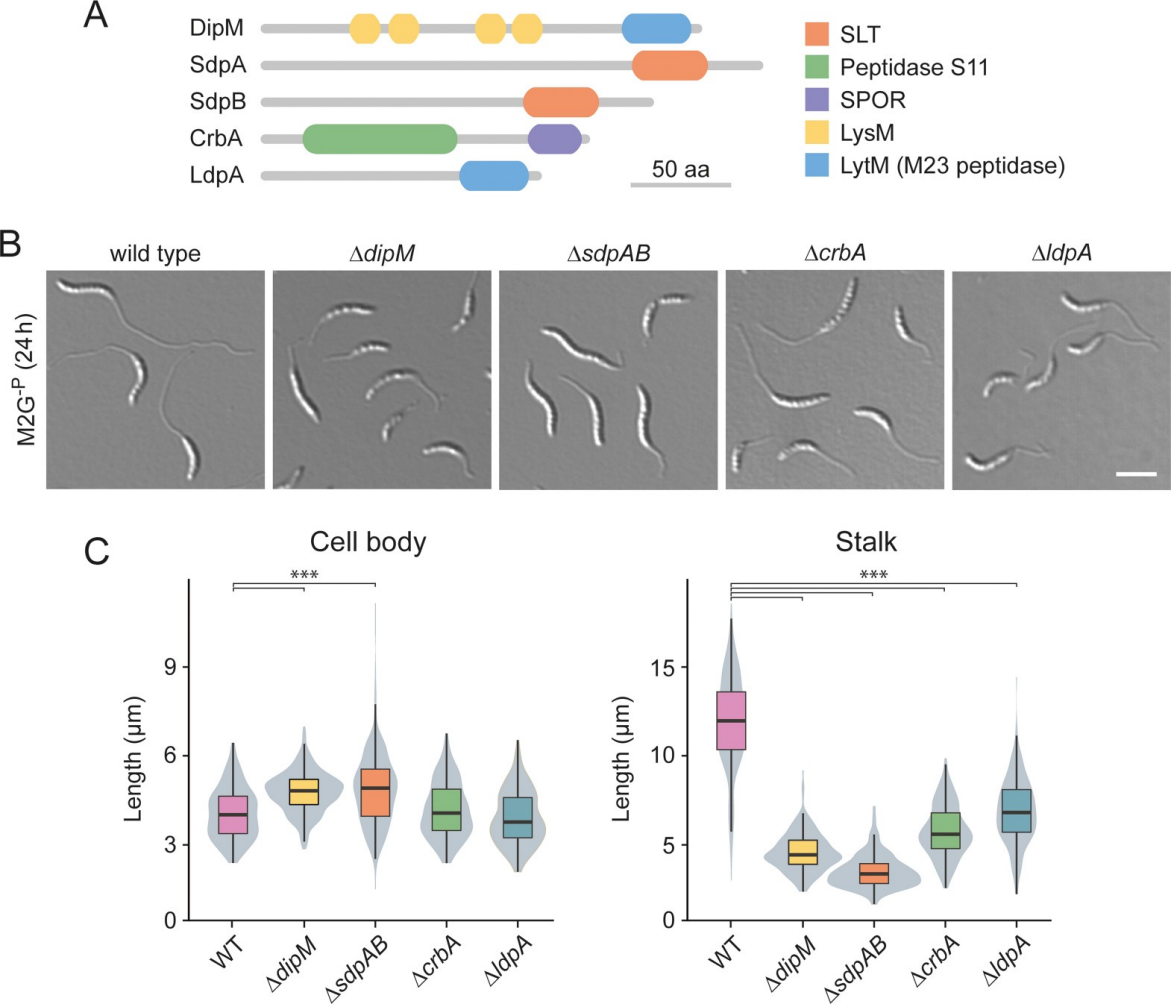
Participation of autolytic factors in stalk elongation. (**A**) Domain structure of selected components of the autolytic machinery of *C*. *crescentus*. (**B**) DIC micrographs of mutant cells exhibiting a stalk elongation defect. Shown are strains MT258 (Δ*dipM*), AZ22 (Δ*sdpAB*), AM376 (Δ*crbA*), and AM364 (Δ*ldpA*) in comparison to NA1000 (WT) after 24 h of cultivation in M2G^-P^ medium. (**C**) Distribution of the cell body and stalk lengths in populations of strains MT258, AZ22, AM376, and AM364 after growth in M2G^-P^ for 24 h. The values obtained are shown as box plots, with the horizontal line indicating the median, the box the interquartile range and the wiskers the 2^nd^ and the 98^th^ percentile (n = 208 per strain). In addition rotated kernel density plots (grey) are depicted for each dataset to indicate the distribution of the raw data (*** p < 10^−6^; t-test). See **S1 File** for the raw data.

Apart from PG synthases, stalk formation must also involve autolytic enzymes that cleave the PG sacculus and, thus, enable the insertion of new cell wall material at the stalk base. However, to this point, the nature of the factors involved has remained unknown. To address this issue, we systematically screened mutants lacking one or multiple predicted PG hydrolases for defects in stalk growth under phosphate-limiting conditions. The enzymes tested included all LytM-like and NlpC/P60-like endopeptidases, AmiC-like and CHAP domain-containing amidases, soluble and membrane-bound lytic transglycosylases, and carboxypeptidases identified in the *Caulobacter* genome (**S2 Table**). In most cases, the lack of single factors and even the absence of whole enzyme families had no apparent effect on stalk length (**S6 Fig**). Four strains, however, displayed obvious morphological defects (**Fig 6**; see **S7 Fig** for the phenotypes in PYE medium). One of them was a mutant lacking the protein DipM, a catalytically inactive LytM-like endopeptidase homolog that was previously shown to be critical for proper PG remodeling during cell division [61–63]. The absence of DipM led to a severe reduction in stalk length, combined with the formation of branches within the stalk structure or the establishment of multiple stalks, often emanating from the same pole (**Figs 6B, 6C** and **S8**). Even shorter stalks were observed in the combined absence of the soluble lytic transglycosylases SdpA and SdpB (**Fig 6B** and **6C**), two proteins previously found to be associated with the divisome complex [64]. Apart from its aberrant morphology, the Δ*sdpAB* mutant frequently showed membrane blebs that were associated with the residual stalk structures, suggesting a defect in membrane attachment or homeostasis (**S8 Fig**). Milder effects on stalk length were caused by inactivation of the divisome-associated carboxypeptidase CrbA (Billini *et al*, unpublished) or the LytM-like endopeptidase LdpA, a thus-far uncharacterized protein encoded in an operon with the polarly localized scaffolding protein bactofilin A (BacA) [50, 65] (**Figs 6B, 6C** and **S8**). Importantly, despite their stalk elongation defects, none of the four deletion strains showed a significant reduction in cell length (**Fig 6C**), indicating that stalk and cell body growth are mechanistically distinct processes that proceed independently of each other.

**Fig 7:**
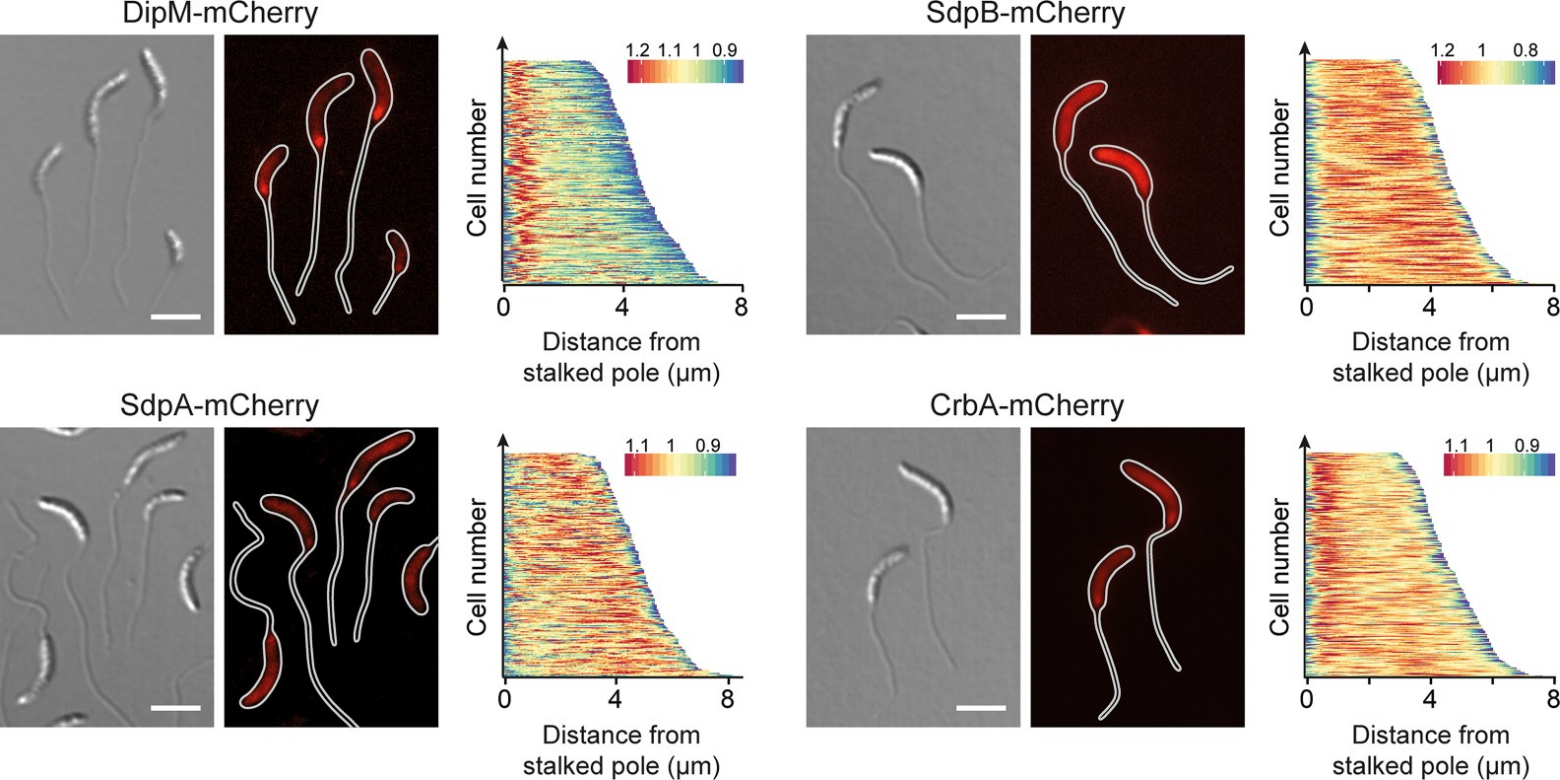
Localization of autolytic factors in phosphate-starved cells. Shown are the localization patterns of SdpA-mCherry (AM480, Pxyl::Pxyl-*sdpA-mCherry*), CrbA-mCherry (MAB247, Pxyl::Pxyl-*crbA-mCherry*), DipM-mCherry (AM208, Pxyl::Pxyl-*dipM-mCherry*), and SdpB-mCherry (AZ127, Pxyl::Pxyl-*sdpB-mCherry*) in cells cultivated for 24 h in M2G^-P^ medium (scale bars: 3 μm). Synthesis of the fluorescent protein fusions was induced for 3 h (for DipM, SdpA, and CrbA) or 2 h (for SdpB) with 0.3% xylose prior to analysis. The demographs next to the images show the fluorescence profiles of a random subpopulation of cells sorted according to cell length (n = 200 for each strain).

To further investigate the functions of the five autolytic factors identified in the mutational screen, we generated fluorescently (mCherry-) tagged derivatives of these proteins and analyzed their localization patterns under conditions of phosphate starvation (**Fig 7**). Both the DipM and CrbA fusions accumulated at the stalk base and may, thus, be specifically associated with the polar stalk biosynthetic machinery. The SdpA and SdpB fusions, by contrast, were distributed throughout the cell envelope, suggesting that the two proteins may either act independently of the polar complex or associate with it in a very transient manner. Unlike the other proteins analyzed (**S9 Fig**), LdpA-mCherry was quantitatively cleaved at the junction between the two fusion partners, preventing further analysis.

**Fig 8:**
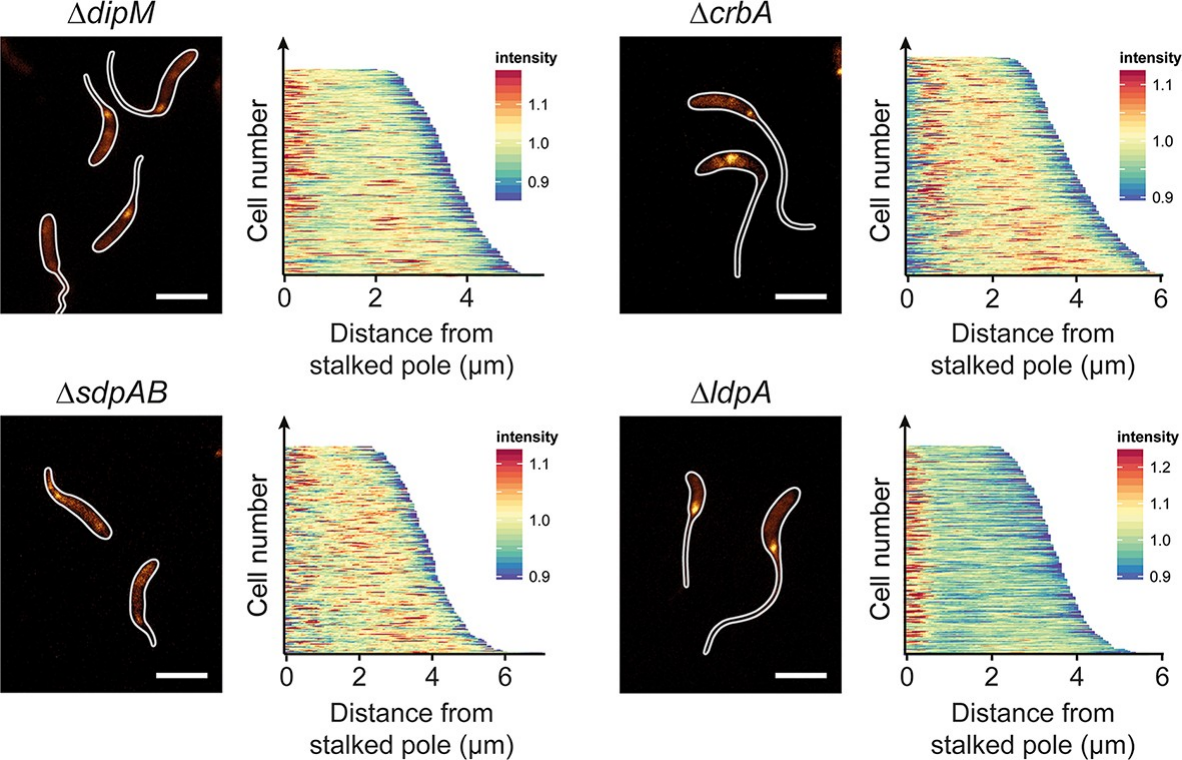
Cell wall biosynthesis in mutants with defects in the autolytic machinery. Shown are fluorescence images of strains MT258 (Δ*dipM*), AZ22 (Δ*sdpAB*), AM376 (Δ*crbA*), and AM364 (Δ*ldpA*) after 24 h of incubation in M2G^-P^ medium and subsequent HADA staining (2 min). The distribution of HADA fluorescence was quantified by demographic analysis of a random subpopulation of cells (n = 200 for each strain) (scale bars: 3 μm).

In order to determine how the absence of the different autolytic factors influenced the pattern of PG biosynthesis, mutants lacking these proteins were grown in phosphate-limiting conditions and subjected to HADA staining (**Fig 8**). Consistent with their relatively mild stalk elongation defect, Δ*ldpA* cells still displayed a pattern similar to that of the wild-type strain. In the Δ*dipM* and Δ*crbA* strains, by contrast, the polar signals were much fainter and new cell wall material was often incorporated at non-polar sites. An even more pronounced effect was observed in the Δ*sdpAB* mutant, which virtually lacked polar foci and instead showed patchy or even HADA fluorescence throughout the cells. Thus, the severity of the stalk elongation defect scales with the loss in polar PG biosynthesis.

To obtain more detailed insight into the effects of the different mutations on the structure of the PG layer, we isolated whole-cell sacculi from wild-type and mutant cells after prolonged (24 h) phosphate starvation and subjected them to muropeptide analysis (**S3 Table**). For the wild-type strain, whole-cell sacculi gave similar results as PG from isolated from a cell body fraction (compare **Fig 3** and **S1 Table**), indicating that the characteristic features of stalk PG are largely obscured by the excess of cell body PG in the whole-cell preparations. Interestingly, there were hardly any differences between the muropeptide profiles obtained under phosphate-limiting (**S3 Table**) and phosphate-replete [66] conditions. The average composition of cell body PG thus appears to be independent of the phosphate supply. Among the mutant strains, Δ*ldpA* cells showed essentially the same average PG composition as the wild-type strain. The same was true for the Δ*dipM* mutant, with exception of a significant increase in the proportion of non-crosslinked tetrapeptides (**S3 Table**), which could indicate an elevated level of endopeptidase and/or carboxypeptidase activity. The muropeptide profiles of the remaining strains, by contrast, showed marked global changes. In line with the notion that CrbA acts as a carboxypeptidase, removing the terminal D-Ala residue of pentapeptide side chains (Billini *et al*, unpublished), the Δ*crbA* mutant displayed a considerable decrease in the total content of tetrapeptides that was accompanied by a proportional increase in the content of pentapeptide-containing muropeptide species (**S3 Table**). In Δ*sdpAB* cells, on the other hand, the average glycan chain length increased from 7 to 9.4 disaccharide units, consistent with the loss of lytic transglycosylase activity. Surprisingly, the mutant cells additionally showed a severe reduction in the degree of crosslinkage. At the same time, their total content of pentapeptide side chains was reduced, whereas the proportion of tripeptide side chains was considerably elevated (**S3 Table**). These results suggest that the lack of SdpAB leads to reduced transpeptidation or, more likely, elevated endopeptidase activity.

Collectively, our results show that several components of the autolytic machinery are critical for proper PG remodeling during stalk formation, with some of them localizing to the stalk base under phosphate-limiting conditions (see **Fig 4** for a summary). Notably, most of the proteins, including DipM, SdpA, SdpB and CrbA, are associated with the cell division apparatus under standard growth conditions [61–64] (M. Billini, unpublished), suggesting parallels in the mechanisms that reshape the PG layer during cell constriction and stalk growth.

### Stalk formation depends on the presence of scaffolding proteins

**Fig 9:**
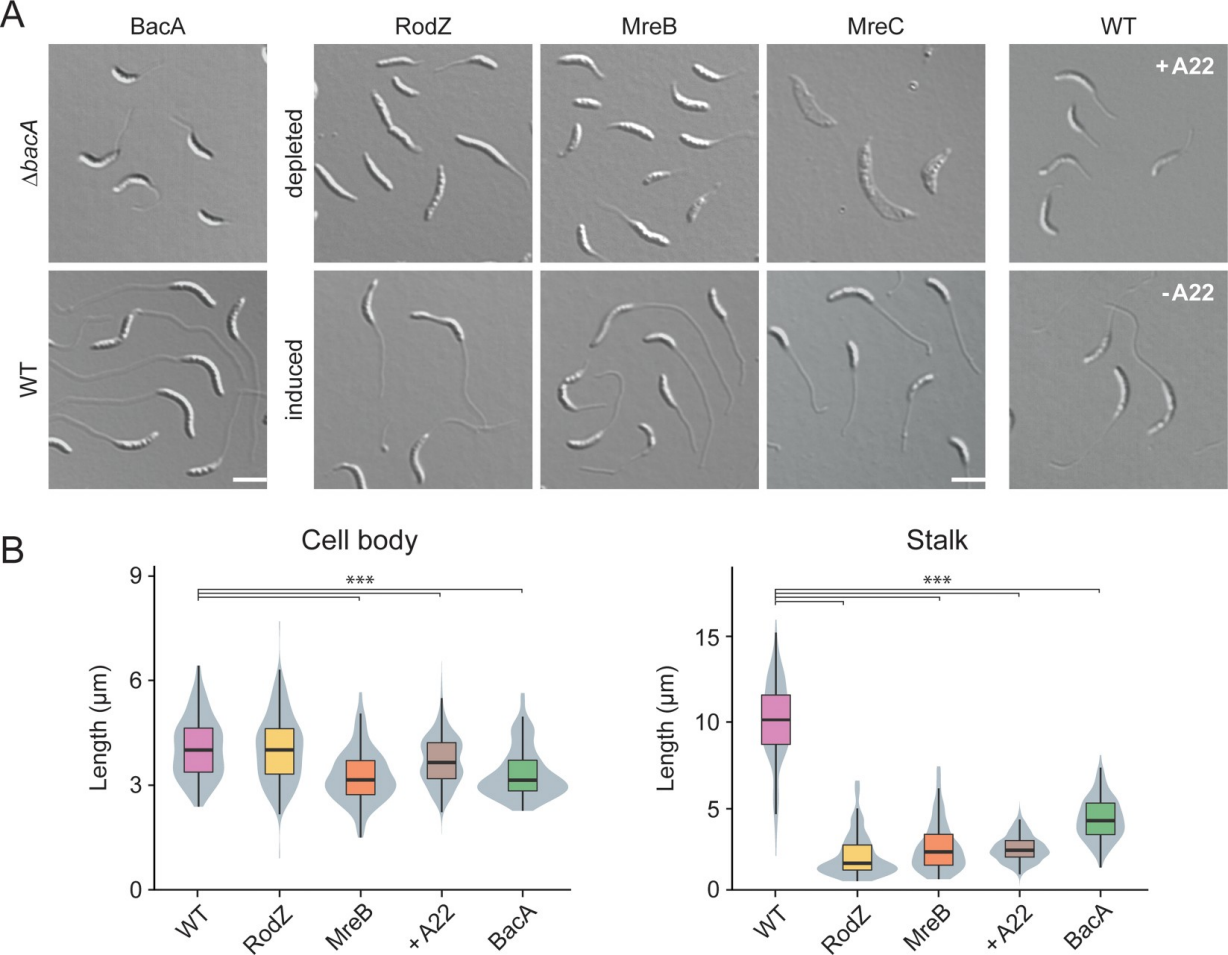
Role of scaffolding proteins in stalk elongation. (**A**) DIC images of cells lacking single scaffolding proteins. The strains analyzed were LS4275 (Δ*mreC* Pxyl::Pxyl*-mreC*), LS3809 (Δ*mreB* Pxyl::Pxyl*-mreB*), CJW2747 (Δ*rodZ*::*Ω* Pxyl::Pxyl*-rodZ*), MT257 (Δ*bacA*). Strain NA1000 (WT) is shown as a wild-type control. Strains LS4275, LS3809, and CJW2747 were grown to exponential phase PYE medium containing the inducer xylose. Subsequently, the cells were washed, grown for another 7 h in PYE medium with or without inducer and then incubated for 24 h in M2G^-P^ medium with or without inducer prior to imaging by DIC microscopy. Strain MT257 was grown to stationary phase in PYE medium, diluted (1:20) into M2G^-P^ medium, and grown for 24 h prior to analysis. Strain NA1000 (WT) was grown in M2G^-P^ medium. After 9 h, the cultures were supplemented with A22 at a final concentration of 10 μg/ml and incubated for additional 15 h prior to imaging (scale bar: 3 μm). (**B**) Distribution of cell body and stalk lengths in populations of WT NA1000 and depleted strains LS4275, LS3809, CJW2747, and MT257 grown as described in (A). The data are shown as box plots, with the horizontal line indicating the median, the box the interquartile range and the wiskers the 2^nd^ and the 98^th^ percentile (n = 208 per strain). In addition rotated kernel density plots (grey) are depicted for each dataset to indicate the distribution of the raw data (*** p < 10^−6^; t-test). See **S1 File** for the raw data.

Polymer-forming scaffolding proteins are critical for the regulation of many growth processes in bacteria [4, 67], suggesting that this group of proteins may also play a critical role in stalk formation. Previous work has indeed implicated the bactofilin homolog BacA in stalk biogenesis [50]. Re-analysis of a Δ*bacA* mutant revealed a significant reduction in both stalk and cell body length during phosphate starvation (**Figs 9** and **S10**). Notably, deletion of the endopeptidase gene *ldpA*, which lies in a putative operon with *bacA*, had a very similar effect on stalk length, whereas it barely affected the cell body (**Fig 6**). These results suggest that LdpA and BacA may specifically cooperate in stalk formation, whereas BacA is additionally involved in a distinct pathway involved in cell body elongation.

As another scaffolding protein, MreB was shown to be required for stalk formation in media containing moderate to high levels of phosphate [48, 68]. To clarify the contribution of this protein to stalk biosynthesis under phosphate starvation, we employed strains producing MreB or the adapter protein RodZ under the control of an inducible promoter. When starved for phosphate in the absence of inducer, both mutants showed a drastic reduction in stalk length or occasionally even failed to form stalks at all (**Figs 9** and **S10**). The effects on the cell bodies, by contrast, differed depending on the protein depleted. Cells lacking RodZ showed a length distribution indistinguishable from that of the wild-type strain. Depletion of MreB, by contrast, markedly decreased the median cell length. Similar effects were observed for wild-type cells treated with the MreB inhibitor A22 [69, 70] (**Fig 9**). These findings indicate that, under phosphate-limiting conditions, RodZ appears to be specifically required for stalk biosynthesis, whereas MreB additionally contributes to cell body elongation, again supporting the idea that these two processes are driven by distinct mechanisms.

Apart from MreB, the MreCD complex has been identified as a factor critical to lateral growth in many rod-shaped bacteria [4]. MreC is thought to serve as a scaffold that interacts with various PG biosynthetic enzymes, including the monofunctional TPase PBP2 [71, 72] and the monofunctional transglycosylase RodA [73]. In *E*. *coli*, it is part of the elongasome complex [74], whereas it was shown to establish an elongasome-independent structure in *Caulobacter* cells [68, 75]. To test for a role of this protein in stalk formation, we analyzed the morphology of a conditional *mreC* mutant grown under phosphate-limiting conditions (**Fig 9**). In the absence of inducer, the cells started to elongate but eventually became amorphous and lyzed. In most cases, stalks were either absent or barely recognizable, indicating that the MreCD complex may be essential for both cell wall integrity and stalk biosynthesis during phosphate starvation. Given the role of MreC in the activation of PBP2 and RodA in *E*. *coli*, the differences in the phenotypes obtained after inactivation of these proteins are unexpected. Either *Caulobacter* MreC has functionally diverged from its *E*. *coli* homolog or its depletion is more complete than the depletion of MreB or the inhibition of PBP2 with mecillinam. Collectively, our results show that the bactofilin homolog BacA and the elongasome components MreB, RodZ and MreC are required for proper stalk biosynthesis in *Caulobacter* cells.

**Fig 10:**
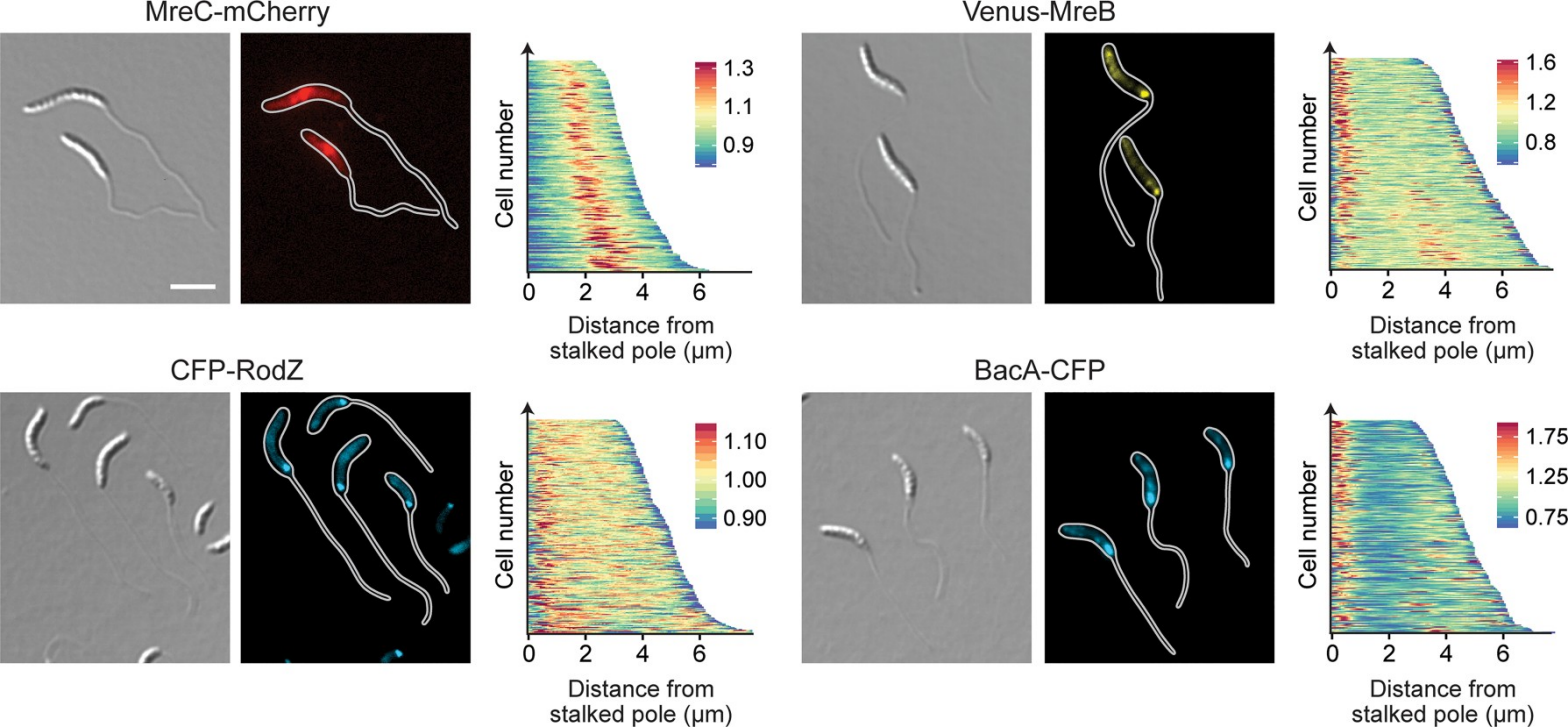
Localization of scaffolding proteins in phosphate-starved cells. Shown is the localization of MreC-mCherry (MAB223, Pxyl::Pxyl*-mreC-mCherry*), CFP-RodZ (CJW2745, *rodZ*::*cfp-rodZ*), Venus-MreB (MT309, Pxyl::Pxyl*-venus-mreB*), and BacA-CFP (MT260, *bacA*::*bacA-cfp*) in cells cultivated for 24 h in M2G^-P^ medium (scale bars: 3 μm). Synthesis of the fluorescent protein fusions was induced with 0.3% xylose 3 h prior to analysis. The population-wide distribution of fluorescence signals was quantified by demographic analysis of random subpopulations of cells (n = 200 for each strain).

To clarify whether the role of the different scaffolding proteins in stalk formation involves their recruitment to the stalked pole, we analyzed the localization patterns of fluorescently tagged derivatives in cells subjected to phosphate starvation (**Fig 10**). Both the MreB and RodZ fusion formed a distinct focus at the stalk base and, in rare cases, also a second focus at the pole opposite the stalk. Together with the polar localization of PBP2 (**Fig 5A**), these findings indicate that key components of the elongasome complex relocate to the site of stalk biosynthesis in phosphate-limiting conditions. There, they colocalize with BacA, which retains its polar position irrespective of changes in the phosphate supply (**Fig 10**). The MreC fusion, by contrast, formed a broad band at midcell, whereas it was largely excluded from the polar regions (**Fig 10**). In line with the global morphological defects caused by its depletion, MreC may have a general role in cell wall biosynthesis, but it does not appear to be stably associated with the polar stalk biosynthetic machinery.

**Fig 11:**
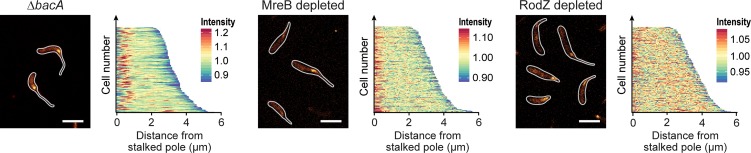
Cell wall biosynthesis in mutants lacking single scaffolding proteins. Shown are fluorescence images of strains LS3809 (Δ*mreB* Pxyl::Pxyl*-mreB*), and CJW2747 (Δ*rodZ*::*Ω* Pxyl::Pxyl*-rodZ* cultivated as described in **Fig 8A** and subjected to HADA staining (2 min). The population-wide distribution of HADA fluorescence was quantified by demographic analysis (n = 200 for each strain) (scale bars: 3 μm).

To determine the role of the different scaffolds in polar PG biosynthesis, cells lacking these factors were subjected to HADA staining after phosphate deprivation (**Fig 11**). Interestingly, despite its severe stalk elongation defect (**Fig 9**) the Δ*bacA* mutant still displayed intense polar foci, indicating that BacA is an accessory factor that is not critical for the global reorganization of PG biosynthesis induced under phosphate-limiting conditions. Consistent with this idea, muropeptide analysis showed that deletion of *bacA* did not have any appreciable effects on global PG composition (**S4 Table**). Depletion of MreB or RodZ, by contrast, strongly decreased the intensity of the polar HADA signals, and frequently led to the insertion of cell wall material at pole-distal sites. In both cases, these defects were accompanied by significant changes in the whole-cell muropeptide profiles. Similar to the Δ*sdpAB* mutant (compare **S3 Table**), the degree of crosslinkage was significantly reduced, mostly due to a decrease in the proportion of highly crosslinked (trimeric and tetrameric) muropeptide species. Moreover, there was a striking increase in the proportion of muropeptides with tripeptide side chains, indicative of high levels of LD-TPase activity. Thus, cell wall stress caused by reduced levels of PBP2-mediated DD-transpeptidation may trigger a fail-safe mechanism that stabilizes the PG meshwork through the formation of abundant 3–3 crosslinks.

Collectively, these results demonstrate that MreB and its transmembrane adapter RodZ play a central role in the establishment of the polar PG biosynthetic zone that gives rise to the stalk structure (see **Fig 4** for a summary).

### MreB orchestrates the polar stalk biosynthetic complex

**Fig 12:**
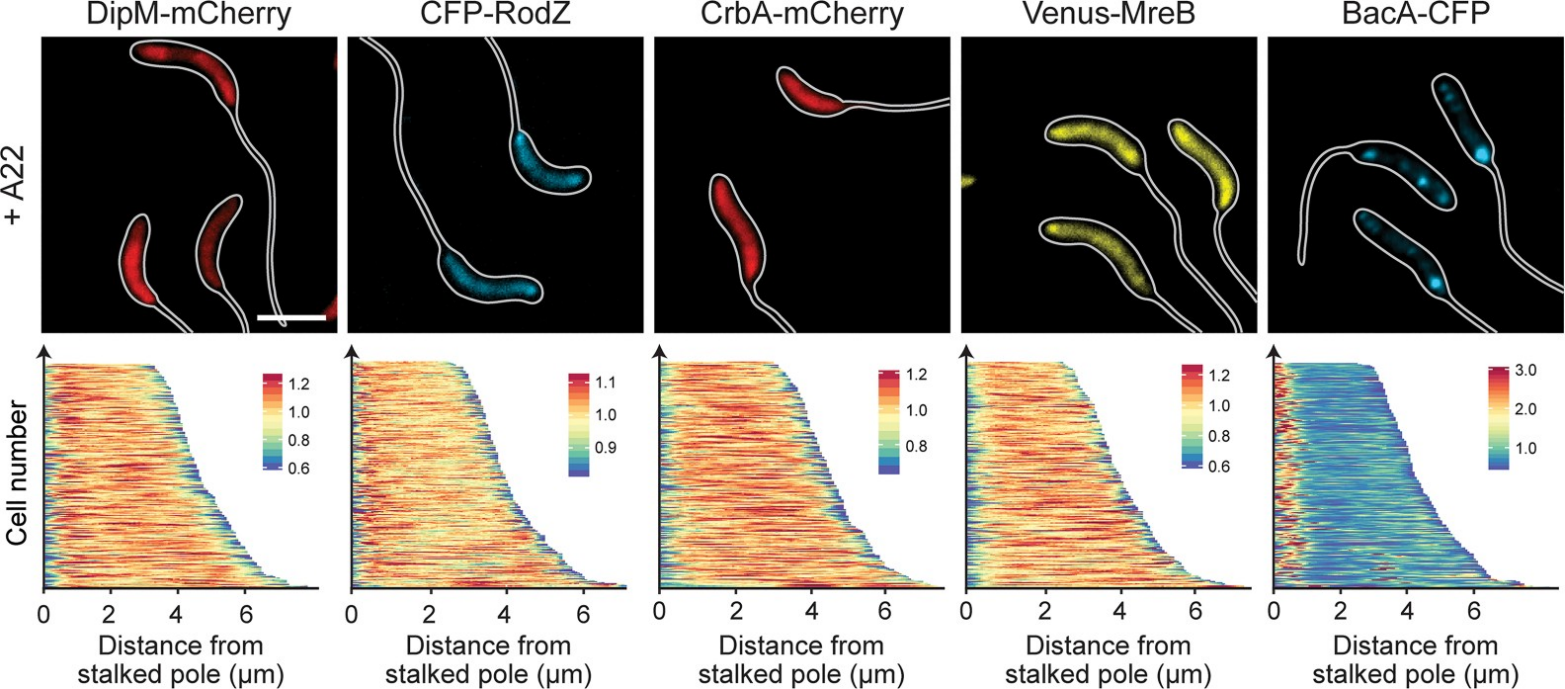
Role of MreB in the polar recruitment of factors involved in stalk formation. Shown are fluorescence images of strains AM208 (Pxyl::Pxyl-*dipM-mCherry*), CJW2745 (*rodZ*::*cfp-rodZ*), MAB247 (Pxyl::Pxyl-*crbA-mCherry*), MT309 (Pxyl::Pxyl*-venus-mreB*) and MT260 (*bacA*::*bacA-cfp*). Cells were grown in PYE medium, diluted into M2G^-P^ medium, and incubated for 23 h. Subsequently, A22 (10 μg/ml) was added to the media, and cultivation was continued for 1 h prior to imaging. Strains AM208, MAB247, and MT309 were induced for 3 h with 0.3% xylose to induce synthesis of the fusion proteins before analysis (scale bars: 3 μm).

Our data demonstrate that several components of the PG biosynthetic machinery localize to the stalked pole in phosphate-starved cells, suggesting that they assemble into a complex mediating the synthesis of stalk PG. To obtain more insight into the factors mediating the recruitment of these proteins, we reanalyzed the localization patterns of DipM-mCherry, CrbA-mCherry, Venus-MreB, CFP-RodZ, and BacA-CFP in all deletion strains that showed defects in stalk elongation (Δ*dipM*, Δ*sdpAB*, Δ*crbA*, Δ*ldpA*, and Δ*bacA*). However, in all cases, the positioning of the fusion proteins remained unaffected, indicating that neither lytic factors nor the bactofilin cytoskeleton are required for complex assembly. Given the prevalence of elongasome components among the polarly localized proteins, we then tested the role of MreB in the recruitment process. Treatment of cells with the MreB inhibitor A22 not only led to the delocalization of the known MreB interactor RodZ but also abolished the polar foci of DipM and CrbA (**Fig 12**). Thus, MreB appears to be a key organizer of the stalk biosynthetic complex. Notably, A22 had no effect on the polar localization of BacA, indicating that the bactofilin scaffold acts independently of MreB.

**Fig 13:**
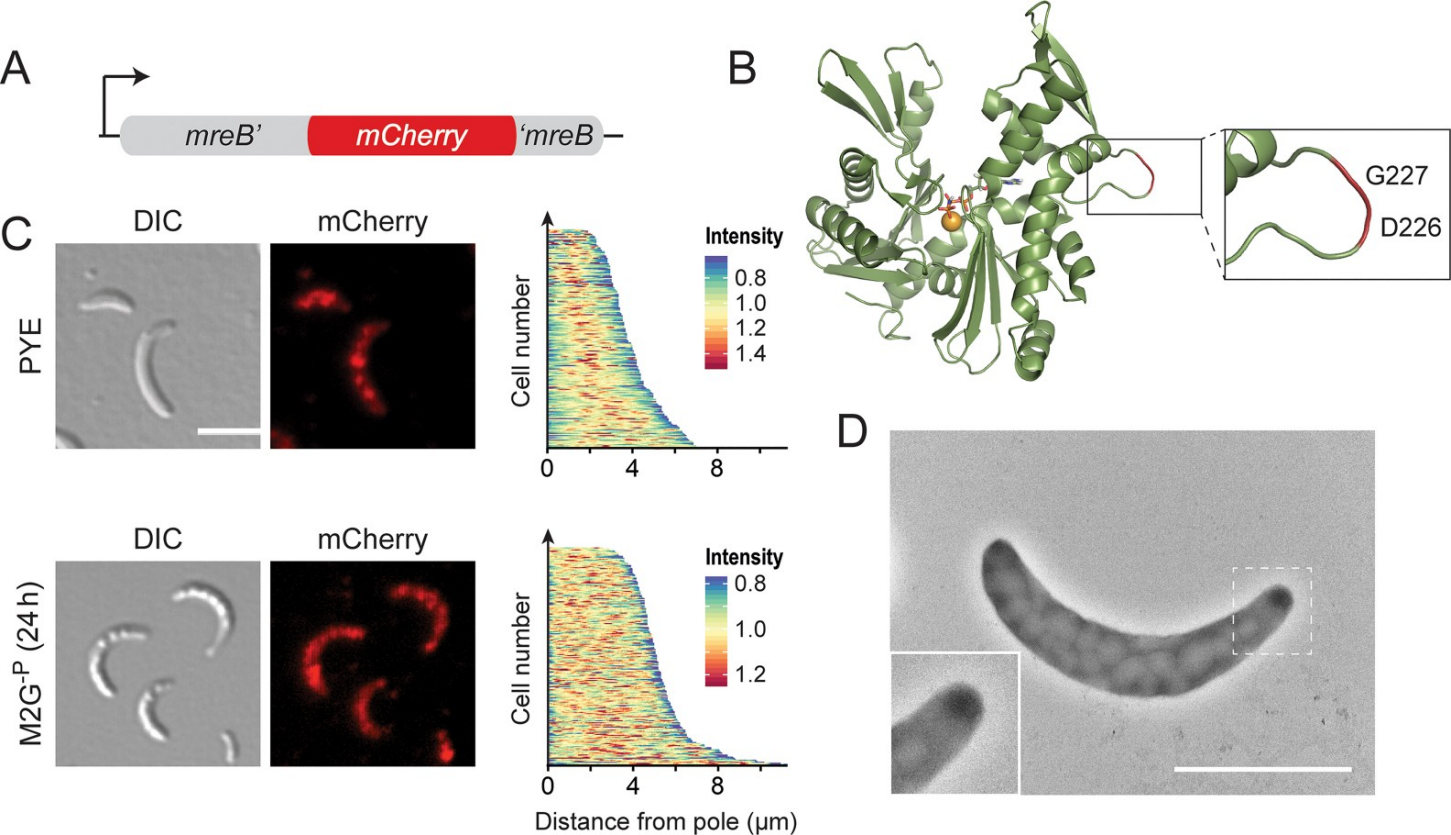
Abolishment of stalk formation in a strain producing an MreB sandwich fusion. (**A**) Schematic representation of the *mreb*^sw^ allele. (**B**) Structure of *Caulobacter* MreB (PDB accession 4CZM; [70]). The inset shows the site used to insert the mCherry tag. (**C**) DIC and fluorescence images of strain MAB238 (*mreB*::*mreB*^*sw*^) grown to exponential phase in PYE medium or incubated for 24 h in M2G^-P^ medium (scale bars: 3 μm). The demographs on the right display the distribution of mCherry fluorescence in random subpopulations of cells (n = 210). (**D**) Transmission electron micrograph of MAB238 cells after 24 h of growth in M2G^-P^ medium and staining with uranyl acetate (2%) (scale bar:2 μm).

To analyze the dynamics of the polar MreB assembly, we constructed a sandwich fusion in which mCherry was inserted into a surface-exposed loop of the MreB protein, following the strategy previously used for its *E*. *coli* homolog [26] (**Fig 13A** and **13B**). A strain carrying the respective allele (*mreB*^sw^) in place of the endogenous *mreB* gene showed normal growth rates (**S11A Fig**). However, in rich medium, the sandwich fusion failed to fully relocate to midcell during the early phases of cell division. Moreover, cells were slightly longer and more highly curved than the wild type and occasionally formed branches and/or filaments (**Figs 13C** and **S11B**). Under phosphate-limiting conditions, by contrast, the distribution of cell lengths was similar to that of the wild-type strain (**S11B Fig**). Strikingly, *mreB*^sw^ cells failed to form stalks in both standard and phosphate-limited medium. Consistent with this observation, the fusion protein no longer condensed into polar foci during phosphate starvation but retained the patchy localization pattern typically observed in exponentially growing cells [76] (**Fig 13C and 13D**). This unusual behavior went along with changes in the global muropeptide profile that were qualitatively similar to those observed for MreB- and RodZ-depleted cells but considerably less pronounced (**S4 Table**). Consistent with the slightly aberrant morphology of the mutant cells, this finding suggests that the insertion of mCherry leads to a mild general defect in MreB function. Importantly, however, it appears to additionally block a specific set of interactions that are critical for the polar recruitment of MreB, thereby preventing stalk formation. To our knowledge this is the first report of a *Caulobacter* strain that is completely stalkless under all growth conditions.

Collectively, these results demonstrate that MreB has a key role in the assembly and function of the polar stalk biosynthetic complex in *Caulobacter* (see **Fig 4** for a summary).

## Discussion

Bacterial cells come in a variety of different shapes, but in most cases the mechanisms generating this morphological diversity are poorly understood [77]. This study uses the *Caulobacter* stalk as a readily amenable model system to investigate the molecular principles underlying the development of species-specific morphological traits. Previous work has shown that stalk growth is driven by zonal PG incorporation at the old cell pole [38, 41, 42]. Initially, this process was thought to be mediated by FtsZ and mechanistically similar to pre-septal cell elongation [68, 78, 79]. However, localization studies revealed that FtsZ is not detectable at the stalked pole, neither during normal cell cycle progression [54] nor during phosphate starvation (**Fig 1B**), excluding the divisome as a relevant player in stalk formation. Other reports implicated MreB and RodZ in stalk growth, suggesting that the elongasome could have a dual role in both cell body and stalk elongation [48, 68]. Clarification of this issue is complicated by the fact that the inactivation of factors with a global role in PG biosynthesis leads to pleiotropic morphological defects. Exploiting the fact that phosphate starvation suppresses *Caulobacter* cell division while strongly promoting stalk elongation, we were able to disentangle cell body- and stalk-specific growth processes and specifically identify proteins involved in the synthesis of stalk PG. Our results indicate that stalk biogenesis is driven by a specialized biosynthetic complex whose composition and biosynthetic activities are clearly distinct from those of the generic cell elongation and division machineries.

**Fig 14:**
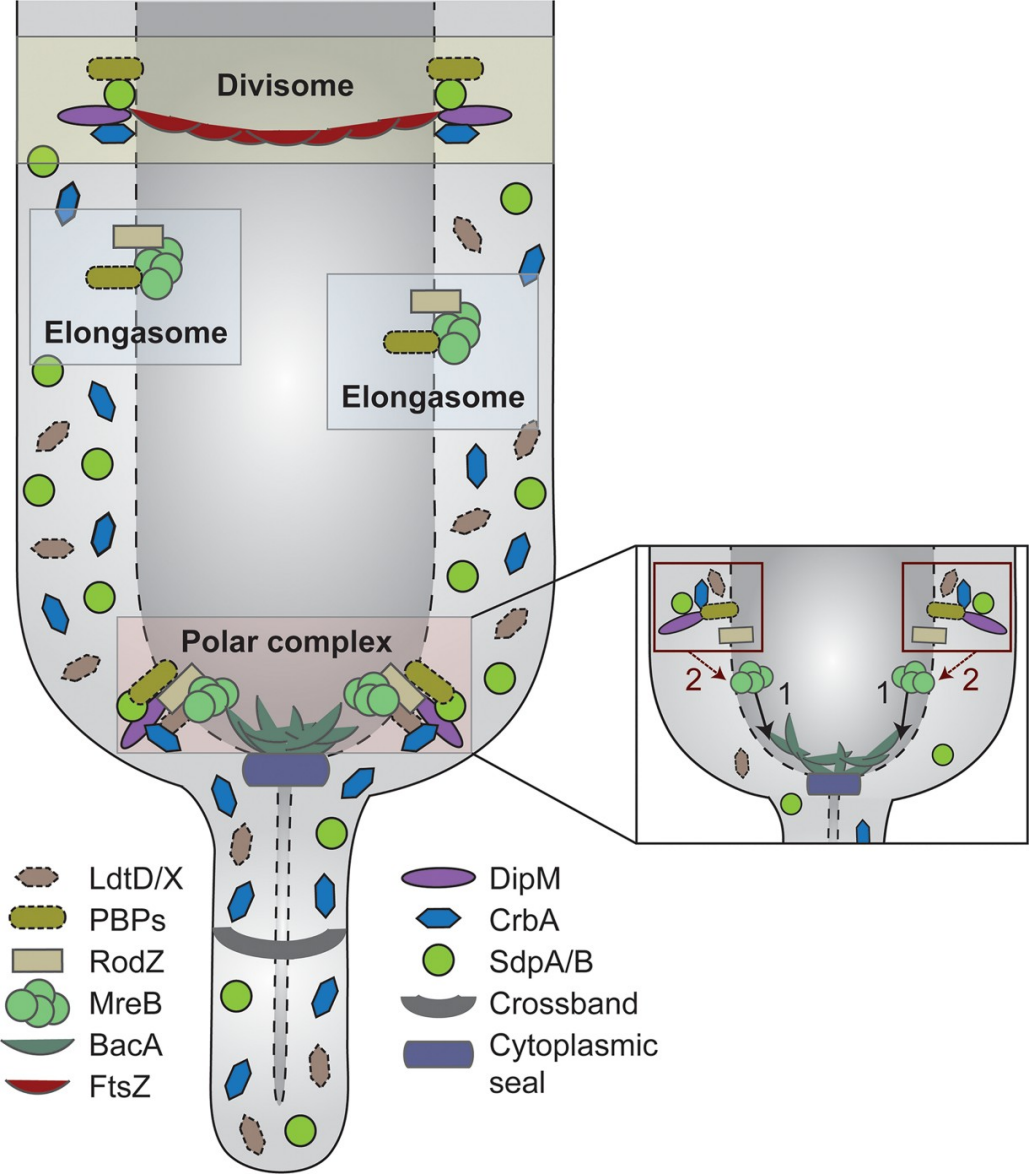
Model of the polar PG biosynthetic complex governing *Caulobacter* stalk formation. The polar complex has a distinct composition and comprises typical elongasome and divisome components. Its basis is formed by the actin homolog MreB, which mediates the recruitment of both synthetic and lytic proteins to the stalked pole (see also inset on the right). The bactofilin BacA contributes to stalk biosynthesis but is not essential to this process. In addition to the proteins that are stably associated with the cell pole, diffusible periplasmic enzymes are transiently associated with the polar machinery or non-selectively trapped in the compartments generated by the crossbands.

Interestingly, the stalk biosynthetic complex is a hybrid composed of factors typically associated with the elongasome (MreB, RodZ, RodA, PBP2) or divisome (DipM, SdpA, SdpB, CrbA) (**Fig 14**). The recruitment of components from the cell elongation machinery may reflect the need to incorporate new cell wall material into an existing sacculus to drive the elongation of the stalk structure, a process that may be mechanistically similar to dispersed PG biosynthesis during lateral cell growth. Notably, HADA (**Fig 2A**) and D-Cysteine [41] labeling clearly indicate that, during stalk growth, newly synthesized PG is not primarily detected in the basal stalk segment but rather in the adjacent polar regions of the cell body. This observation suggests that stalk elongation does not occur simply by addition of new material to the existing stalk template. Instead, it appears to be mediated through expansion of the stalk-proximal polar cap and its simultaneous remodeling into a new stalk segment, a process reminiscent of the medial growth and constriction of the PG sacculus during *Caulobacter* cell division. The common requirement for extensive PG remodeling may explain why the cell division and stalk biosynthetic complexes show a considerable overlap in their autolytic machineries. Interestingly, the importance of some of these shared components varies substantially between the two complexes. For instance, combined inactivation of the lytic transglycosylases SdpA and SdpB has no obvious effect on cell division [64], whereas it largely abolishes stalk formation, indicating that the functional context of these proteins varies depending on the process they mediate. It remains to be clarified to what extent the different cell wall biosynthetic complexes compete for their shared components. Interestingly, during the *Caulobacter* cell cycle, stalk growth occurs predominantly within a short time window at the transition from dispersed to medial peptidoglycan biosynthesis (**S1 Fig**). It is therefore tempting to speculate that the elongasome and divisome have higher priority in the recruitment of shared factors, thereby restricting assembly of the stalk biosynthetic complex to phases in which they are not fully active. Overall, stalk formation clearly demonstrates how the reshuffling of preexisting machinery can serve as a straightforward means to generate novel morphological features in bacteria (see also [80]). The striking diversity of cell shapes observed in certain lineages, such as the alphaproteobacteria, may therefore not be based on major new additions to the repertoire of cell wall biosynthetic proteins but rather on subtle changes in protein activities and localization patterns.

A key finding of our work is the central role of MreB in the stalk biosynthetic complex. We show that this cytoskeletal protein condenses at the stalked pole during phosphate starvation and facilitates the polar recruitment of several other factors that are critical to stalk formation. Notably, our attempts to integrate mCherry into a surface-exposed loop of MreB led to the serendipitous identification of a *Caulobacter* strain that was completely devoid of stalks under both phosphate-limiting and -replete conditions. This is in stark contrast to other mutants described previously, which are stalk-less in rich medium but still elaborate stalks upon phosphate starvation [43–46], suggesting that they have a defect in the regulation of stalk formation rather than in the biosynthetic machinery mediating this process. Importantly, apart from their failure to form stalks, cells producing the MreB sandwich fusion showed only mild general cell shape defects. The region surrounding the insertion site of mCherry may thus contain determinants that are specifically required for MreB’s function in stalk formation but largely dispensable for elongasome-mediated longitudinal growth of the cell body. Previous work has shown that the positioning of MreB filaments is strongly influenced by their intrinsic curvature [81, 82], a parameter controlled by the concentration of the membrane adapter RodZ [83]. The high enrichment of the MreB-RodZ complex at the stalked pole may thus be sufficient to change the architecture of MreB filaments such as to facilitate their interaction with the more highly curved stalked pole. However, the cues promoting the relocation of MreB from the lateral regions of the cell to the stalked pole still remain unknown.

Although MreB clearly has a key role in stalk biogenesis, it is not the only scaffolding protein contributing to this process. Previous work has shown that the bactofilin BacA is required for proper stalk length [50], and our analyses revealed an additional role for this protein in cell body elongation during phosphate starvation (**Fig 9**). Notably, the *bacA* gene lies in a putative operon with *ldpA*, a gene encoding a putative LytM-like endopeptidase that also functions in stalk formation. This genetic context is conserved in a variety of other species, suggesting a functional link between the two gene products [84, 85]. Support for this notion comes from studies in the human pathogen *Helicobacter pylori*, which demonstrated that both genes in this conserved operon are required to establish the characteristic helical cell shape of this species [84]. Notably, apart from its putative interaction with LdpA, *Caulobacter* BacA was shown to recruit a class A PBP (PbpC) involved in stalk elongation and in the targeting of proteins to the stalk lumen [50, 86]. Importantly, the polar localization of BacA was independent of the presence of MreB. The bactofilin cytoskeleton thus appears to constitute a functionally independent morphogenetic module that has been coopted by *Caulobacter* to modulate stalk formation. This module appears to act downstream of the MreB-dependent stalk biosynthetic complex, as it was not able to establish a stalk structure in the absence of a functional MreB cytoskeleton.

The ultimate determinant mediating the polar recruitment of the stalk biosynthetic machinery in *Caulobacter* still remains unknown. In *Asticcacaulis excentricus*, a member of the *Caulobacteraceae* that is characterized by subpolar stalks, the site of stalk formation was shown to be defined by the polarity determinant SpmX [87]. However, despite its conservation, this protein is not required for proper stalk localization in *Caulobacter* cells [88]. Notably, deletion of SpmX or transfer of the cells to phosphate-limited media restores polar stalk growth in *A*. *excentricus* [87], suggesting that the pathway observed in *Caulobacter* is still present in *A*. *excentricus* but normally obscured by the the action of the newly coopted localization factor SpmX. It will be interesting to see whether the *A*. *excentricus* SpmX homolog organizes an alternative stalk biosynthetic complex or simply recruits the polar machinery to a pole-distal position.

Although the functionality and localization of the peptidoglycan biosynthetic machinery changes drastically upon transition of *Caulobacter* cells from phosphate-replete to phosphate-limiting media, the overall composition of their PG layer remains largely unaffected. This finding is unexpected because significant changes in both glycan chain lengths and the degree of cross-linking were observed in other species in response to changes in their growth conditions [89]. However, analyzing the muropeptide profiles of isolated stalk and cell body fractions, we identified clear differences between these two compartments that are likely obscured in whole-cell analyses due to the small contribution of stalks to the total cellular PG content. Most importantly, stalk PG showed a significantly higher degree of crosslinkage, which was mostly due to a higher frequency of 3–3 crosslinks, indicative of elevated LD-TPase activity. The precise reason for this difference remains unclear. It is conceivable that the LD-TPases LdtD and LdtX are part of the polar stalk biosynthetic complex and, thus, preferentially act on newly synthesized PG produced by this machinery. However, localization studies did not give any evidence for an enrichment of these proteins at the stalked pole. An alternative explanation may be provided by the observation that the turnover rate of PG is significantly lower in the stalk than in the cell body. Thus, LD-TPases may act uniformly throughout the entire cell envelope, but most of the 3–3 crosslinks formed in the cell body may be lost as a consequence of PG remodeling, whereas those in the stalk are retained over prolonged periods of time. Notably, peptides with 3–3 crosslinks are stiffer than those with 3–4 crosslinks and adopt a more extended conformation that is better suited to connect glycan strands in stressed PG [90]. Their increased frequency may therefore help to modulate the mechanical properties of the stalk and render it more resistant to bending or breakage under conditions of high laminar flow [37, 91]

Collectively, our study shows that, in *Caulobacter*, multiple cell-wall biosynthetic machineries act in concert to generate stalks of proper size and stability, thereby ensuring optimal performance of this cellular structure in the environmental context. It will be interesting to see how the nature and the regulation of these components have changed during evolution to bring about the large variety of morphologies found in other stalked members of the alphaproteobacterial lineage.

## Materials and methods

### Media and growth conditions

*Caulobacter* strains [92] were grown at 28°C in peptone-yeast-extract (PYE) medium [35], supplemented with antibiotics at the following concentration when appropriate (μg ml^-1^; liquid/solid medium): spectinomycin (25/50), streptomycin (-/5), gentamicin (0.5/5), kanamycin (5/25), chloramphenicol (1/1), mecillinam (15/-). Gene expression from the *xylX* promoter (Pxyl) or *vanA* promoter (Pvan), was induced by supplementation of the media with 0.3% D-xylose and 0.5 mM sodium vanillate, respectively, prior to analysis of the cells. To induce phosphate starvation, stationary cells were diluted 1:20 in M2G^-P^ medium [50] and incubated at 28°C for the indicated times. In case of the conditional *mreB*, *rodZ*, and *mreC* mutants, cells were grown to exponential phase (OD_600_ ~ 0.5) in PYE medium supplemented with xylose, washed three times, and then resuspended to an OD_600_ of 0.05 in inducer-free medium. The cultures were then grown for 7 h to achieve protein depletion, diluted (1:20) in M2G^-P^ medium, and cultivated for additional 24 h before analysis. The conditional *amiC* and *dipM* mutants were treated in a similar fashion, with 12 h of cultivation in PYE medium prior to transfer into M2G^-P^. The synchronization of *Caulobacter* was achieved by density gradient centrifugation using Percoll (Sigma-Aldrich) [93]. To determine the viable-cell count in cultures, various dilutions of the cell suspensions were spread on PYE plates, and the number of colony-forming units (CFU) was determined after three days of incubation at 28°C. *E*. *coli* strain TOP10 (Invitrogen) and its derivatives were cultivated at 37°C in LB broth (Karl Roth, Germany). Antibiotics were added at the following concentrations (μg/ml; liquid/solid medium): spectinomycin (50/100), gentamicin (15/20), kanamycin (30/50), chloramphenicol (20/30).

### Plasmid and strain construction

The bacterial strains, plasmids, and oligonucleotides used in this study are listed in **S5–S8 Tables**. *E*. *coli* TOP10 (Invitrogen) was used as host for cloning purposes. All plasmids were verified by DNA sequencing. *Caulobacter* was transformed by electroporation. Non-replicating plasmids were integrated into the *Caulobacter* chromosome by single-homologous recombination at the *xylX* (Pxyl) or *vanA* (Pvan) locus [94]. Gene replacement was achieved by double-homologous recombination using the counter-selectable *sacB* marker (M.R.K. Alley, unpublished) [54]. Proper chromosomal integration or gene replacement was verified by colony PCR.

### Growth curves

Cells were grown to exponential phase in PYE medium, harvested by centrifugation, and resuspended in the same medium to an OD_600_ of 0.05. The suspensions were then transferred to 24‐well polystyrene microtiter plates (Becton Dickinson Labware), incubated at 32°C with double‐orbital shaking in an Epoch 2 microplate reader (BioTek, Germany), and analyzed photometrically (OD_600_) at 15 min intervals.

### Light and immunofluorescence microscopy

For light microscopic analysis, cells were transferred onto pads made of 1% agarose. Images were taken with an Axio Observer.Z1 (Zeiss, Germany) microscope equipped with a Plan Apochromat 100x/1.45 Oil DIC and a Plan Apochromat 100x/1.4 Oil Ph3 phase contrast objective, an ET-mCherry filter set (Chroma, USA), and a pco.edge sCMOS camera (PCO, Germany). Images were recorded with VisiView 3.3.0.6 (Visitron Systems, Germany) and processed with Metamorph 7.7.5 (Universal Imaging Group, USA) and Illustrator CS6 (Adobe Systems, USA). To generate demographs, fluorescence intensity profiles were measured with ImageJ 1.47v (http://imagej.nih.gov/ij). The data were then processed in R version 3.5.0 [95] using the Cell Profiles script (http://github.com/ta-cameron/Cell-Profiles) [96]. Box and violin plots for the statistical analysis of imaging data were generated in R version 3.5.0 using the ggplot2 [97] and Reshape2 [98] packages, respectively.

### Electron microscopy

10 μl cell suspension were applied to an electron microscopy grid (Formvar/Carbon Film on 300 Mesh Copper; Plano GmbH, Germany) and incubated for 1 min at room temperature. Excess liquid was removed with Whatman filter paper. Subsequently, the cells were negatively stained for 5 sec with 5 μl of 1% uracyl acetate. After three washes with H_2_O, the grids were dried, stored in an appropriate grid holder, and analyzed in a 100 kV JEM-1400 Plus transmission electron microscope (JEOL, USA).

### Western blot analysis

Western blot analysis was performed as described [54], using anti-CtrA [99], anti-FtsZ [63], anti-MipZ [54], anti-DnaA [100], or anti-SpmX [88] at dilutions of 1:10,000 (anti-CtrA, anti-FtsZ, anti-MipZ, and anti-DnaA), and 1:50,000 (anti-SpmX). Goat anti-rabbit immunoglobulin G conjugated to horseradish peroxidase (Perkin Elmer, USA) was used as secondary antibody. Immunocomplexes were detected using the Western Lightning Plus-ECL chemiluminescence reagent (Perkin Elmer, USA). Signals were recorded with a ChemiDoc MP imaging system (Bio-Rad) and analyzed using the Image Lab 5.0 software (Bio-Rad).

### HADA staining

HADA-staining experiments were conducted as described [42]. Briefly, 50 μl of a culture were incubated for 2 min with 0.5 mM HADA. The cells were then fixed by addition of ice-cold ethanol to a concentration of 70% and incubated at 4°C for 20 min. Subsequently, they were washed three times with PBS and subjected to fluorescence microscopic analysis. For chase experiments, phosphate-starved *Caulobacter* cells were grown for 90 min in the presence of 0.5 mM HADA. The cells were washed three times with M2G^-P^ medium, resuspended in fresh M2G^-P^ medium, and further cultivated for the indicated time intervals. Cells were fixed and washed as described above prior to imaging.

### Bioinformatic analysis

Protein sequences containing the indicated domains were retrieved from the UniProt Knowledgebase [101]. Their overall domain composition was determined using the SMART server [102]. The prediction of protein localization and membrane topology was performed with Signal-BLAST [103] and TMHMM [104], respectively.

### Flow cytometry

Cultures were grown in the indicated media and supplemented with 20 μg/ml rifampicin 3 h prior to analysis to block the re-initiation of chromosome replication. At the indicated time points, cells were diluted to an OD_600_ of 0.1–0.2, incubated for 25 min under vigorous shaking with the DNA-specific fluorescent dye Hoechst 33342 (10 μM; Thermo Fisher Scientific, Germany), and fixed by addition of ethanol to a final concentration of 70%. Subsequently, the suspensions were analyzed by flow cytometry in a customized Fortessa Flow Cytometer (BD Biosciences, Germany), using the UV 440/40 nm channel. Data were acquired with FACSdiva 8.0 (BD Biosciences) and processed with FlowJo v10 (FlowJo LLC, USA).

### Peptidoglycan analysis

For whole-cell analyses, cultures were rapidly cooled to 4°C and harvested by centrifugation at 16,000 rpm for 30 min. The cells were resuspended in 6 ml of ice-cold H_2_O and added dropwise to 6 ml of a boiling solution of 8% sodium dodecylsulfate (SDS) that was stirred vigorously. After 30 min of boiling, the suspension was cooled to room temperature. Peptidoglycan was isolated from the cell lysates as described previously [105] and digested with the muramidase cellosyl (kindly provided by Hoechst, Frankfurt, Germany). The resulting muropeptides were reduced with sodium borohydride and separated by HPLC following an established protocol [105, 106]. The identity of eluted fragments was assigned based on the retention times of known muropeptides from *Caulobacter* [107].

To prepare stalk and cell body fractions, 100 ml cultures grown in M2G^-P^ medium were rapidly cooled to 4°C and harvested by centrifugation at 16,000 rpm for 30 min. After resuspension in M2G^-P^ medium, the cells were vigorously agitated for 2 min at maximum speed in a kitchen blender. The suspension was submitted to three rounds of centrifugation at 9,000 rpm and 4°C. The supernatants (stalk fraction) and the first pellet (cell body fraction) were collected separately and kept in ice. The stalk fraction was subjected to an additional centrifugation step at 10,000 rpm and 4°C to remove residual cell bodies and cell debris. Subsequently, stalks were collected by centrifugation at 20,000 rpm and 4°C for 30 min, resuspended in 3 ml ice-cold H_2_O, added dropwise to 3 ml of a boiling 8% SDS solution, and then further processed as described above to isolate stalk PG. The isolation of cell body PG was achieved as described for whole-cell samples.

## Supporting information

S1 Fig Growth pattern of *Caulobacter* cells in phosphate-replete conditions. (**A**) Schematic representation of the *Caulobacer* cell cycle. (**B**) HADA incorporation in synchronized wild-type cells growing in rich medium. Wild-type (NA1000) swarmer cells were transferred into PYE medium and cultivated for the duration of one cell cycle. At the indicated time points, samples were taken, pulse-labeled (2 min) with HADA, and subjected to fluorescence microscopy (scale bar: 3 μm). The demographs show the distribution of HADA fluorescence in random subpopulations of cells (n = 200 per time point).
(TIF)

S2 Fig Changes in HADA incorporation during transition to phosphate starvation. (**A**) Distribution of newly synthesized PG after different times of phosphate starvation. Cells of wild-type strain NA1000 were cultivated in M2G^-P^ medium for the indicated amount of time and exposed to a short (2 min) pulse of HADA. After microscopic analysis, the distribution of fluorescence along the long axis of the cells was determined by line scan analysis for multiple cells per time point. The curves obtained were normalized to the average cell length of the population analyzed, aligned at the center of the stalked-pole focus and averaged (n = 42 at 8 h, n = 40 at 18 h, and n = 44 at 40 h). (**B**) Intensity of HADA fluorescence at the stalked pole in wild-type (NA1000) cells cultivated in M2G^-P^ medium for 8 h (n = 51), 18 h (n = 60), 28 h (n = 54), and 40 h (n = 54). Error bars represent standard deviations. (**C**) Slow turnover of PG in the stalk. Cells were cultivated in M2G^-P^ medium for 18 h and exposed to HADA for an extended period of time (1.5 h) to uniformly label their peptidoglycan layer. Subsequently, they were washed, transferred into HADA-free M2G^-P^ medium, and cultivated for 2 h, 4 h, and 6 h in the absence of the label (scale bars: 3 μm). To quantify the changes in HADA fluorescence overtime, fluorescence profiles were obtained from random subpopulations of cells (n = 200 per time point). The lengths of the profiles in each quintile of the cell length distribution were normalized to the maximum cell length in the respective quintile, and the fluorescence intensities were averaged and shown as violin plots.
(TIF)

S3 Fig Microscopic analysis of the stalk and cell body fractions. Cells were cultivated for 24 h in M2G^-P^ medium, agitated vigorously, and then subjected to differential centrifugation to separate stalks and cell bodies. Samples of the intact cells and the stalk and cell body fractions were visualized by phase contrast microscopy (scale bar: 3 μm).
(TIF)

S4 Fig Role of PBP2 and RodA in stalk elongation. (**A**) DIC micrographs of cells deficient in PBP2 or RodA activity. Strain NA1000 (wild type) was diluted into M2G^-P^ medium containing mecillinam (+) and cultivated for 24 h prior to analysis. Cells of strain MAB407 (Δ*rodA* Pxyl::Pxyl*-rodA*) were first grown for 8 h in PYE medium lacking xylose to deplete RodA, diluted 1:20 into xylose-free M2G^-P^ medium, and then cultivated for another 24 h. As controls, cells were grown in the absence of mecillinam (-) or in the presence of the inducer xylose, respectively. (**B**) Distribution of the cell body and stalk lengths in the cultures described in (A). The data are shown as box plots, with the horizontal line indicating the median, the box the interquartile range and the wiskers the 2^nd^ and the 98^th^ percentile (n = 202 per strain). In addition rotated kernel density plots (grey) are depicted for each dataset to indicate the distribution of the raw data (*** p < 10^−6^; t-test). See **S1 File** for the raw data.
(TIF)

S5 Fig Role of class-A PBPs and LD-TPases in stalk elongation. (**A**) Lysis of a conditional mutant lacking all class A PBPs during cultivation in phosphate-limiting conditions. Cells of strain WS056 (Δ*pbpY* Δ*pbp1A* Δ*pbpC* Δ*pbpZ* Pxyl::Pxyl*-pbpX*) were pre-grown until exponential phase in PYE medium containing the inducer xylose, transferred in PYE without xylose after two washing steps, and grown for additional 12 h until they reached stationary phase. Then cells were diluted (1:20) into xylose-free M2G^-P^ medium, and cultivated for 24 h prior to visualization by DIC microscopy (scale bar: 3 μm). (**B**) Localization of fluorescently tagged class A PBPs under conditions of phosphate starvation. Cells of strains AM457 (Pxyl::Pxyl-*venus-pbpY*), KK33 (Pxyl::Pxyl-*venus-pbp1a*), MT279 (Pxyl::Pxyl-*venus-pbpC*), AM458 (Pxyl::Pxyl-*venus-pbpZ*), and MT278 (Pxyl::Pxyl-*venus-pbpX*), were grown for 24 h in M2G^-P^ medium and visualized by fluorescence microscopy. Three hours prior to analysis, the cultures were supplemented with 0.3% xylose to induce synthesis of the fusion proteins (scale bars: 3 μm). (**C**) Localization of fluorescently tagged LD-TPases under conditions of phosphate starvation. Shown are cells of strains MAB389 (Pxyl::Pxyl-*ldtD-mCherry*) and MAB390 (Pxyl::Pxyl-*ldtX-mCherry)* cultivated and induced as described for panel B (scale bar: 3 μm). Please note that due to the short induction time and the presence of crossbands, the signal is limited to the cell body and the first stalk segment.
(TIF)

S6 Fig Role of autolytic enzymes in stalk elongation. (**A**) Distribution of stalk lengths in populations of mutants lacking predicted autolytic enzymes. Shown are cells of strains AZ52 (Δ*ldpABCDEF)*, AM364 (Δ*ldpA*), MAB386 (Δ*amiC* Pxyl::Pxyl-*amiC*), MAB239 (Δ*CC3740*), MAB233 (Δ*CC3322*), MAB251 (Δ*CC3322* Δ*CC3740*), AZ85 (Δ*CC2775*), MAB248 (Δ*CC2936*), and MAB250 (Δ*CC2936* Δ*CC2775* Δ*CC0349*) harvested after 24 h of cultivation in M2G^-P^ medium. The values obtained (n = 215 per strain) are shown as box plots, with the thick line indicating the median, the box the interquartile range and the wiskers the 2^nd^ and the 98^th^ percentile. In addition rotated kernel density plots (grey) are depicted for each dataset to indicate the distribution of the raw data (*** p < 10^−6^; t-test). See **S1 File** for the raw data.
(TIF)

S7 Fig Phenotypes of autolysin-deficient cells grown in rich medium. Cells of strains NA1000 (WT), MAB360 (Δ*dipM* Pxyl::Pxyl*-dipM*), AM364 (Δ*ldpA*), AZ22 (Δ*sdpAB*), and AM376 (Δ*crbA*) were cultivated in PYE medium and visualized by DIC microscopy.
(TIF)

S8 Fig Transmission electron micrographs of phosphate-starved autolysin mutants. Cells of strains NA1000 (WT), MAB360 (Δ*dipM* Pxyl::Pxyl*-dipM*), AM364 (Δ*ldpA*), AZ22 (Δ*sdpAB*), and AM376 (Δ*crbA*) were grown for 24 h in M2G^-P^ medium, stained with uranyl acetate, and visualized by transmission electron microscopy (scale bars: 2 μm).
(TIF)

S9 Fig Stability of fluorescent protein fusions. Cells producing fluorescently tagged derivatives of SdpA (AM480, Pxyl::Pxyl-*sdpA-mCherry*), DipM (AM208, Pxyl::Pxyl-*dipM-mCherry*), CrbA (MAB247, Pxyl::Pxyl-*crbA-mCherry*), LdpA (MAB293, Pxyl::Pxyl-*mCherry-ldpA*), SdpB (AZ127, Pxyl::Pxyl-*torA’-sdpB-mCherry*), and RodA (MAB405, Pxyl::Pxyl-*gfp*-*rodA*) were grown for 24 h in M2G^-P^ medium and subjected to Western blot analysis. Four hours (MAB293), three hours (AM480, MAB247, AM208, MAB405) or two hours (AZ127) prior to analysis, the media were supplemented with 0.3% xylose to induce the synthesis of the fusion proteins (+). Cells grown in the absence of xylose (-) are shown as controls.
(TIF)

S10 Fig Transmission electron micrographs of mutants lacking scaffolding proteins. Shown are cells of strains NA1000 (WT), LS3809 (Δ*mreB* Pxyl::Pxyl*-mreB*), CJW2747 (Δ*rodZ*::*Ω* Pxyl::Pxyl*-rodZ*), and MT257 (Δ*bacA*) that were stained with uranyl acetate and visualized by transmission electron microscopy (scale bars: 2 μm). Strains NA1000 and MT257 (Δ*bacA*) was grown in M2G^-P^ for 24 h prior to imaging. Strains LS3809, and CJW2747 were grown to exponential phase in PYE medium containing the inducer xylose, washed, cultivated for 7 h in inducer-free PYE medium, and then diluted (1:20) into M2G^-P^ medium 24 h prior to imaging.
(TIF)

S11 Fig Growth characteristics of a strain producing an MreB-mCherry sandwich fusion. (**A**) Growth curves of strains NA1000 (WT) and MAB238 (*mreB*^SW^) in PYE medium. (**B**) Distribution of the cell body lengths in populations of strains NA1000 (WT) and MAB238 (*mreB*^SW^) during exponential growth in PYE medium or after cultivation for 18 h, 24 h and 40 h in M2G^-P^ medium. The values obtained (n = 208 per strain) are shown as box plots, with the thick line indicating the median, the box the interquartile range and the wiskers the 2^nd^ and the 98^th^ percentile. In addition rotated kernel density plots (grey) are depicted for each dataset to indicate the distribution of the raw data (*** p < 10^−6^; t-test). See **S1 File** for the raw data.
(TIF)

S1 Table Composition of cell body and stalk peptidoglycan from from crossband- and LD-transpeptidase-deficient cells. The indicated strains were analyzed after growth for 24 h in M2G^-P^. Values are the mean ± variance of two independent experiments.
(PDF)

S2 Table List of peptidoglycan biosynthesis mutants analyzed for stalk defects. (PDF)

S3 Table Composition of peptidoglycan isolated from autolysin-deficient cells. The indicated strains were analyzed after growth for 24 h in M2G^-P^. The values are the mean ± variance of two independent experiments.
(PDF)

S4 Table Composition of peptidoglycan isolated from cells with defects in scaffolding proteins. Strains MT257 (Δ*bacA*), MAB238 (*mreB*::*mreB*^*sw*^), and NA1000 (WT) were grown for 24 h in M2G^-P^ prior to analysis. Strains CJW2747 (Δ*rodZ*::*Ω* Pxyl::Pxyl*-rodZ*) and LS3809 (Δ*mreB* Pxyl::Pxyl*-mreB*) were first cultivated for 7 h in PYE and then grown for 24 h in M2G^-P^. Values are the mean ± variance of two independent experiments.
(PDF)

S5 Table *C*. *crescentus* strains used in this study. (DOCX)

S6 Table General plasmids used in this work. (DOCX)

S7 Table Plasmids generated in this work. (DOCX)

S8 Table Oligonucleotides used in this work. (DOCX)

S1 File Cell body and stalk lengths (raw data). (XLSX)
