# Supplementary figures and images for: A specialized MreB-dependent cell wall biosynthetic complex mediates the formation of stalk-specific peptidoglycan in Caulobacter crescentus
Source: PLoS Genet. 2019 Feb 1;15(2):e1007897. doi: 10.1371/journal.pgen.1007897 (PMC6373972; doi:10.1371/journal.pgen.1007897)

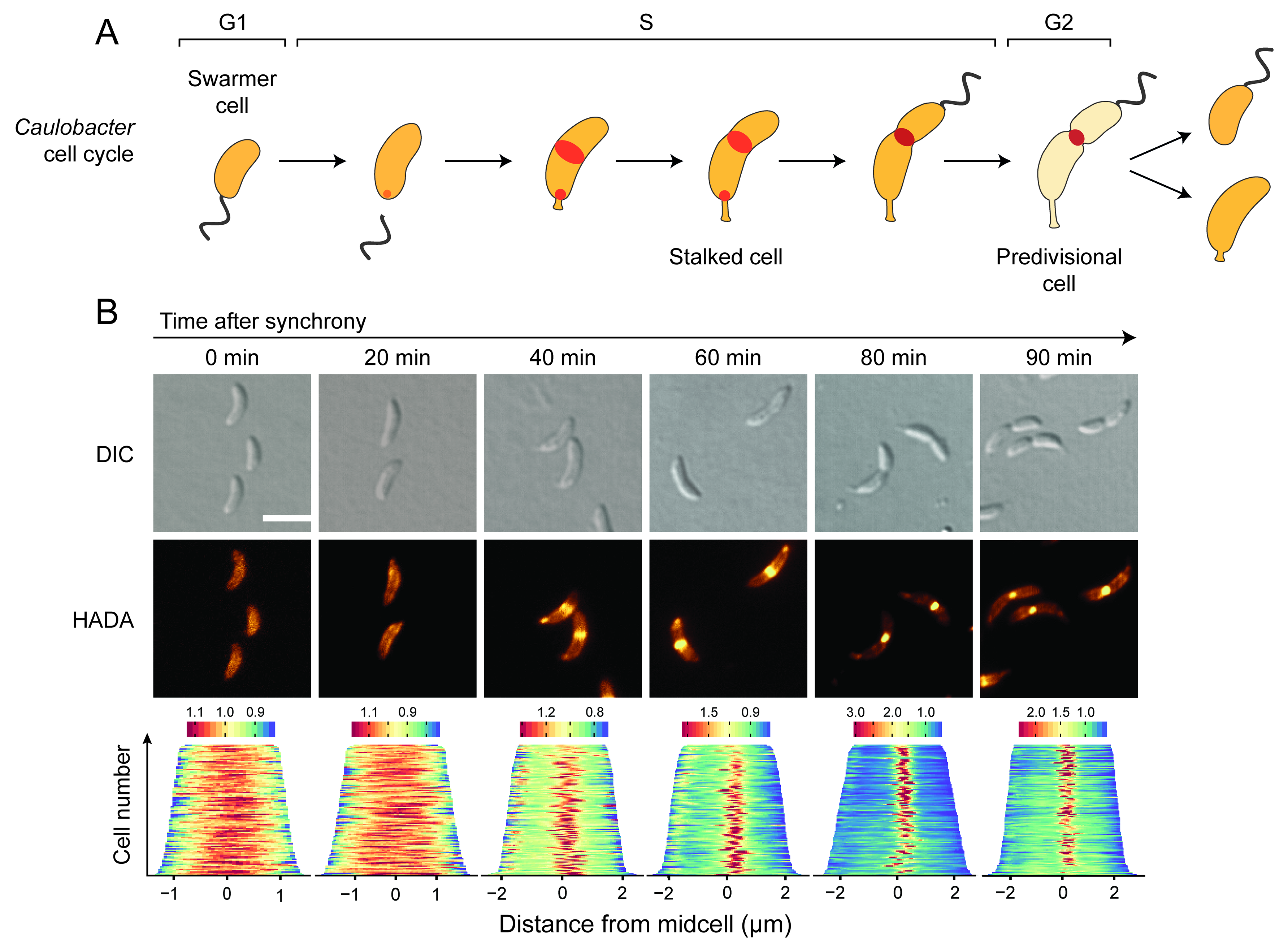

Supplement: S1 Fig — (A) Schematic representation of the Caulobacer cell cycle. (B) HADA incorporation in synchronized wild-type cells growing in rich medium. Wild-type (NA1000) swarmer cells were transferred into PYE medium and cultivated for the duration of one cell cycle. At the indicated time points, samples were taken, pulse-labeled (2 min) with HADA, and subjected to fluorescence microscopy (scale bar: 3 μm). The demographs show the distribution of HADA fluorescence in random subpopulations of cells (n = 200 per time point). (TIF) [file pgen.1007897.s001.tif]

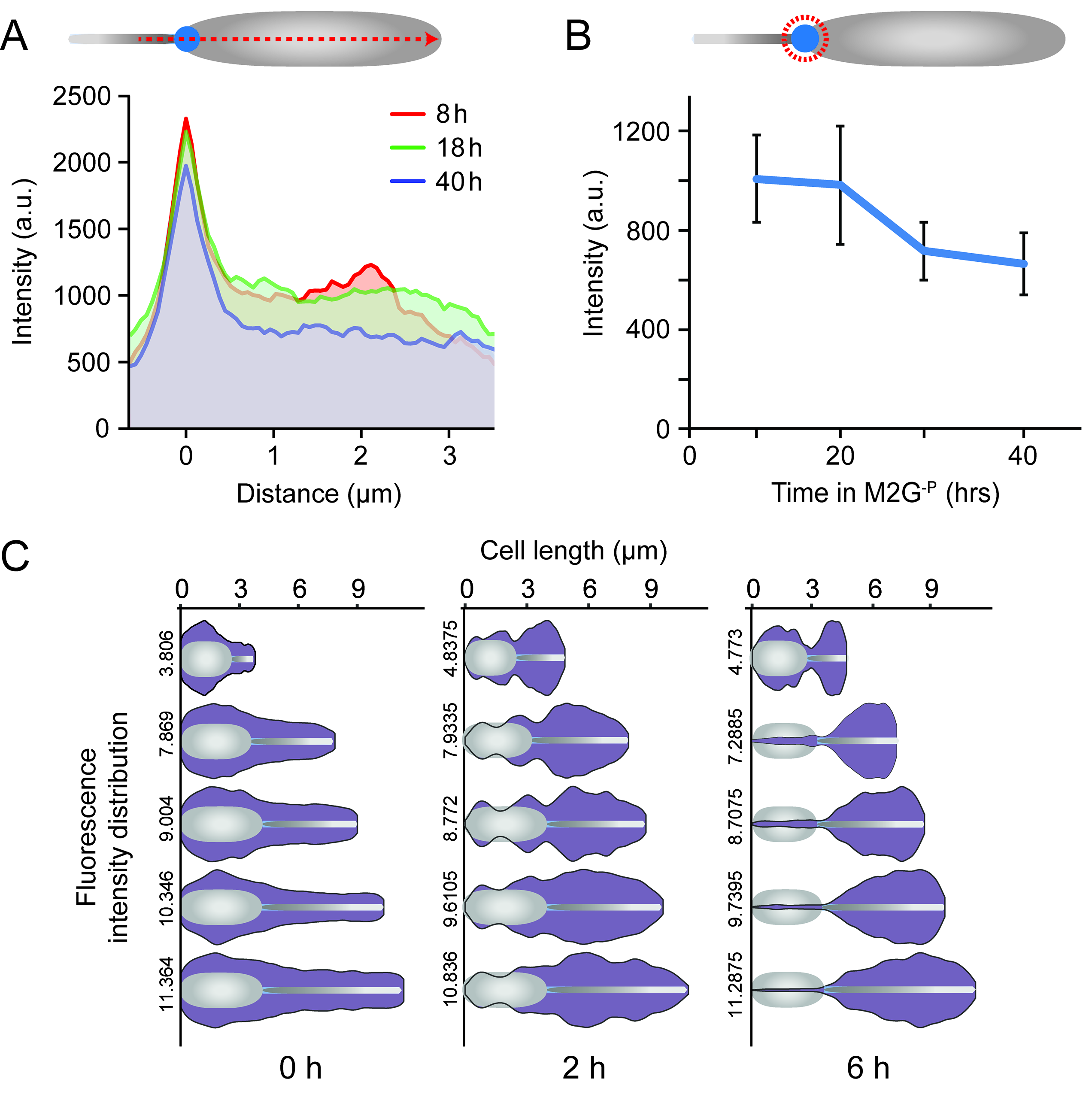

Supplement: S2 Fig — (A) Distribution of newly synthesized PG after different times of phosphate starvation. Cells of wild-type strain NA1000 were cultivated in M2G-P medium for the indicated amount of time and exposed to a short (2 min) pulse of HADA. After microscopic analysis, the distribution of fluorescence along the long axis of the cells was determined by line scan analysis for multiple cells per time point. The curves obtained were normalized to the average cell length of the population analyzed, aligned at the center of the stalked-pole focus and averaged (n = 42 at 8 h, n = 40 at 18 h, and n = 44 at 40 h). (B) Intensity of HADA fluorescence at the stalked pole in wild-type (NA1000) cells cultivated in M2G-P medium for 8 h (n = 51), 18 h (n = 60), 28 h (n = 54), and 40 h (n = 54). Error bars represent standard deviations. (C) Slow turnover of PG in the stalk. Cells were cultivated in M2G-P medium for 18 h and exposed to HADA for an extended period of time (1.5 h) to uniformly label their peptidoglycan layer. Subsequently, they were washed, transferred into HADA-free M2G-P medium, and cultivated for 2 h, 4 h, and 6 h in the absence of the label (scale bars: 3 μm). To quantify the changes in HADA fluorescence overtime, fluorescence profiles were obtained from random subpopulations of cells (n = 200 per time point). The lengths of the profiles in each quintile of the cell length distribution were normalized to the maximum cell length in the respective quintile, and the fluorescence intensities were averaged and shown as violin plots. (TIF) [file pgen.1007897.s002.tif]

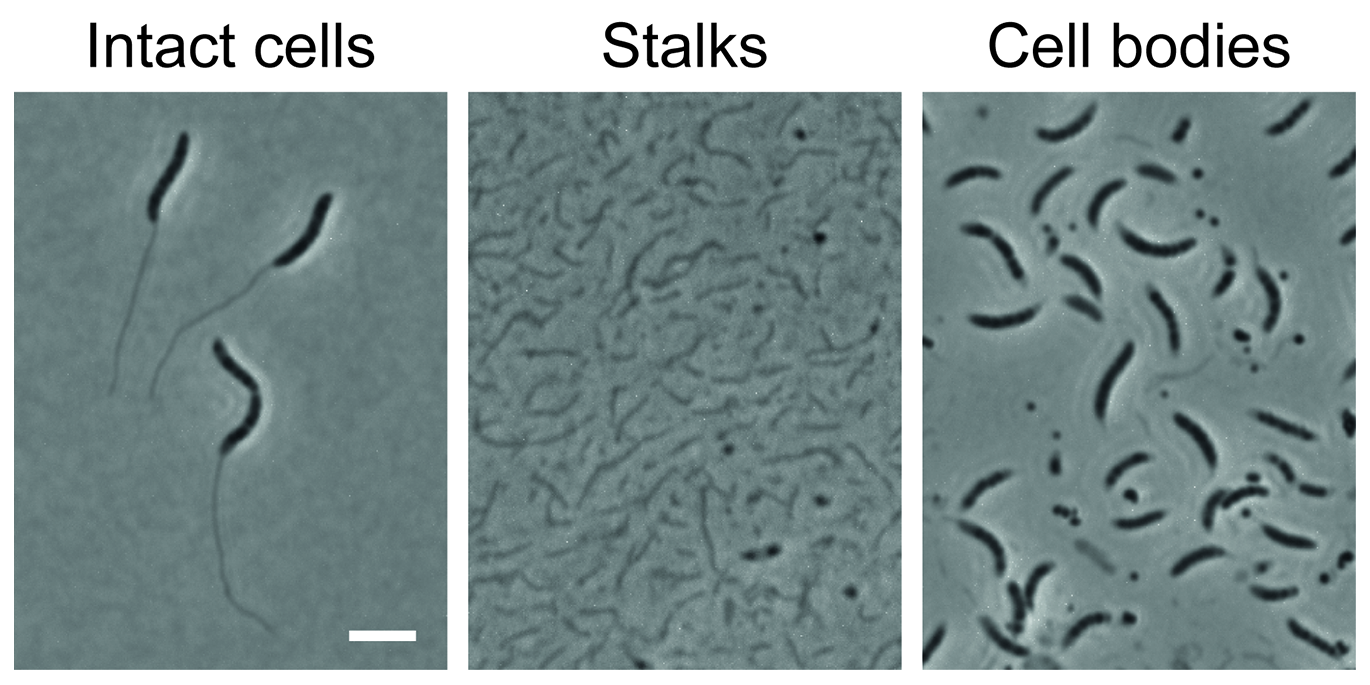

Supplement: S3 Fig — Cells were cultivated for 24 h in M2G-P medium, agitated vigorously, and then subjected to differential centrifugation to separate stalks and cell bodies. Samples of the intact cells and the stalk and cell body fractions were visualized by phase contrast microscopy (scale bar: 3 μm). (TIF) [file pgen.1007897.s003.tif]

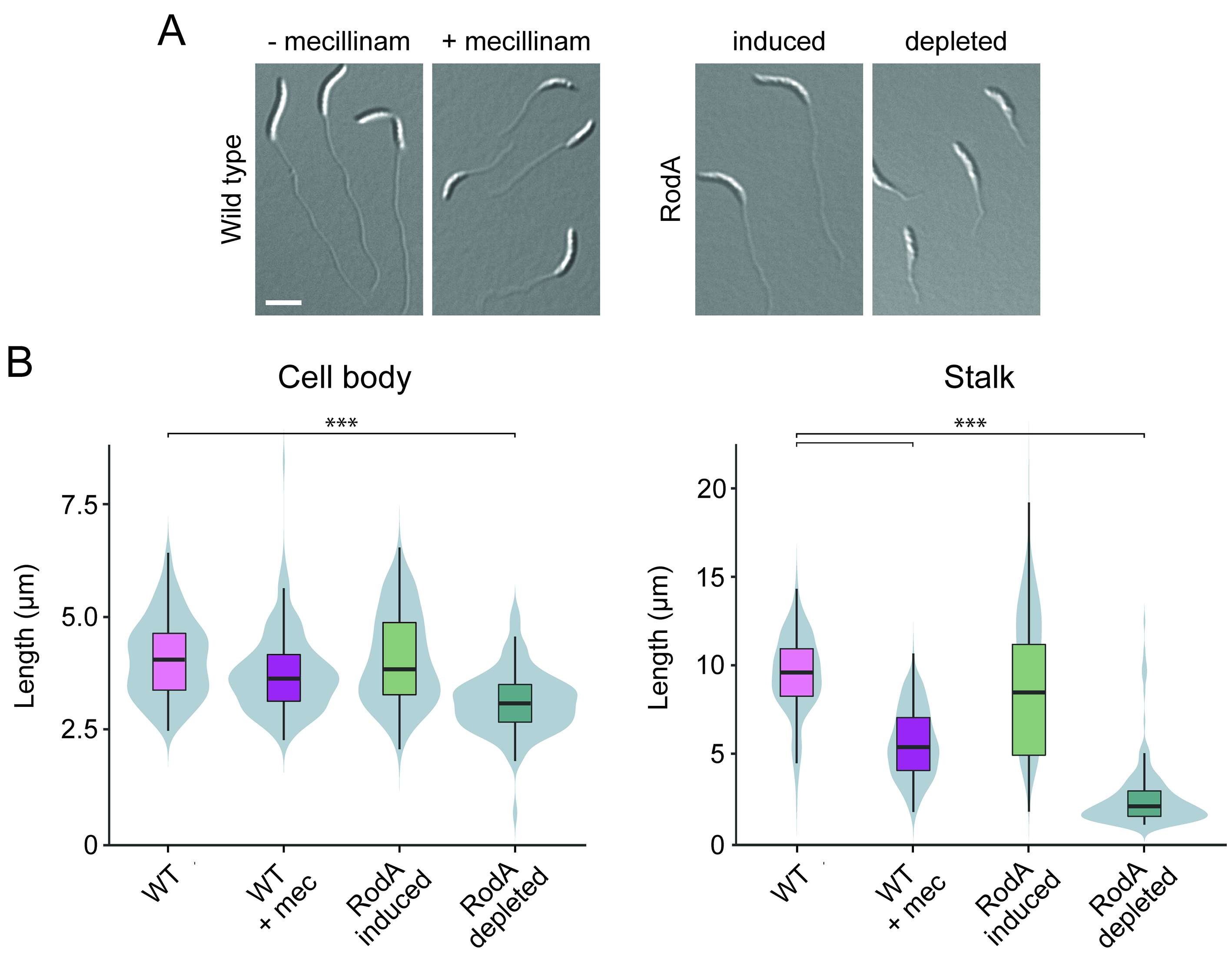

Supplement: S4 Fig — (A) DIC micrographs of cells deficient in PBP2 or RodA activity. Strain NA1000 (wild type) was diluted into M2G-P medium containing mecillinam (+) and cultivated for 24 h prior to analysis. Cells of strain MAB407 (ΔrodA Pxyl::Pxyl-rodA) were first grown for 8 h in PYE medium lacking xylose to deplete RodA, diluted 1:20 into xylose-free M2G-P medium, and then cultivated for another 24 h. As controls, cells were grown in the absence of mecillinam (-) or in the presence of the inducer xylose, respectively. (B) Distribution of the cell body and stalk lengths in the cultures described in (A). The data are shown as box plots, with the horizontal line indicating the median, the box the interquartile range and the wiskers the 2nd and the 98th percentile (n = 202 per strain). In addition rotated kernel density plots (grey) are depicted for each dataset to indicate the distribution of the raw data (*** p < 10−6; t-test). See S1 File for the raw data. (TIF) [file pgen.1007897.s004.tif]

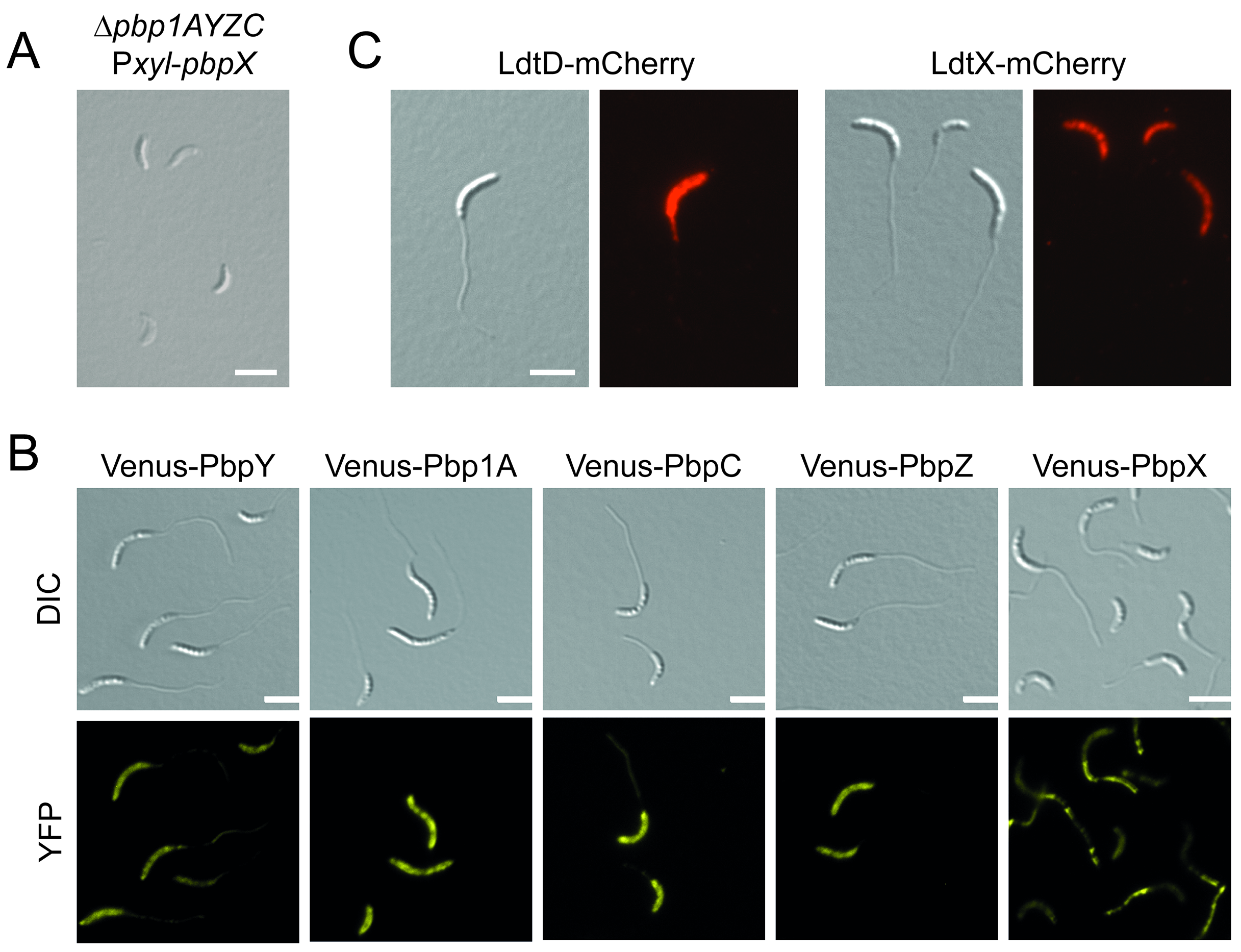

Supplement: S5 Fig — (A) Lysis of a conditional mutant lacking all class A PBPs during cultivation in phosphate-limiting conditions. Cells of strain WS056 (ΔpbpY Δpbp1A ΔpbpC ΔpbpZ Pxyl::Pxyl-pbpX) were pre-grown until exponential phase in PYE medium containing the inducer xylose, transferred in PYE without xylose after two washing steps, and grown for additional 12 h until they reached stationary phase. Then cells were diluted (1:20) into xylose-free M2G-P medium, and cultivated for 24 h prior to visualization by DIC microscopy (scale bar: 3 μm). (B) Localization of fluorescently tagged class A PBPs under conditions of phosphate starvation. Cells of strains AM457 (Pxyl::Pxyl-venus-pbpY), KK33 (Pxyl::Pxyl-venus-pbp1a), MT279 (Pxyl::Pxyl-venus-pbpC), AM458 (Pxyl::Pxyl-venus-pbpZ), and MT278 (Pxyl::Pxyl-venus-pbpX), were grown for 24 h in M2G-P medium and visualized by fluorescence microscopy. Three hours prior to analysis, the cultures were supplemented with 0.3% xylose to induce synthesis of the fusion proteins (scale bars: 3 μm). (C) Localization of fluorescently tagged LD-TPases under conditions of phosphate starvation. Shown are cells of strains MAB389 (Pxyl::Pxyl-ldtD-mCherry) and MAB390 (Pxyl::Pxyl-ldtX-mCherry) cultivated and induced as described for panel B (scale bar: 3 μm). Please note that due to the short induction time and the presence of crossbands, the signal is limited to the cell body and the first stalk segment. (TIF) [file pgen.1007897.s005.tif]

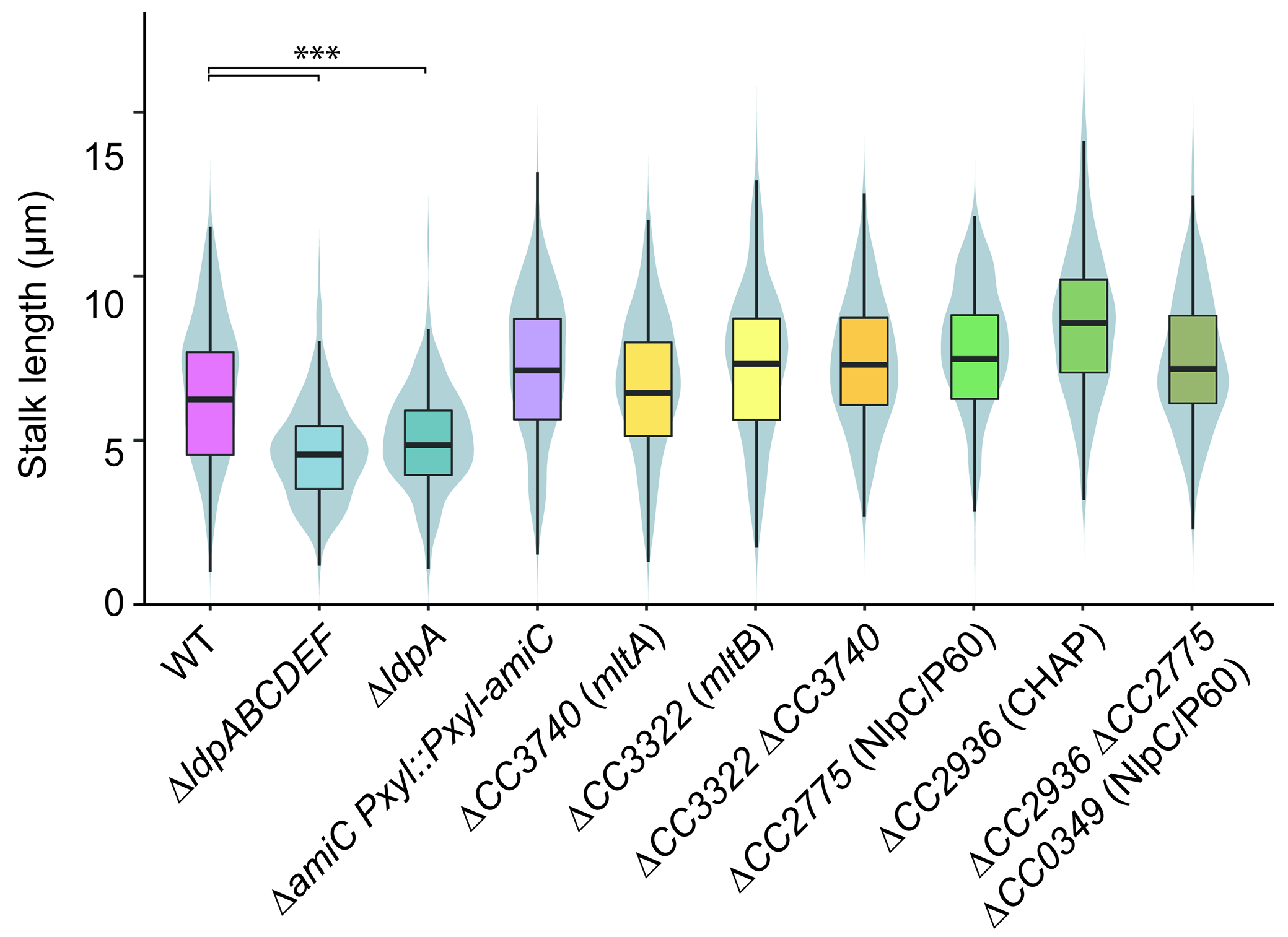

Supplement: S6 Fig — (A) Distribution of stalk lengths in populations of mutants lacking predicted autolytic enzymes. Shown are cells of strains AZ52 (ΔldpABCDEF), AM364 (ΔldpA), MAB386 (ΔamiC Pxyl::Pxyl-amiC), MAB239 (ΔCC3740), MAB233 (ΔCC3322), MAB251 (ΔCC3322 ΔCC3740), AZ85 (ΔCC2775), MAB248 (ΔCC2936), and MAB250 (ΔCC2936 ΔCC2775 ΔCC0349) harvested after 24 h of cultivation in M2G-P medium. The values obtained (n = 215 per strain) are shown as box plots, with the thick line indicating the median, the box the interquartile range and the wiskers the 2nd and the 98th percentile. In addition rotated kernel density plots (grey) are depicted for each dataset to indicate the distribution of the raw data (*** p < 10−6; t-test). See S1 File for the raw data. (TIF) [file pgen.1007897.s006.tif]

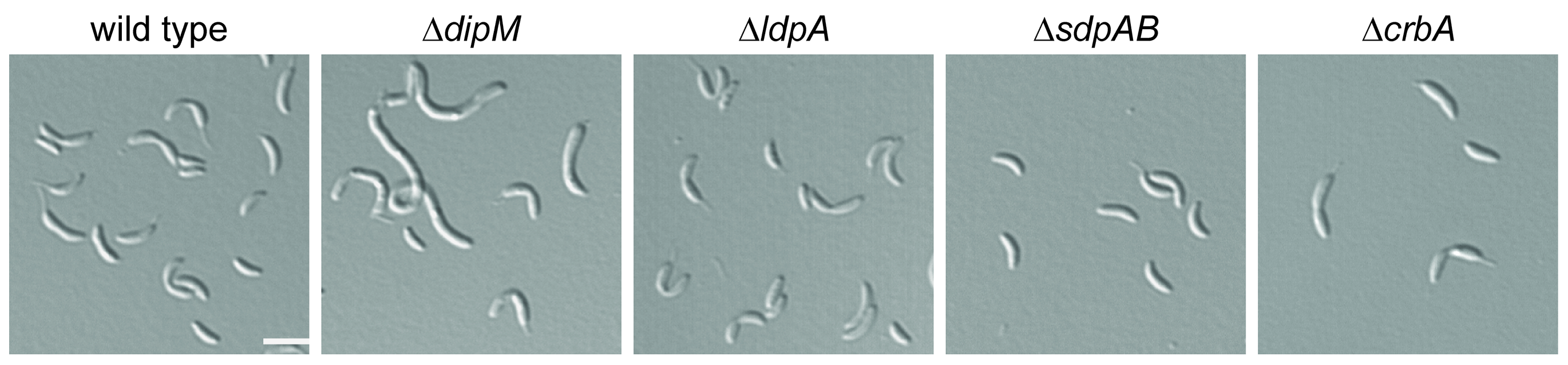

Supplement: S7 Fig — Cells of strains NA1000 (WT), MAB360 (ΔdipM Pxyl::Pxyl-dipM), AM364 (ΔldpA), AZ22 (ΔsdpAB), and AM376 (ΔcrbA) were cultivated in PYE medium and visualized by DIC microscopy. (TIF) [file pgen.1007897.s007.tif]

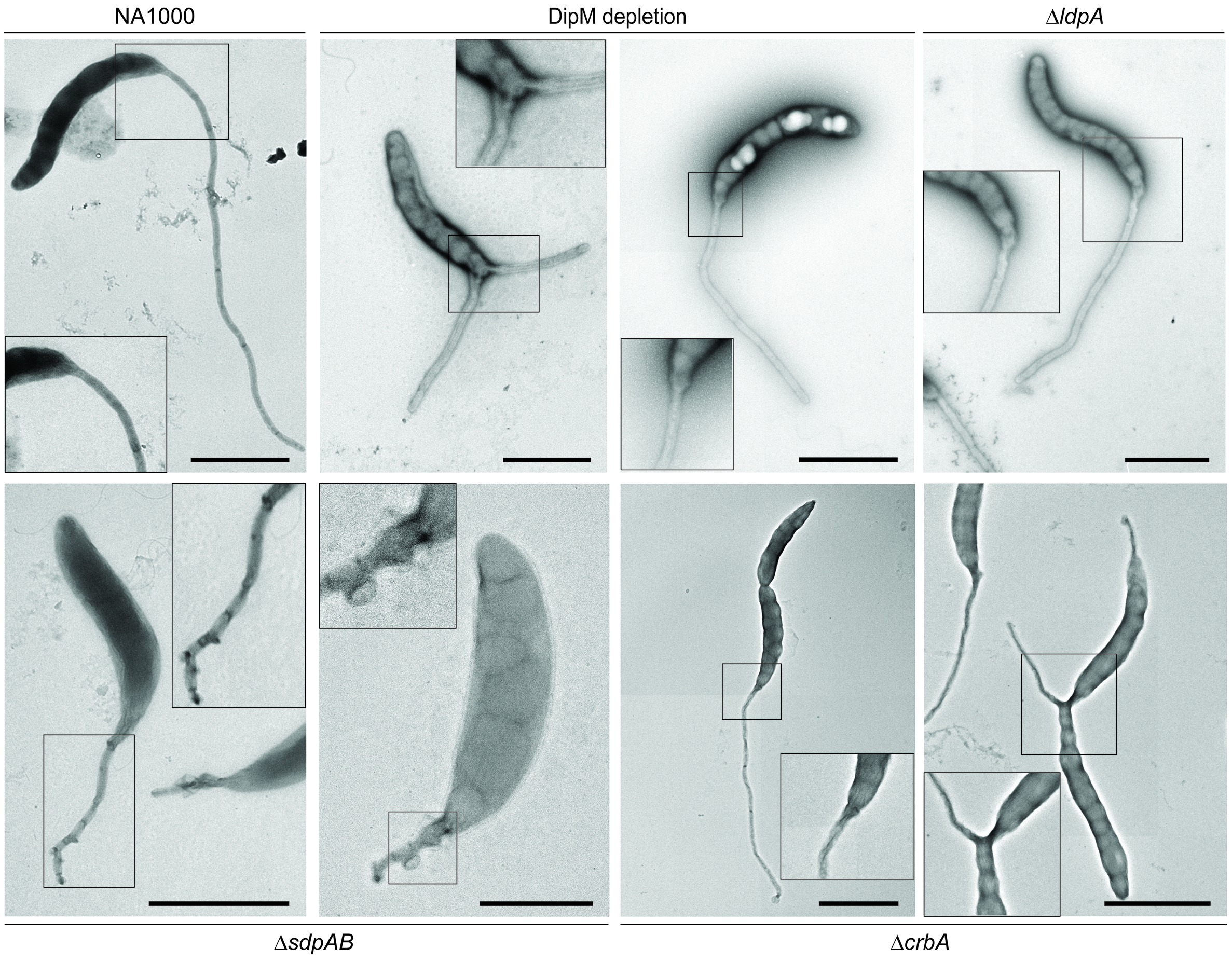

Supplement: S8 Fig — Cells of strains NA1000 (WT), MAB360 (ΔdipM Pxyl::Pxyl-dipM), AM364 (ΔldpA), AZ22 (ΔsdpAB), and AM376 (ΔcrbA) were grown for 24 h in M2G-P medium, stained with uranyl acetate, and visualized by transmission electron microscopy (scale bars: 2 μm). (TIF) [file pgen.1007897.s008.tif]

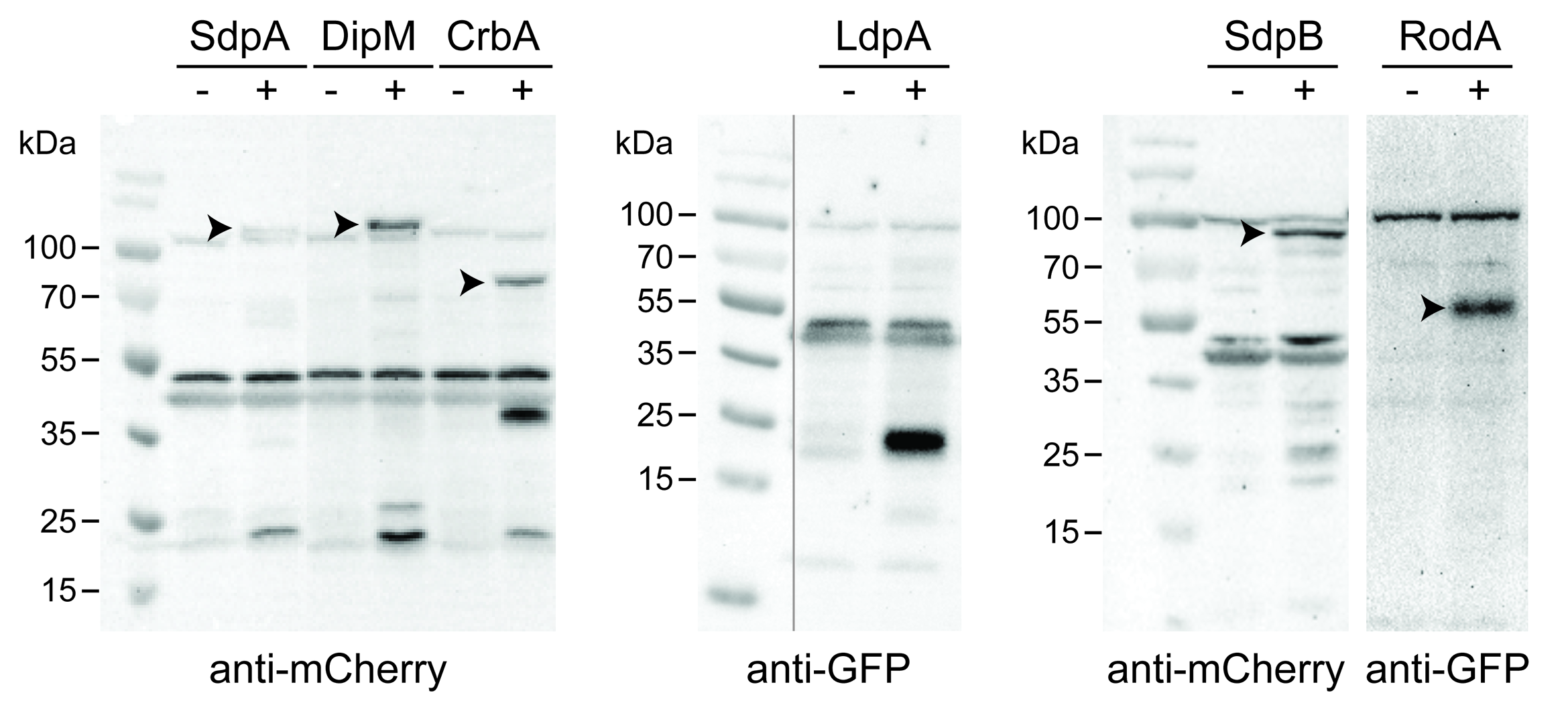

Supplement: S9 Fig — Cells producing fluorescently tagged derivatives of SdpA (AM480, Pxyl::Pxyl-sdpA-mCherry), DipM (AM208, Pxyl::Pxyl-dipM-mCherry), CrbA (MAB247, Pxyl::Pxyl-crbA-mCherry), LdpA (MAB293, Pxyl::Pxyl-mCherry-ldpA), SdpB (AZ127, Pxyl::Pxyl-torA’-sdpB-mCherry), and RodA (MAB405, Pxyl::Pxyl-gfp-rodA) were grown for 24 h in M2G-P medium and subjected to Western blot analysis. Four hours (MAB293), three hours (AM480, MAB247, AM208, MAB405) or two hours (AZ127) prior to analysis, the media were supplemented with 0.3% xylose to induce the synthesis of the fusion proteins (+). Cells grown in the absence of xylose (-) are shown as controls. (TIF) [file pgen.1007897.s009.tif]

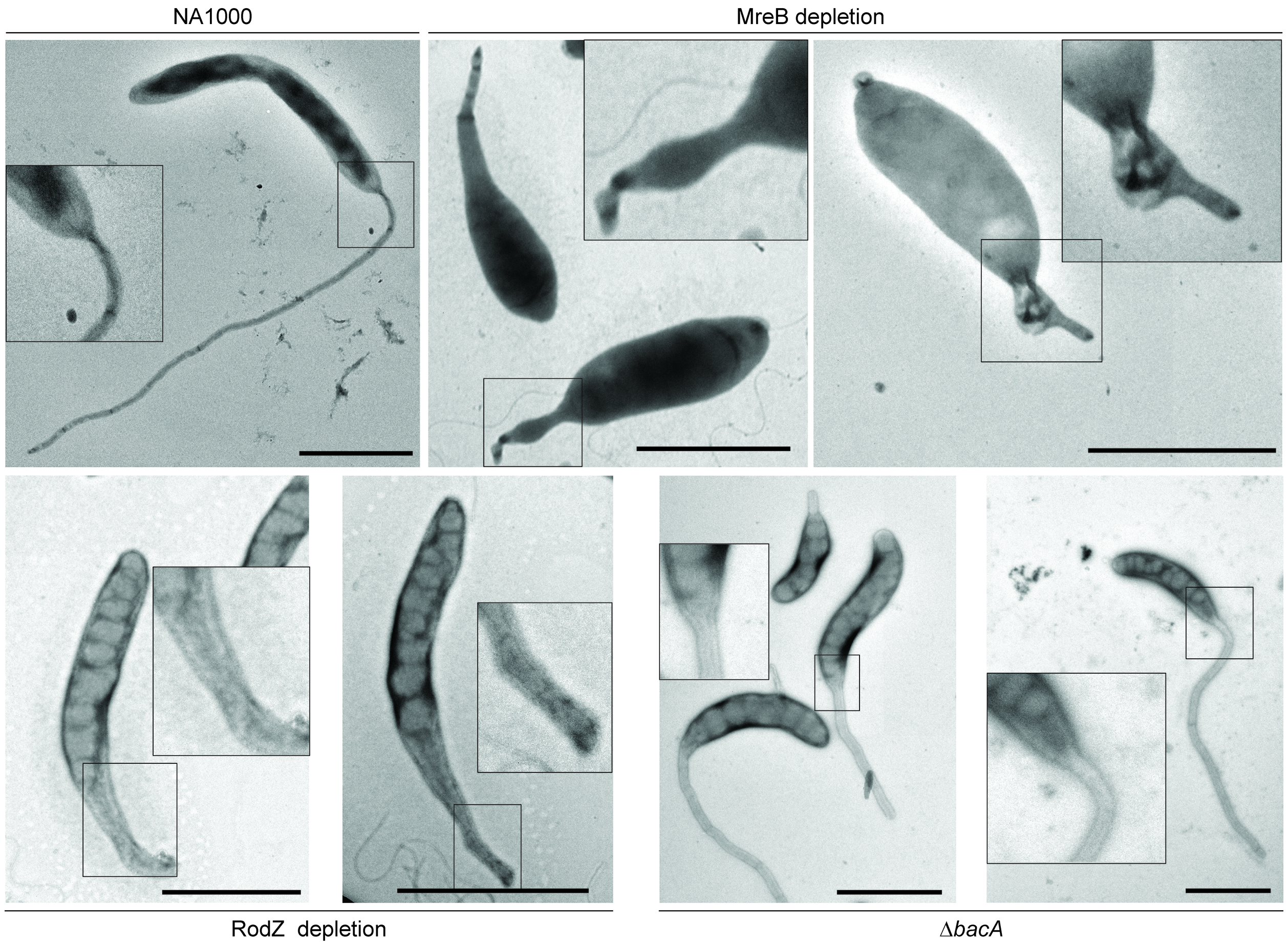

Supplement: S10 Fig — Shown are cells of strains NA1000 (WT), LS3809 (ΔmreB Pxyl::Pxyl-mreB), CJW2747 (ΔrodZ::Ω Pxyl::Pxyl-rodZ), and MT257 (ΔbacA) that were stained with uranyl acetate and visualized by transmission electron microscopy (scale bars: 2 μm). Strains NA1000 and MT257 (ΔbacA) was grown in M2G-P for 24 h prior to imaging. Strains LS3809, and CJW2747 were grown to exponential phase in PYE medium containing the inducer xylose, washed, cultivated for 7 h in inducer-free PYE medium, and then diluted (1:20) into M2G-P medium 24 h prior to imaging. (TIF) [file pgen.1007897.s010.tif]

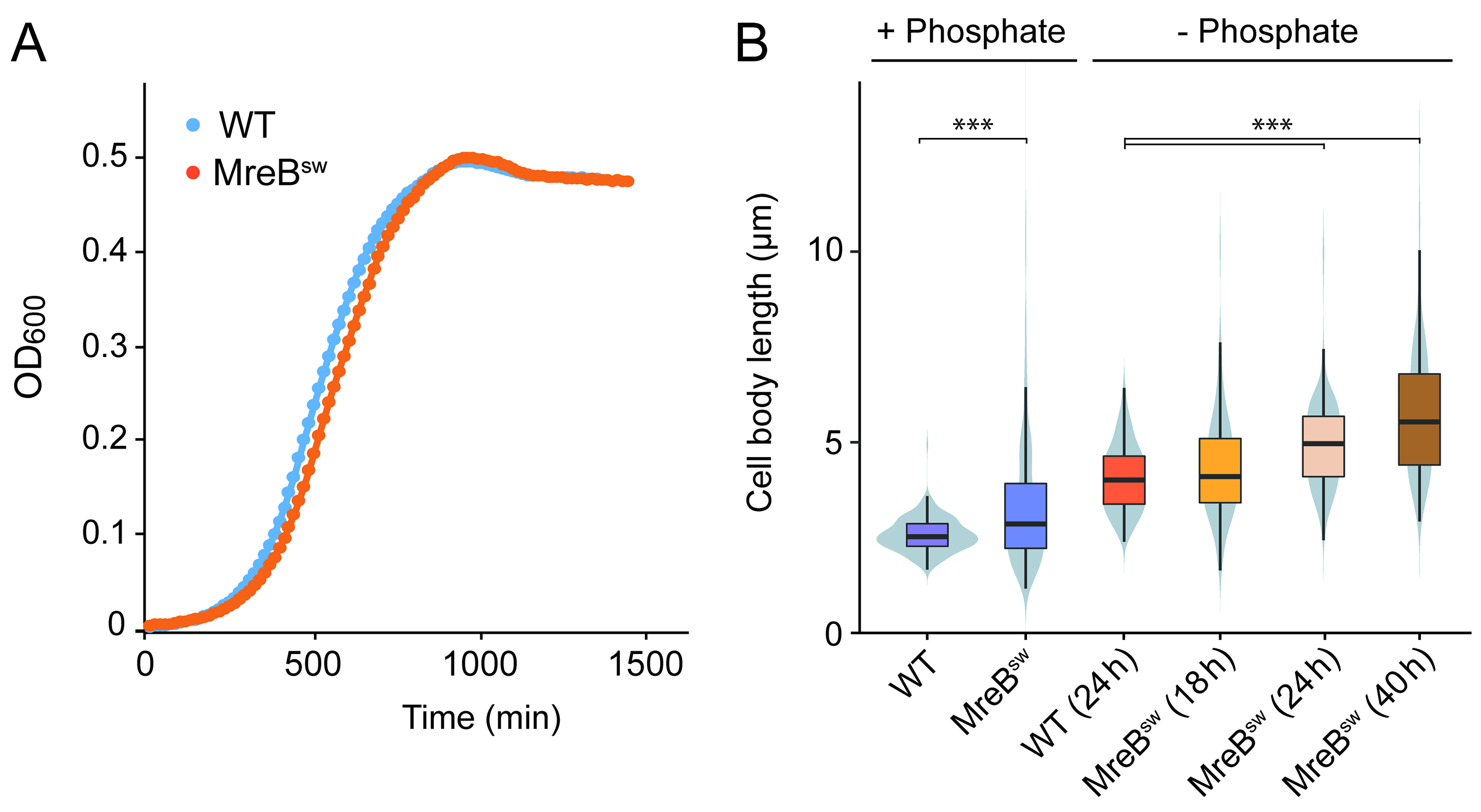

Supplement: S11 Fig — (A) Growth curves of strains NA1000 (WT) and MAB238 (mreBSW) in PYE medium. (B) Distribution of the cell body lengths in populations of strains NA1000 (WT) and MAB238 (mreBSW) during exponential growth in PYE medium or after cultivation for 18 h, 24 h and 40 h in M2G-P medium. The values obtained (n = 208 per strain) are shown as box plots, with the thick line indicating the median, the box the interquartile range and the wiskers the 2nd and the 98th percentile. In addition rotated kernel density plots (grey) are depicted for each dataset to indicate the distribution of the raw data (*** p < 10−6; t-test). See S1 File for the raw data. (TIF) [file pgen.1007897.s011.tif]
