# Supplementary material for: A specialized MreB-dependent cell wall biosynthetic complex mediates the formation of stalk-specific peptidoglycan in Caulobacter crescentus
Source: PLoS Genet. 2019 Feb 1;15(2):e1007897. doi: 10.1371/journal.pgen.1007897 (PMC6373972; doi:10.1371/journal.pgen.1007897)
Supplement: S1 Table — The indicated strains were analyzed after growth for 24 h in M2G-P. Values are the mean ± variance of two independent experiments. (PDF) [file pgen.1007897.s012.pdf]

Table S1. Composition of cell body and stalk peptidoglycan from from crossband- and LD-transpeptidase-deficient cells. The indicated strains were analyzed after growth for 24 h in M2G^{-P}. Values are the mean ± variance of two independent experiments.

Muropeptide	Relative percentage (%) in strain					
	WT (CB15N)		Δ*stpAB*		Δ*ldtDX*	
	Cell body	Stalk	Cell body	Stalk	Cell body	Stalk
Tri	**0.8**±0.0	**1.4**±0.0	**1.5**±0.0	**3.3**±0.0	n.d.	n.d.
Tetra	**16.5**±0.4	**11.1**±6.6	**19.3**±0.3	**12.1**±3.5	**19.3**±0.1	**13.6**±0.0
Penta	**7.9**±0.0	**6.5**±0.1	**8.9**±0.0	**6.6**±0.0	**8.6**±0.0	**7.8**±0.0
TriTri (LD)	**0.3**±0.0	**0.9**±0.0	**0.2**±0.1	**1.2**±0.2	n.d.	n.d.
TetraTri (LD)	**1.0**±0.1	**2.9**±0.1	**0.6**±0.0	**2.2**±0.2	n.d.	n.d.
TriAnh/TetraTri (LD)	**1.1**±0.0	**1.3**±0.0	**1.1**±0.0	**1.9**±0.0	n.d.	n.d.
TetraTetra(LD)	**1.3**±1.1	**1.5**±0.0	**0.4**±0.0	**1.4**±0.0	**0.2**±0.0	**0.2**±0.0
TetraTetra	**12.7**±0.0	**10.8**±1.5	**14.6**±0.1	**10.4**±0.1	**14.3**±0.0	**13.5**±0.0
TetraPenta	**6.8**±0.1	**7.0**±0.0	**7.9**±0.0	**6.9**±0.0	**7.4**±0.0	**8.5**±0.0
TetraTetraTri or TetraTetraTri (LD)	**0.3**±0.0	**1.2**±0.0	**0.2**±0.0	**1.0**±0.0	n.d.	n.d
TetraAnh	**1.4**±0.0	**1.7**±0.0	**1.1**±0.0	**0.8**±0.0	**1.2**±0.0	**1.2**±0.0
TetraTetraTetra	**3.8**±0.0	**5.1**±0.0	**4.1**±0.0	**4.8**±0.1	**4.2**±0.0	**5.9**±0.0
TetraTetraPenta	**1.8**±0.0	**3.0**±0.0	**1.9**±0.0	**2.6**±0.0	**2.1**±0.0	**3.2**±0.0
TetraTetraTetraTetra	n.d.	**0.3**±0.2	n.d.	**0.5**±0.0	n.d.	n.d.
TetraTetraTetraPenta	n.d.	**0.6**±0.2	n.d.	**0.3**±0.0	n.d.	n.d.
PentaAnh	**0.8**±0.0	**1.6**±0.2	**0.7**±0.0	**1.4**±0.0	**0.8**±0.0	**1.7**±0.0
TetraTetraAnh	**1.0**±0.0	**2.0**±0.0	**0.7**±0.2	**1.1**±0.0	**0.8**±0.0	**0.6**±0.0
TetraPentaAnh	**3.6**±0.0	**3.1**±0.0	**2.3**±0.0	**1.9**±0.0	**2.5**±0.0	**2.6**±0.0
TetraTetraTriAnh or TetraTetraTri(LD)Anh	**0.6**±0.0	**1.3**±0.0	**0.7**±0.0	**1.8**±0.0	**1.3**±0.0	**1.9**±0.0
TetraTetraTetraAnh	**6.4**±0.1	**6.2**±0.0	**6.8**±0.0	**6.2**±0.0	**6.8**±0.0	**6.8**±0.0
TetraTetraPentaAnh	**6.6**±0.1	**6.2**±0.0	**6.4**±0.0	**5.7**±0.1	**6.7**±0.0	**6.6**±0.0
TetraTetraTetraPentaAnh I	**1.4**±0.0	**1.0**±0.0	**1.3**±0.0	**1.1**±0.2	**1.4**±0.0	**2.0**±0.0
TetraTetraTetraPentaAnh II	**1.4**±0.0	**1.3**±0.0	**1.5**±0.0	**1.2**±0.0	**1.7**±0.0	**1.8**±0.0
TetraTetraTetradiAnh	**1.4**±0.0	**1.5**±0.0	**1.8**±0.0	**1.4**±0.5	**1.4**±0.0	**1.6**±0.7
TetraTetraTetraPentadiAnh	**1.8**±0.3	**1.3**±0.0	**1.9**±0.2	**1.1**±0.0	**2.3**±0.0	**1.6**±0.3
TetraTetradiAnh	**1.8**±0.0	**1.3**±0.0	**1.2**±0.4	**1.0**±0.0	**2.7**±0.0	**1.6**±0.0
TetraTetraPentadiAnh	**0.7**±0.0	**0.4**±0.0	**0.7**±0.0	**0.4**±0.0	**0.9**±0.0	**0.8**±0.0
all known	**83.3**±0.6	**82.5**±4.7	**87.7**±0.5	**80.3**±0.0	**86.5**±0.0	**83.4**±0.2
Monomers (total)	**33.0**±0.1	**27.0**±4.5	**36.0**±1.1	**30.1**±4.3	**34.6**±0.3	**29.0**±0.4
Dimers (total)	**35.6**±3.4	**37.2**±0.0	**32.9**±0.7	**34.8**±1.6	**32.2**±0.0	**32.3**±0.0
Trimers (total)	**26.0**±0.5	**30.2**±3.7	**25.9**±0.1	**29.9**±4.1	**27.0**±0.1	**32.2**±1.7
Tetramers (total)	**5.4**±0.6	**5.5**±0.2	**5.3**±0.2	**5.2**±1.4	**6.2**±0.0	**6.5**±0.4
Tripeptides (total)	**3.0**±0.0	**6.4**± 0.6	**3.1**±0.0	**9.3**±1.3	**0.5**±0.0	**0.8**±0.0
Tetrapeptides (total)	**74.7**±0.0	**71.9**±2.5	**74.5**±0.3	**69.2**±1.3	**77.6**±0.0	**75.4**±0.2
Pentapeptides (total)	**21.7**±0.0	**21.1**±0.5	**21.6**±0.0	**20.2**±0.0	**21.5**±0.0	**23.2**±0.2
3-3 crosslinks	**2.3**±0.6	**5.2**±0.7	**1.0**±0.0	**4.2**±0.6	**0.6**±0.0	**0.9**±0.0
Chain ends (Anh)	**16.5**±0.2	**17.2**±0.9	**14.2**±1.2	**14.5**±0.8	**17.0**±0.0	**17.4**±0.3
Degree of cross-linkage	**39.2**±0.0	**42.9**±2.2	**37.6**±0.4	**41.2**±1.2	**38.8**±0.1	**42.5**±0.2
% peptides in cross-links	**67.0**±0.1	**73.0**±4.5	**64.0**±1.1	**69.9**±4.3	**65.4**±0.3	**71.0**±0.4
	Mean of disaccharides units ± SD (n=2)					
Average glycan chain length	**6.0**±0.1	**5.8**±0.1	**7.1**±0.3	**6.9**±0.2	**5.9**±0.0	**5.8**±0.0
