# Supplementary material for: A specialized MreB-dependent cell wall biosynthetic complex mediates the formation of stalk-specific peptidoglycan in Caulobacter crescentus
Source: PLoS Genet. 2019 Feb 1;15(2):e1007897. doi: 10.1371/journal.pgen.1007897 (PMC6373972; doi:10.1371/journal.pgen.1007897)
Supplement: S2 Table — (PDF) [file pgen.1007897.s013.pdf]

Table S2. List of peptidoglycan biosynthesis mutants analyzed for stalk defects.

LytM-like endopeptidases	
**Locus Tags**	*ldpA* (CC1872, CCNA_01948), *ldpB* (CC2248, CCNA_02331), *ldpC* (CC2297, CCNA_02382), *ldpD* (CC3034 CCNA_03129), *ldpE* (CC3301, CCNA_03410), *ldpF* (CC3434, CCNA_03547), *dipM* (CC1996 CCNA_02075)
**Single deletion**	*ΔldpA*, *ΔldpB*, *ΔldpC*, *ΔldpD*, *ΔldpE*, *ΔldpF*, *ΔdipM*
**Multiple deletions**	*ΔldpA ΔldpB ΔldpC ΔldpD ΔldpE*, *ΔldpA ΔldpB*, *ΔldpB ΔldpF*, *ΔldpA ΔldpB ΔldpC*, *ΔldpA ΔldpC ΔldpD*, *ΔldpC ΔldpD ΔldpE*, *ΔldpA ΔldpB ΔldpC ΔldpD ΔldpE ΔldpF*
NlpC/P60 and CHAP domain proteins	
**Locus Tags**	CC2936 (CCNA_03031), CC0349 (CCNA_00354), CC2775 (CCNA_02863)
**Single deletions**	ΔCC2936, ΔCC0349, ΔCC2775
**Multiple deletions**	ΔCC2775 ΔCC2936, ΔCC2936 ΔCC0349 ΔCC2775
Soluble lytic transglycosylases	
**Locus Tags**	*sdpA* (CC1194, CCNA_01252), *sdpB* (CC1332, CCNA_01393), *sdpC* (CC2416, CCNA_02498)
**Single deletions**	*ΔsdpA*, *ΔsdpB*, *ΔsdpC*
**Multiple deletions**	*ΔsdpB ΔsdpC*, *ΔsdpA ΔsdpC*, *ΔsdpA ΔsdpB*
Membrane-bound lytic transglycosylases	
**Locus Tags**	CC3740 (CCNA_03856), CC3322 (CCNA_03431)
**Single deletions**	ΔCC3740, ΔCC3322
**Multiple deletions**	ΔCC3740 ΔCC3322
Amidase	
**Locus Tags**	*amiC* (CC1876, CCNA_01952)
**Single depletions**	*ΔamiC* Pxyl::Pxyl-*amiC*
Carboxypeptidase	
**Locus Tags**	*crbA* (CC2161, CCNA_02243)
**Single deletions**	ΔCC2161
LD-transpeptidases	
**Locus Tags**	*ldtD* (CC1511, CCNA_01579), *ldtX* (CC3744, CCNA_03860)
**Single deletions**	*ΔldtD*, *ΔldtX*
**Multiple deletions**	*ΔldtD ΔldtX*
Penicillin-binding proteins (class A)	
**Locus Tags**	*pbpY* (CC1875, CCNA_01951), *pbp1A* (CC1516, CCNA_01584), *pbpC* (CC3277, CCNA_03386), *pbpZ* (CC3570, CCNA_03685), *pbpX* (CC0252, CCNA_00252)
**Single deletions**	*ΔpbpC*, *ΔpbpX*
**Multiple deletions**	*Δpbp1a ΔpbpC ΔpbpY ΔpbpZ*, *Δpbp1a ΔpbpY ΔpbpZ*, *Δpbp1a ΔpbpC ΔpbpY ΔpbpZ Pxyl::Pxyl-pbpX*
