# Supplementary material for: A specialized MreB-dependent cell wall biosynthetic complex mediates the formation of stalk-specific peptidoglycan in Caulobacter crescentus
Source: PLoS Genet. 2019 Feb 1;15(2):e1007897. doi: 10.1371/journal.pgen.1007897 (PMC6373972; doi:10.1371/journal.pgen.1007897)
Supplement: S3 Table — The indicated strains were analyzed after growth for 24 h in M2G-P. The values are the mean ± variance of two independent experiments. (PDF) [file pgen.1007897.s014.pdf]

**Table S3. Composition of peptidoglycan isolated from autolysin-deficient cells.** The indicated strains were analyzed after growth for 24 h in M2G$^{-P}$. The values are the mean ± variance of two independent experiments.

| Muropeptide | Relative percentage (%) in strain | | | | |
|---|---|---|---|---|---|
| | WT | Δ*dipM* | Δ*crbA* | Δ*ldpA* | Δ*sdpAB* |
| Tri | 0.9±0.0 | 1.8±0.1 | 0.6±0.0 | 1.1±0.0 | 4.3±2.1 |
| Tetra | 17.5±0.0 | 21.3±0.5 | 9.8±0.5 | 18.2±0.1 | 27.5±1.8 |
| Penta | 7.9±0.1 | 8.1±0.3 | 11.0±0.0 | 7.4±0.0 | 6.6±0.0 |
| TriTri (LD) | 0.2±0.0 | 0.2±0.0 | 0.2±0.0 | 0.2±0.0 | 0.5±0.0 |
| TetraTri (LD) | 1.1±0.0 | 0.9±0.0 | 0.2±0.0 | 0.8±0.0 | 1.9±0.0 |
| TriAnh +small amount of TetraTri (LD) | 0.9±0.0 | 1.0±0.0 | 0.5±0.0 | 0.9±0.0 | 1.6±0.3 |
| TetraTetra (LD) | 0.2±0.0 | 0.5±0.0 | 1.1±0.0 | 0.9±0.5 | 0.6±0.0 |
| TetraTetra | 15.1±0.0 | 15.9±0.0 | 10.8±0.3 | 15.8±0.0 | 16.7±0.8 |
| TetraPenta | 7.3±0.2 | 7.3±0.1 | 12.1±0.1 | 7.1±0.1 | 5.3±0.0 |
| TetraTetraTri or TetraTetraTri (LD) | 0.4±0.0 | 0.3±0.0 | 0.3±0.0 | 0.3±0.0 | 0.4±0.0 |
| TetraAnh | 0.3±0.0 | 0.5±0.0 | 0.2±0.0 | 0.5±0.0 | 0.8±0.0 |
| TetraTetraTetra | 4.4±0.1 | 4.5±0.0 | 3.5±0.2 | 4.6±0.0 | 5.5±0.2 |
| TetraTetraPenta | 1.8±0.0 | 1.8±0.0 | 3.6±0.0 | 1.8±0.0 | 1.5±0.0 |
| TetraTetraTetraTetra | 0.8±0.0 | 0.8±0.0 | 0.6±0.0 | 0.8±0.0 | 1.1±0.0 |
| TetraTetraTetraPenta | 0.4±0.0 | 0.4±0.0 | 0.6±0.0 | 0.4±0.0 | 0.4±0.0 |
| PentaAnh | 0.8±0.0 | 0.8±0.0 | 0.8±0.0 | 0.8±0.0 | 1.7±0.0 |
| TetraTetraAnh | 4.3±0.0 | 3.9±0.0 | 3.0±0.0 | 4.1±0.0 | 2.6±0.0 |
| TetraPentaAnh | 0.9±0.0 | 0.8±0.0 | 0.4±0.1 | 0.6±0.0 | 0.9±0.1 |
| TetraTetraTriAnh or TetraTetraTri(LD)Anh | n.d. | n.d. | n.d. | n.d. | n.d. |
| TetraTetraTetraAnh | 7.6±0.1 | 7.1±0.0 | 5.6±0.0 | 7.6±0.0 | 6.4±0.0 |
| TetraTetraPentaAnh | 6.4±0.5 | 5.9±0.1 | 7.0±0.0 | 7.0±0.0 | 4.0±0.0 |
| TetraTetraTetraPentaAnh I | 1.2±0.0 | 1.2±0.0 | 1.3±0.0 | 1.3±0.0 | 1.0±0.0 |
| TetraTetraTetraPentaAnh II | 1.3±0.0 | 1.3±0.0 | 2.3±0.0 | 1.4±0.0 | 0.7±0.0 |
| TetraTetraTetradiAnh | 1.1±0.0 | 1.0±0.0 | 1.1±0.0 | 1.2±0.0 | 0.5±0.0 |
| TetraTetraTetraPentadiAnh | 0.9±0.0 | 0.9±0.0 | 1.3±0.0 | 1.0±0.0 | 0.3±0.0 |
| TetraTetradiAnh | 1.5±0.0 | 1.4±0.0 | 1.8±0.0 | 1.5±0.0 | 0.4±0.0 |
| TetraTetraPentadiAnh | 0.7±0.0 | 0.6±0.0 | 1.2±0.0 | 0.7±0.0 | 0.3±0.0 |
| | | | | | |
| all known | 85.7±1.7 | 90.1±1.0 | 80.6±0.0 | 87.9±0.1 | 93.4±0.5 |
| Monomers (total) | 32.9±0.5 | 37.3±2.1 | 28.4±0.2 | 32.9±0.1 | 45.4±1.5 |
| Dimers (total) | 35.7±1.7 | 34.1±0.0 | 36.5±1.4 | 35.2±1.1 | 31.0±0.2 |
| Trimers (total) | 26.0±1.5 | 23.6±1.1 | 27.6±1.3 | 26.5±0.1 | 20.0±0.4 |
| Tetramers (total) | 5.4±0.6 | 5.1±0.3 | 7.4±0.2 | 5.5±0.3 | 3.7±0.0 |
| Tripeptides (total) | 3.1±0.0 | 4.0±0.2 | 1.8±0.3 | 3.0±0.0 | 8.1±5.4 |
| Tetrapeptides (total) | 77.1±0.2 | 77.0±0.6 | 68.9±0.6 | 77.9±0.0 | 76.3±6.9 |
| Pentapeptides (total) | 19.5±0.1 | 18.5±0.2 | 29.0±0.0 | 18.5±0.1 | 14.8±0.0 |
| 3-3 crosslinks | 1.0±0.0 | 1.0±0.0 | 1.0±0.0 | 1.2±0.2 | 1.8±0.0 |
| Chain ends (Anh) | 15.0±0.2 | 13.9±0.1 | 15.2±0.4 | 15.1±0.0 | 11.6±1.0 |
| Degree of cross-linkage | 39.2±0.6 | 36.6±1.1 | 42.2±0.2 | 39.3±0.0 | 31.6±0.5 |
| % Peptides in crosslinks | 67.1±0.5 | 62.7±2.1 | 71.6±0.2 | 67.1±0.1 | 54.6±1.5 |
| | Mean of disaccharides units ± SD (n=2) | | | | |
| Average glycan chain length | 6.7±0.0 | 7.2±0.0 | 6.6±0.1 | 6.6±0.0 | 8.6±0.6 |
