# Supplementary material for: A specialized MreB-dependent cell wall biosynthetic complex mediates the formation of stalk-specific peptidoglycan in Caulobacter crescentus
Source: PLoS Genet. 2019 Feb 1;15(2):e1007897. doi: 10.1371/journal.pgen.1007897 (PMC6373972; doi:10.1371/journal.pgen.1007897)
Supplement: S4 Table — Strains MT257 (ΔbacA), MAB238 (mreB::mreBsw), and NA1000 (WT) were grown for 24 h in M2G-P prior to analysis. Strains CJW2747 (ΔrodZ::Ω Pxyl::Pxyl-rodZ) and LS3809 (ΔmreB Pxyl::Pxyl-mreB) were first cultivated for 7 h in PYE and then grown for 24 h in M2G-P. Values are the mean ± variance of two independent experiments. (PDF) [file pgen.1007897.s015.pdf]

Table S4. Composition of peptidoglycan isolated from cells with defects in scaffolding proteins. Strains MT257 (Δ*bacA*), MAB238 (*mreB::mreB*^{sw}), and NA1000 (WT) were grown for 24 h in M2G^{-P} prior to analysis. Strains CJW2747 (Δ*rodZ::Ω* Pxyl::Pxyl-*rodZ*) and LS3809 (Δ*mreB* Pxyl::Pxyl-*mreB*) were first cultivated for 7 h in PYE and then grown for 24 h in M2G^{-P}. Values are the mean ± variance of two independent experiments.

Muropeptide	Relative percentage (%) in strain				
	**WT**	**Δ*bacA***	**RodZ depl.**	**MreB depl.**	**MreB^{sw}**
**Tri**	**0.9**±0.0	**1.4**±0.0	**5.0**±0.0	**5.1**±0.1	**3.8**±0.0
**Tetra**	**17.5**±0.0	**19.6**±0.0	**17.7**±0.2	**17.4**±0.0	**19.2**±0.1
**Penta**	**7.9**±0.1	**8.0**±0.1	**8.3**±0.0	**8.7**±0.0	**7.3**±0.0
**TriTri (LD)**	**0.2**±0.0	**0.2**±0.0	**0.8**±0.0	**1.0**±0.0	**0.5**±0.0
**TetraTri (LD)**	**1.1**±0.0	**0.7**±0.0	**1.9**±0.0	**2.5**±0.0	**1.9**±0.0
**TriAnh/TetraTri (LD)**	**0.9**±0.0	**1.1**±0.0	**3.6**±0.0	**3.7**±0.0	**3.0**±0.0
**TetraTetra (LD)**	**0.2**±0.0	**0.4**±0.0	**1.1**±0.0	**1.3**±0.0	**0.9**±0.0
**TetraTetra**	**15.1**±0.0	**16.8**±0.2	**13.6**±0.0	**13.3**±0.1	**15.8**±0.0
**TetraPenta**	**7.3**±0.2	**7.7**±0.0	**6.4**±0.0	**6.8**±0.0	**5.9**±0.0
**TetraTetraTri or TetraTetraTri (LD)**	**0.4**±0.0	**0.3**±0.0	**0.6**±0.0	**0.7**±0.0	**0.6**±0.0
**TetraAnh**	**0.3**±0.0	**0.5**±0.0	**0.9**±0.0	**0.8**±0.0	**0.8**±0.0
**TetraTetraTetra**	**4.4**±0.1	**5.0**±0.0	**3.7**±0.0	**3.1**±0.0	**4.4**±0.0
**TetraTetraPenta**	**1.8**±0.0	**1.9**±0.0	**1.4**±0.0	**1.3**±0.0	**1.3**±0.0
**TetraTetraTetraTetra**	**0.8**±0.0	**0.9**±0.0	**0.3**±0.0	**0.3**±0.0	**0.2**±0.0
**TetraTetraTetraPenta**	**0.4**±0.0	**0.4**±0.0	**0.1**±0.0	**0.2**±0.0	**0.1**±0.0
**PentaAnh**	**0.8**±0.0	**0.9**±0.0	**0.6**±0.0	**0.5**±0.0	**0.7**±0.0
**TetraTetraAnh**	**4.3**±0.0	**4.2**±0.0	**0.7**±0.0	**0.7**±0.0	**0.6**±0.0
**TetraPentaAnh**	**0.9**±0.0	**0.8**±0.0	**2.2**±0.0	**2.2**±0.0	**2.2**±0.0
**TetraTetraTriAnh or TetraTetraTri(LD)Anh**	n.d.	n.d.	**1.5**±0.0	**1.5**±0.0	**1.4**±0.0
**TetraTetraTetraAnh**	**7.6**±0.1	**7.0**±0.0	**5.5**±0.0	**4.9**±0.1	**6.1**±0.0
**TetraTetraPentaAnh**	**6.4**±0.5	**6.1**±0.0	**4.3**±0.1	**4.1**±0.0	**4.7**±0.0
**TetraTetraTetraPentaAnh I**	**1.2**±0.0	**1.3**±0.0	**0.6**±0.0	**0.4**±0.0	**0.7**±0.0
**TetraTetraTetraPentaAnh II**	**1.3**±0.0	**1.3**±0.0	**0.6**±0.0	**0.5**±0.0	**0.7**±0.0
**TetraTetraTetradiAnh**	**1.1**±0.0	**1.0**±0.0	**1.0**±0.0	**0.7**±0.0	**0.9**±0.0
**TetraTetraTetraPentadiAnh**	**0.9**±0.0	**0.9**±0.0	**1.3**±0.0	**1.0**±0.0	**1.3**±0.0
**TetraTetradiAnh**	**1.5**±0.0	**1.2**±0.0	**1.3**±0.0	**1.1**±0.0	**1.2**±0.0
**TetraTetraPentadiAnh**	**0.7**±0.0	**0.6**±0.0	**0.4**±0.0	**0.4**±0.0	**0.3**±0.0
**all known**	**85.7**±1.7	**90.2**±0.0	**85.3**±0.1	**84.4**±0.2	**86.4**±0.1
**Monomers (total)**	**32.9**±0.5	**35.1**±0.0	**38.2**±0.6	**38.6**±0.3	**36.8**±0.2
**Dimers (total)**	**35.7**±1.7	**35.4**±0.0	**36.9**±0.1	**38.7**±0.0	**36.8**±0.0
**Trimers (total)**	**26.0**±1.5	**24.3**±0.0	**21.5**±0.2	**19.7**±0.1	**22.8**±0.1
**Tetramers (total)**	**5.4**±0.6	**5.2**±0.0	**3.4**±0.0	**2.9**±0.0	**3.5**±0.0
**Tripeptides (total)**	**3.1**±0.0	**3.5**±0.0	**10.8**±0.0	**11.8**±0.4	**8.5**±0.1
**Tetrapeptides (total)**	**77.1**±0.2	**77.0**±0.0	**70.6**±0.0	**69.1**±0.6	**74.3**±0.0
**Pentapeptides (total)**	**19.5**±0.1	**18.9**±0.0	**18.6**±0.0	**19.1**±0.0	**17.2**±0.0
**3-3 crosslinks**	**1.0**±0.0	**0.8**±0.0	**3.0**±0.0	**3.7**±0.0	**2.6**±0.0
**Chain ends (Anh)**	**15.0**±0.2	**14.1**±0.0	**11.3**±0.0	**10.4**±0.0	**11.4**±0.1
**Degree of cross-linkage**	**39.2**±0.6	**37.8**±0.0	**35.3**±0.2	**34.7**±0.1	**36.3**±0.1
**% peptides in cross-links**	**67.1**±0.5	**64.9**±0.0	**61.8**±0.6	**61.4**±0.3	**63.2**±0.2
	Mean of disaccharides units ± standard deviation (n=2)				
**Average glycan chain length**	**6.7**±0.0	**7.1**±0.0	**8.8**±0.0	**9.6**±0.0	**8.8**±0.0
