# Supplementary material for: A specialized MreB-dependent cell wall biosynthetic complex mediates the formation of stalk-specific peptidoglycan in Caulobacter crescentus
Source: PLoS Genet. 2019 Feb 1;15(2):e1007897. doi: 10.1371/journal.pgen.1007897 (PMC6373972; doi:10.1371/journal.pgen.1007897)
Supplement: S5 Table — (DOCX) [file pgen.1007897.s016.docx]

**Table S5. *C. crescentus* strains used in this study.**

| **Strain** | **Genotype/description** | **Construction** | **References** |
| --- | --- | --- | --- |
| CB15N | Synchronizable wild-type strain | - | Evinger & Agabian (1977) |
| AM208 | CB15N P*_xyl_::*P*_xyl_-dipM-mCherry* | - | Möll *et al.* (2010) |
| AM364 | CB15N Δ*ldpA* | - | Zielinska *et al.* (2017) |
| AM376 | CB15N Δ*crbA* (*CCNA_02243*) | In-frame deletion of CCNA_02243 in CB15N using pAM152 | This study |
| AM457 | CB15N P_xyl_::P_xyl_-*venus-pbpY* | - | Strobel *et al.,* (2014) |
| AM458 | CB15N P_xyl_::P_xyl_-*venus-pbpZ* | - | Strobel *et al.* (2014) |
| AM480 | CB15N P_xyl_::P_xyl_-*sdpA-mCherry* | - | Zielinska *et al.* (2017) |
| AZ22 | CB15N Δ*sdpAB* | - | Zielinska *et al.* (2017) |
| AZ52 | CB15N Δ*ldpABCDEF* | - | Zielinska *et al.* (2017) |
| AZ85 | CB15N Δ*CCNA_02863* | In-frame deletion of CCNA_02863 in CB15N using pAZ12 | This study |
| AZ89 | CB15N Δ*CCNA_02863* Δ*CCNA_00354* | In-frame deletion of CCNA_00354 in AZ85 using pAZ11 | This study |
| AZ127 | CB15N P_xyl_::P_xyl_*-torA’-sdpB-mCherry* | - | Zielinska *et al.* (2017) |
| AZ137 | CB15N Δ*CCNA_01579* (*ldtD*) | In-frame deletion of CCNA_01579 in CB15N using pAZ26 | This study |
| AZ138 | CB15N Δ*ldtD* Δ*ldtX* | In-frame deletion of CCNA_03860 in AZ137 using pAZ30 | This study |
| AZ140 | CB15N Δ*CCNA_03860* (*ldtX*) | In-frame deletion of CCNA_03860 in CB15N using pAZ30 | This study |
| CJW2745 | CB15N *rodZ::cfp-rodZ* | - | Alyahya *et al.* (2009) |
| CJW2747 | CB15N Δ*rodZ::Ω* P_xyl_::P_xyl_*-rodZ* | - | Alyahya *et al.* (2009) |
| JK305 | CB15N Δ*pbpA1* Δ*pbpC* Δ*pbpY* Δ*pbpZ* | - | Strobel *et al.* (2014) |
| KK1 | CB15N Δ*pbpX* | - | Strobel *et al.* (2014) |
| KK12 | CB15N Δ*pbp1A* Δ*pbpY* Δ*pbpZ* | - | Strobel *et al.* (2014) |
| KK33 | CB15N P_xyl_::P_xyl_-*venus-pbp1A* | - | Strobel *et al.* (2014) |
| LS3809 | CB15N Δ*mreB* P_xyl_::P_xyl_-*mreB* | - | Gitai *et al.* (2004) |
| LS4275 | CB15N Δ*mreC* P_xyl_::P_xyl_*-mreC* | - | Dye *et al.* (2005) |
| MAB223 | CB15N P*_xyl_*::P_xyl_-*mreC-mCherry* | Integration of pMAB66 in CB15N | This study |
| MAB233 | CB15N Δ*CCNA_03431* | In-frame deletion of CCNA_03431 in CB15N using pMAB70 | This study |
| MAB238 | CB15N *mreB::mreB^sw^* | In-frame integration of pMAB64 in CB15N | This study |
| MAB239 | CB15N Δ*CCNA_03856* | In-frame deletion of CCNA_03856 in CB15N using pMAB60 | This study |
| MAB244 | CB15N *pbp2::gfp-pbp2* | In-frame integration of *gfp-pbp2* in CB15N using pMAB67 | This study |
| MAB247 | CB15N P_xyl_::P_xyl_*-crbA-mCherry* | Integration of pMT719 in CB15N | This study |
| MAB248 | CB15N Δ*CCNA_03031* | In-frame deletion of CCNA_03031 in CB15N using pMAB62 | This study |
| MAB250 | CB15N Δ*CCNA_02863* Δ*CCNA_00354* Δ*CCNA_03031* | In-frame deletion of CCNA_03031 in AZ89 using pMAB62 | This study |
| MAB251 | CB15N Δ*CCNA_03856* Δ*CCNA_03431* | In-frame deletion of CCNA_03431 in MAB239 using pMAB70 | This study |
| MAB315 | CB15N Δ*crbA* P_xyl_::P_xyl_-*venus-mreB* | Integration of pMT1003 in AM376 | This study |
| MAB316 | CB15N Δ*crbA* P_xyl_::P_xyl_*-dipM-mCherry* | Integration of pMT795 in AM376 | This study |
| MAB317 | CB15N Δ*crbA* P_xyl_::P_xyl_-*bacA-cfp* | Integration of pMT810 in AM376 | This study |
| MAB318 | CB15N Δ*bacA* P_xyl_::P_xyl_-*crbA-mCherry* | Integration of pMT719 in JK55 | This study |
| MAB319 | CB15N Δ*bacA* P_xyl_::P_xyl_*-venus-mreB* | Integration of pMT1003 in JK55 | This study |
| MAB320 | CB15N Δ*dipM* P_xyl_::P_xyl_*-bacA-cfp* | Integration of pMT810 in MT258 | This study |
| MAB 321 | CB15N Δ*dipM* P_xyl_::P_xyl_-*crbA-mCherry* | Integration of pMT719 in MT258 | This study |
| MAB322 | CB15N Δ*dipM*  P_xyl_::P_xyl_-*venus-mreB* | Integration of pMT1003 in MT258 | This study |
| MAB323 | CB15N Δ*sdpAB* P_xyl_::P_xyl_-*bacA-cfp* | Integration of pMT810 in AZ22 | This study |
| MAB324 | CB15N Δ*sdpAB* P_xyl_::P_xyl_*-crbA-mCherry* | Integration of pMT719 in AZ22 | This study |
| MAB325 | CB15N Δ*ldpA* P_xyl_::P_xyl_-*venus-mreB* | Integration of pMT1003 in AM364 | This study |
| MAB326 | CB15N Δ*ldpA* P_xyl_::P_xyl_-*dipM-mCherry* | Integration of pMT795 in AM364 | This study |
| MAB327 | CB15N Δ*ldpA* P_xyl_::P_xyl_-*crbA-mCherry* | Integration of pMT719 in AM364 | This study |
| MAB328 | CB15N Δ*ldpA* P_xyl_::P_xyl_-*bacA-cfp* | Integration of pMT810 in AM364 | This study |
| MAB329 | CB15N Δ*sdpAB* P_xyl_::P_xyl_-*venus-mreB* | Integration of pMT1003 in AZ22 | This study |
| MAB330 | CB15N Δ*bacA* P_xyl_::P_xyl_-*dipM-mCherry* | Integration of pMT795 in JK55 | This study |
| MAB331 | CB15N Δ*sdpAB* P_xyl_::P_xyl_-*dipM-mCherry* | Integration of pMT795 in AZ22 | This study |
| MAB360 | CB15N Δ*dipM* P_xyl_::P_xyl_-*dipM-mCherry* | - | Zielinska *et al.* (2017) |
| MAB386 | CB15N Δ*amiC* P_xyl_::P*_xyl_*-*amiC* | - | Zielinska *et al.* (2017) |
| MAB389 | CB15N P*_xyl_*::P_xyl_-*ldtD-mCherry* | Integration of pMAB133 in CB15N | This study |
| MAB390 | CB15N P_xyl_::P_xyl_-*ldtX-mCherry* | Integration of pMAB134 in CB15N | This study |
|  |  |  |  |

**Table S6. *C. crescentus* strains used in this study (continued).**

| **Strain** | **Genotype/description** | **Construction** | **References** |
| --- | --- | --- | --- |
| MAB404 | CB15N P_xyl_::P_xyl_-*rodA* | Integration of pMAB147 in CB15N | This study |
| MAB405 | CB15N P_xyl_::P_xyl_-*gfp-rodA* | Integration of pMAB148 in CB15N | This study |
| MAB407 | Δ*rodA* P_xyl_::P*_xyl_-rodA* | In-frame deletion of *rodA* in MAB404 using pMAB146 | This study |
| MT174 | CB15N *parB::gfp-parB* | - | Thanbichler *et al.* (2006) |
| MT199 | CB15N P_van_::P_van_-*ftsZ-yfp* | - | Thanbichler *et al.* (2006) |
| MT257 | CB15N Δ*bacA* | - | Kühn *et al.* (2010) |
| MT258 | CB15N Δ*dipM* | - | Möll *et al.* (2010) |
| MT260 | CB15N *bacA::bacA-cfp* | - | Kühn *et al.* (2010) |
| MT278 | CB15N P_xyl_::P_xyl_-*venus-pbpZ* | - | Strobel *et al.* (2014) |
| MT279 | CB15N P_xyl_::P_xyl_-*venus-pbpC* | - | Strobel *et al.* (2014) |
| MT286 | CB15N Δ*pbpC* | - | Kühn *et al.* (2010) |
| MT309 | CB15N P_xyl_::P_xyl_-*venus-mreB* | Integration of pMT1003 in CB15N | This study |
| SW30 | CB15N P_xyl_::P_xyl_-*stpB-mCherry* |  | Schlimpert *et al.* (2012) |
| SW51 | CB15N Δ*stpA* Δ*stpB* | - | Schlimpert *et al.* (2012) |
| WS056 | CB15N Δ*pbpY* Δ*pbp1A* Δ*pbpC* Δ*pbpZ* P_xyl_::P_xyl_-*pbpX* | - | Strobel *et al.* (2014) |
|  |  |  |  |

**References**

Alyahya SA, Alexander R, Costa T, Henriques AO, Emonet T, Jacobs-Wagner C. RodZ, a component of the bacterial core morphogenic apparatus (2009) Proc Natl Acad Sci USA 106: 1239-1244.

Evinger M, Agabian N. Envelope-associated nucleoid from *Caulobacter crescentus* stalked and swarmer cells (1977) J Bacteriol 132: 294-301.

Dye NA, Pincus Z, Theriot JA, Shapiro L, Gitai Z. (2005) Two independent spiral structures control cell shape in *Caulobacter*. Proc Natl Acad Sci USA 102: 18608-18613.

Gitai Z, Dye N, Shapiro L. An actin-like gene can determine cell polarity in bacteria (2004) Proc Natl Acad Sci USA 101: 8643-8648.

Kühn J, Briegel A, Mörschel E, Kahnt J, Leser K, Wick S, et al. (2010) Bactofilins, a ubiquitous class of cyto­skeletal proteins mediating polar localization of a cell wall synthase in *Caulobacter crescentus*. EMBO J 29: 327-339.

Möll A, Schlimpert S, Briegel A, Jensen GJ, Thanbichler M. (2010) DipM, a new factor required for peptido­glycan remodelling during cell division in *Caulobacter crescentus*. Mol Microbiol 77: 90-107.

Schlimpert S, Klein EA, Briegel A, Hughes V, Kahnt J, Bolte K, et al. (2012) General protein diffusion barriers create compartments within bacterial cells. Cell 151: 1270-1282.

Strobel W, Möll A, Kiekebusch D, Klein KE, Thanbichler M. (2014) Function and localization dynamics of bifunc­tional penicillin-binding proteins in *Caulobacter crescentus*. J Bacteriol 196: 1627-1639.

Thanbichler M, Shapiro L. (2006) MipZ, a spatial regulator coordinating chromosome segregation with cell division in *Caulobacter*. Cell 126: 147-162.

Zielinska A, Billini M, Möll A, Kremer K, Briegel A, Izquierdo Martinez A, et al. (2017) LytM factors affect the recruitment of autolysins to the cell division site in *Caulobacter crescentus*. Mol Microbiol 106: 419-438.
