# Supplementary material for: A specialized MreB-dependent cell wall biosynthetic complex mediates the formation of stalk-specific peptidoglycan in Caulobacter crescentus
Source: PLoS Genet. 2019 Feb 1;15(2):e1007897. doi: 10.1371/journal.pgen.1007897 (PMC6373972; doi:10.1371/journal.pgen.1007897)
Supplement: S6 Table — (DOCX) [file pgen.1007897.s017.docx]

**Table S6.** **General plasmids used in this work.**

| **Plasmid** | **Description** | | **References** | |
| --- | --- | --- | --- | --- |
|  |  | |  | |
| pNPTS138 | *sacB*-containing suicide plasmid used for double homologous recombination, Kan^R^ | | M.R. Alley, unpublished | |
| pXVENN-1 | Integrating plasmid for the construction of N-terminal fusions to Venus under the control of P_xyl_**,** Strep/Spec^R^ | | Thanbichler *et al.* (2007) | |
| pXVENN-4 | Integrating plasmid for the construction of N-terminal fusions to Venus under the control of P_xyl_**,** Gen^R^ | | Thanbichler *et al.* (2007) | |
| pXCHYC-2 | Integrating plasmid for the construction of C-terminal fusions to mCherry under the control of P_xyl_**,** Kan^R^ | | Thanbichler *et al.* (2007) | |
| pXGFPN-4 | Integrating plasmid for the construction of N-terminal fusions to GFP under the control of P_xyl_**,** Gen^R^ | | Thanbichler *et al.* (2007) | |
| pMT795 | Integrating plasmid for the expression of the DipM-mCherry fusion under the control of the P_xyl_, Kan^R^ | | Möll *et al.* (2010) | |
| pMT810 | Integrating plasmid for the expression of the BacA-CFP fusions under the control of the P_xyl_, Kan^R^ | | Kühn *et al.* (2010) | |
|  | |  | |  |

**References**

Kühn J, Briegel A, Mörschel E, Kahnt J, Leser K, Wick S, et al. (2010) Bactofilins, a ubiquitous class of cyto­skeletal proteins mediating polar localization of a cell wall synthase in *Caulobacter crescentus*. EMBO J 29: 327-339.

Möll A, Schlimpert S, Briegel A, Jensen GJ, Thanbichler M. (2010) DipM, a new factor required for peptido­glycan remodelling during cell division in *Caulobacter crescentus*. Mol Microbiol 77: 90-107.

Thanbichler M, Iniesta AA, Shapiro L. (2007) A comprehensive set of plasmids for vanillate- and xylose-inducible gene expression in *Caulobacter crescentus*. Nucleic Acids Res 35: e137.
