# Supplementary material for: A specialized MreB-dependent cell wall biosynthetic complex mediates the formation of stalk-specific peptidoglycan in Caulobacter crescentus
Source: PLoS Genet. 2019 Feb 1;15(2):e1007897. doi: 10.1371/journal.pgen.1007897 (PMC6373972; doi:10.1371/journal.pgen.1007897)
Supplement: S7 Table — (DOCX) [file pgen.1007897.s018.docx]

**Table S7. Plasmids generated in this work.**

| **Plasmid** | **Description** | **Construction** |
| --- | --- | --- |
|  |  |  |
|  |  |  |
|  |  |  |
|  |  |  |
|  |  |  |
| pAM152 | pNPTS138 derivative for in-frame deletion of *crbA* | a) amplification of the CCNA_02243 flanking regions from CB15N chromosomal DNA using primers oAM265+oAM266 (upstream) and oAM267+oAM268 (downstream),  b) restriction of the upstream fragment with HindIII and EcoRI, restriction of the downstream fragment with EcoRI and NheI.  c) triple ligation with pNPTS138 cut with HindIII and NheI |
| pAZ11 | pNPTS138 derivative for in-frame deletion of *CCNA_00354* | a) amplification of the CCNA_00354 flanking regions from CB15N chromosomal DNA using primers oAZ19+oAZ20 (upstream) and oAZ21+oAZ22 (downstream),  b) restriction of the upstream fragment with PstI and EcoRI, restriction of the downstream fragment with EcoRI and NheI.  c) triple ligation with pNPTS138 cut with PstI and NheI |
| pAZ12 | pNPTS138 derivative for in-frame deletion of *CCNA_02863* | a) amplification of the CCNA_02863 flanking regions from CB15N chromosomal DNA using primers oAZ23+oAZ24 (upstream) and oAZ25+oAZ26 (downstream),  b) restriction of the upstream fragment with HindIII and EcoRI, restriction of the downstream fragment with EcoRI and NheI.  c) triple ligation with pNPTS138 cut with HindIII and NheI |
| pAZ26 | pNPTS138 derivative for in-frame deletion of *ldtD* | a) amplification of the CCNA_01579 flanking regions from CB15N chromosomal DNA using primers oAZ70+oAZ71 (upstream) and oAZ72+oAZ73 (downstream),  b) restriction of the upstream fragment with PstI and EcoRI, restriction of the downstream fragment with EcoRI and NheI.  c) triple ligation with pNPTS138 cut with PstI and NheI |
| pAZ30 | pNPTS138 derivative for in-frame deletion of *ldtX* | a) amplification of the CCNA_03860 flanking regions from CB15N chromosomal DNA using primers oAZ81+oAZ82 (upstream) and oAZ83+oAZ84 (downstream),  b) restriction of the upstream fragment with HindIII and EcoRI, restriction of the downstream fragment with EcoRI and NheI.  c) triple ligation with pNPTS138 cut with HindIII and NheI |
| pMT719 | pXCHYC-2 derivative bearing *crbA-mCherry* | a) amplification of CCNA_02243 from CB15N chromosomal DNA using primers oMT657+oMT658, restriction with NdeI and SacI b) ligation with pXCHYC-2 cut with NdeI and SacI |
| pMT1003 | pXVENN-1 derivative bearing *venus-mreB* | a) amplification of CCNA_01612 from CB15N chromosomal DNA using primers oMT1107+oMT1112, restriction with BglII and NheI b) ligation with pXVENN-1 cut with BglII and NheI |
| pMAB60 | pNPTS138 derivative for in-frame deletion of *CCNA_03856* | a) amplification of the CCNA_03856 flanking regions from CB15N chromosomal DNA using primers oMAB235+oMAB218 (upstream) and oMAB219+oMAB236 (downstream) b) double-joint PCR with oMAB235 and oMAB236 and restriction of the PCR product with EcoRI and HindIII,  c) ligation with pNPTS138 cut with EcoRI and HindIII |
| pMAB62 | pNPTS138 derivative for in-frame deletion of *CCNA_03031* | a) amplification of the CCNA_03031 flanking regions from CB15N chromosomal DNA using primers oMAB245+oMAB246 (upstream) and oMAB247+oMAB248 (downstream)  b) double-joint PCR with oMAB245 and oMAB248 and restriction of the PCR product with EcoRI and HindIII,  c) ligation with pNPTS138 cut with EcoRI and HindIII |
| pMAB64 | pNPTS138 derivative for in-frame replacement of *mreB* with *mreB^sw^* | a) amplification of the CCNA_01612 coding regions from CB15N chromosomal DNA using primers oMAB231+oMAB206 (upstream coding region until 227 codon) and oMAB209+oMAB233 (downstream coding region from 228 codon)  b) amplification of the mCherry coding region from pXCHYC-2 using primers oMAB207+oMAB208 bearing appropriate linkers and compatible regions with the CCNA_02243 fragments  c) triple-joint PCR with oMAB231 and oMAB233 of the upstream, doawnstream and mCherry fragments and restriction of the PCR product with EcoRI and HindIII,  d) ligation with pNPTS138 cut with EcoRI and HindIII |
| pMAB65 | pXGFPN-4 derivative bearing *gfp-pbp2* | a) amplification of CCNA_01615 from CB15N chromosomal DNA using primers oMAB221+oMAB222, restriction with KpnI and NheI b) ligation with pXGFPN-2 cut with KpnI and NheI |
| pMAB66 | pXCHYC-2 derivative bearing *mreC-mCherry* | a) amplification of CCNA_01613 from CB15N chromosomal DNA using primers oMAB185+oMAB186, restriction with NdeI and KpnI b) ligation with pXCHYC-2 cut with NdeI and KpnI |
| pMAB67 | pNPTS138 derivative for in-frame replacement of *pbp2* with *gfp-pbp2* | a) amplification of the CCNA_01615 upstream flanking region from CB15N chromosomal DNA using primers oMAB241+oMAB242 and the GFP with the first 685bp of CCNA_01615 coding region from pMAB65 with primers oMAB243+oMAB244  b) double-joint PCR with oMAB241 and oMAB244 of the upstream and downstream fragments and restriction of the PCR product with EcoRI and HindIII,  d) ligation with pNPTS138 cut with EcoRI and HindIII |
| pMAB70 | pNPTS derivative for in-frame deletion of *CCNA_03431* | a) PCR amplification of the CCNA_03431 flanking regions from chromosomal DNA using primers oMAB215+oAMAB202 (upstream) and oMAB203+oMAB204 (downstream)  b) double-joint PCR with oMAB215 and oMAB204 of the upstream and downstream fragments and restriction of the PCR product with PstI and EcoRI,  d) ligation with pNPTS138 cut with PstI and EcoRI |
|  |  |  |

**Table S8. Plasmids generated in this work (continued).**

| **Plasmid** | **Description** | **Construction** |
| --- | --- | --- |
| pMAB133 | pXCHYC-2 derivative bearing *ldtD-mCherry* | a) amplification of CCNA_01579 from CB15N chromosomal DNA using primers oMAB383+oMAB384  b) Gibson assembly with pXCHYC-2 cut with NdeI and KpnI |
| pMAB134 | pXCHYC-2 derivative bearing *ldtX-mCherry* | a) amplification of CCNA_03860 from CB15N chromosomal DNA using primers oMAB385+oMAB386 b) Gibson assembly with pXCHYC-2 cut with NdeI and KpnI |
| pMAB146 | pNPTS138 derivative for in-frame deletion of *rodA* | a) amplification of rodA flanking regions from CB15N chromosomal DNA using primers oMAB402/oMAB403 (upstream) and oMAB404/oMAB405 (downstream)  b) Gibson assembly with pNTPS138 cut with EcoRI and HindIII |
| pMAB147 | pXCHYC-4 derivative bearing *rodA* | a) amplification of *rodA* from CB15N chromosomal DNA using primers oMAB160+oMAB161  b) ligation with pXCHYC-4 cut with NdeI and NheI |
| pMAB148 | pXGFPN-4 derivative bearing *venus-rodA* | a) amplification of *rodA* from CB15N chromosomal DNA using primers oMAB406+oMAB161 b) Gibson assembly with pXGFPN-4 cut with KpnI and NheI |
|  |  |  |
