# Supplementary material for: A specialized MreB-dependent cell wall biosynthetic complex mediates the formation of stalk-specific peptidoglycan in Caulobacter crescentus
Source: PLoS Genet. 2019 Feb 1;15(2):e1007897. doi: 10.1371/journal.pgen.1007897 (PMC6373972; doi:10.1371/journal.pgen.1007897)
Supplement: S8 Table — (DOCX) [file pgen.1007897.s019.docx]

**Table S8.** **Oligonucleotides used in this work.**

| **ID** | **Oligonucleotide** | | **Sequence** (5’ to 3’) ^1^ | | **Restriction site** |
| --- | --- | --- | --- | --- | --- |
| oAM265 | CC2161_265f | | TAT**AAGCTT**GACTTGGCGGCGGCGACTTGATCTTC | | HindIII |
| oAM266 | CC2161_266r | | TA**GAATTC**GGTAAGCCTGGCGAACATGAAGCGAC | | EcoRI |
| oAM267 | CC2161_267f | | TA**GAATTC**ATTGTGCTGAAGCCGTAGGCGAAAC | | EcoRI |
| oAM268 | CC2161_268r | | TTTTT**GCTAGC**AAGGCCGTGATTGAGAAGCTGGTGC | | NheI |
| oAZ19 | CCNA_00354-1f | | TA**CTGCAG**GCGCTGCGTATCGTGCCGTGC | | PstI |
| oAZ20 | CCNA_00354-2r | | TA**GAATTC**AAAAGAGTGTTTATCACGCTC | | EcoRI |
| oAZ21 | CCNA_00354-3f | | TA**GAATTC**CCCAACAACAAGCCGACCGCG | | EcoRI |
| oAZ22 | CCNA_00354-4r | | TATA**GCTAGC**AGGCAGGCGCCGCGCATGTCG | | NheI |
| oAZ23 | CCNA_02863-1f | | TATA**AAGCTT**CGGCGGCTGGAGTCCCGGCGC | | HindIII |
| oAZ24 | CCNA_02863-2r | | TA**GAATTC**GGATTGCGCGCCCCCGCCGCT | | EcoRI |
| oAZ25 | CCNA_02863-3f | | TA**GAATTC**TCAGGAGCGCGCACCTCAGGCTGA | | EcoRI |
| oAZ26 | CCNA_02863-4r | | TATA**GCTAGC**CGGCCGATCCCGTCGATCGGG | | NheI |
| oAZ70 | CCNA_01579-1 | | TA**CTGCAG**AAGAAGAAGGCCATCAAGTC | | PstI |
| oAZ71 | CCNA_01579-2 | | TA**GAATTC**GGCGCCGCAGGTTCTCGTAAA | | EcoRI |
| oAZ72 | CCNA_01579-3 | | TA**GAATTC**CGTGATCTGGTGCAGCGTAT | | EcoRI |
| oAZ73 | CCNA_01579-4 | | TATA**GCTAGC**TGGAACGGCCCCTTGCTCTG | | NheI |
| oAZ82 | CCNA_03860-1 | | TATA**AAGCTT**GCGGAACGGCTTGGTCACAT | | HindIII |
| oAZ83 | CCNA_03860-2 | | TA**GAATTC**GCGGCCGTCAGCATGGGCTC | | EcoRI |
| oAZ84 | CCNA_03860-3 | | TA**GAATTC**CCCGGCGACGCCGTCCAGATT | | EcoRI |
| oAZ85 | CCNA_03860-4 | | TATA**GCTAGC**CGTGAGGCGGTCGGCTTTTT | | NheI |
| oMT657 | CC2161-uni | | TTAATT**CATATG**TTCGCCAGGCTTACCTCCGCCC | | NdeI |
| oMT658 | CC2161-rev | | TA**GAGCTC**CCGGCTTCAGCACAATGCAGGGCATG | | SacI |
| oMT1107 | mreb-for | | TTT**AGATCT**ATGTTCTCTTCCCTTTTCGGCGTGATCTCG | | BglII |
| oMT1112 | mreB-rev3 | | TTAA**GCTAGC**CTAGGCCAGCGTGGATTCCAGGACGC | | NheI |
| oMAB160 | RodAF-NdeI | | ATAT**CATATG**ACCCTGAGCGGCGGCCTTTC | | NdeI |
| oMAB161 | RodAF-NheI | | ATAT**GCTAGC**TCAGACCAGCGAGCCCTTGCCG | | NheI |
| oMAB235 | CC3740UpF(EcoRI) | | ATAT**GAATTC**GGGCCCAAAGCCCGCTGCC | | EcoRI |
| oMAB218 | CC3740UpR | | TTCGGCGTCAGCCGATAAAGACGCGCCAGCGAGGTGTAGGCCAGG | | - |
| oMAB219 | CC3740DowF | | CCTGGCCTACACCTCGCTGGCGCGTCTTTATCGGCTGACGCCGAA | | - |
| oMAB236 | CC3740DowR(HindIII) | | ATAT**AAGCTT**CCCTGGATTGTTCTGAAAGGGTGAAACC | | HindIII |
| oMAB245 | CCNA03031UpF-BamHI | | ATAT**GGATCC**CCGCCCACGTCCTGGCGCT | | BamHI |
| oMAB246 | CCNA03031UpR | | CGATACCCCAGCACCCCGCCCAGGGTGAGGCGGCGGTCG | | - |
| oMAB247 | CCNA03031DoF | | CGACCGCCGCCTCACCCTGGGCGGGGTGCTGGGGTATCG | | - |
| oMAB248 | CCNA03031DR-HindIII | | ATAT**AAGCTT**GCACGGGCCCCGGCCTGT | | HindIII |
| oMAB231 | MreBUpF (EcoRI) | | ATAT**GAATTC**ATGTTCTCTTCCCTTTTCGGCGTGA | | EcoRI |
| oMAB206 | MreB-mChe UpR | | TGTTATCCTCCTCGCCCTTGCTCACGCTCGAGCCAGAGTCGGCCGGCGCGCGGG | | - |
| oMAB207 | mChe-MreB-F | | CCCGCGCGCCGGCCGACTCTGGCTCGAGCGTGAGCAAGGGCGAGGAGGATAACA | | - |
| oMAB208 | mChe-MreB-R | | ACGTCGATCGACAGACCTTCGCCGCCCGGCGCGCCAGACTTGTACAGCTCGTCCATGCCGCC | | - |
| oMAB209 | MreB-mChe-DF | | GGCGGCATGGACGAGCTGTACAAGTCTGGCGCGCCGGGCGGCGAAGGTCTGTCGATCGACGT | | - |
| oMAB233 | MreB-DR(HindIII) | | ATAT**AAGCTT**CTAGGCCAGCGTGGATTCCAGGAC | | HindIII |
| oMAB221 | Pbp2F(KpnI) | | ATAT**GGTACC**AGCGAACCGTCCATCTTCTTTTTCGAG | | KpnI |
| oMAB222 | Pbp2R(NheI) | | ATAT**GCTAGC**TCATGTCTGGCCTCCCTGCGGAGTTG | | NheI |
| oMAB185 | MreCF(NdeI) | | ATAT**CATATG**CGCTTTCGTGAAGGTCCGCTGG | | NdeI |
| oMAB186 | MreCR(KpnI) | | ATAT**GGTACC**TCGGGGCGCTCCCGTCTGAG | | KpnI |
| oMAB241 | PBP2UPST-UF(EcoRI) | | ATAT**GAATTC**CCTCGATCCTGTCGAACCCCGTCT | | EcoRI |
| oMAB242 | PBP2UPSTGFP-UR | | AGCTCCTCGCCCTTGCTCACCATCGCCTCACCGGAACCGCACG | | - |
| oMAB243 | PBP2UPSTGFP-DF | | CGTGCGGTTCCGGTGAGGCGATGGTGAGCAAGGGCGAGGAGCT | | - |
| oMAB244 | PBP2-DR(HindIII) | | ATAT**AAGCTT**TGGCGCCGGCGCGCCC | | HindIII |
| oMAB215 | CC3322UpF(PstI) | | ATAT**CTGCAG**AGACCGTCTCGGGAATCGGCCC | | PstI |
| oMAB202 | CC3322UpR | | CTGATCCCGGCCTGGGCCTTCAGAACAAGAAAGACGCGACGATCCATAGC | | - |
| oMAB203 | CC3322DoF | | GCTATGGATCGTCGCGTCTTTCTTGTTCTGAAGGCCCAGGCCGGGATCAG | | - |
| oMAB204 | CC3322DoR(EcoRI) | | ATAT**GAATTC**CCGCCGAACCTGGAGGGCCAT | | EcoRI |
| oMAB383 | CCNA_01579-NdeI | | TCGAGTTTTGGGGAGACGAC**CATATG**CGATTTACGAGAACCTGCG | | NdeI |
| oMAB384 | CCNA_01579-KpnI | | TCCGGAGCTCGAGATCTTAA**GGTACC**GAGTGCGGCGATACGCTGCA | | KpnI |
| oMAB385 | CCNA_03860-pXC-F | | TCGAGTTTTGGGGAGACGAC**CATATG**CTGTTTCGAGCCCATGCTGAC | | NdeI |
| oMAB386 | CCNA_03860 pXC-KpnI-R | | TCTCCGGAGCTCGAGATCTTAA**GGTACC**GTCCGCGAGAATCTGGACGGCGT | | KpnI |
| oMAB402 | RodAUpF(EcoRI) | | CGGCCGAAGCTAGC**GAATTCC**GTAATGGCATGGCCGCCGT | | EcoRI |
| oMAB403 | RodAUPrev | | CACGCGGACGGCCTGGATGAACTTGATGGTCGGCCGGT | | - |
| oMAB404 | RodADownF | | ACCGGCCGACCATCAAGTTCATCCAGGCCGTCCGCGTG | | - |
| oMAB405 | RodAdown_rev(HindIII) | | GCCGGCTGGCGCC**AAGCTT**GCCCAGCTTGGACAGAATCGC | | HindIII |
| oMAB406 | RodAF-KpnI | | ATAT**GGTACC**ACCCTGAGCGGCGGCCT | | KpnI |
|  |  |  | |  | |
|  |  |  | |  | |
|  |  |  | |  | |
|  |  |  | |  | |
|  |  |  | |  | |

^1^ Restriction sites are indicated in boldface.
